# Supplementary material for: Three-dimensional alteration of neurites in schizophrenia
Source: Transl Psychiatry. 2019 Feb 12;9:85. doi: 10.1038/s41398-019-0427-4 (PMC6372695; doi:10.1038/s41398-019-0427-4)
Supplement: Supplementary file 3 — Suppl. Methods, Figs, Table S4, clean [file 41398_2019_427_MOESM3_ESM.docx]

Supplementary Information of:

**Three-dimensional alteration of neurites in schizophrenia**

Ryuta Mizutani^1^*, Rino Saiga^1^, Akihisa Takeuchi^2^, Kentaro Uesugi^2^, Yasuko Terada^2^, Yoshio Suzuki^3^, Vincent De Andrade^4^, Francesco De Carlo^4^, Susumu Takekoshi^5^, Chie Inomoto^5^, Naoya Nakamura^5^, Itaru Kushima^6^, Shuji Iritani^6^, Norio Ozaki^6^, Soichiro Ide^7^, Kazutaka Ikeda^7^, Kenichi Oshima^7^, Masanari Itokawa^7^, and Makoto Arai^7^

^1^Department of Applied Biochemistry, Tokai University, Hiratsuka, Kanagawa 259-1292, Japan

^2^Japan Synchrotron Radiation Research Institute (JASRI/SPring-8), Sayo, Hyogo 679-5198, Japan.

^3^Graduate School of Frontier Sciences, University of Tokyo, Kashiwa, Chiba 277-8561, Japan

^4^Advanced Photon Source, Argonne National Laboratory, Lemont, IL 60439, USA.

^5^Tokai University School of Medicine, Isehara, Kanagawa 259-1193, Japan.

^6^Graduate School of Medicine, Nagoya University, Nagoya, Aichi 466-8550, Japan.

^7^Tokyo Metropolitan Institute of Medical Science, Setagaya, Tokyo 156-8506, Japan.

*mizutanilaboratory@gmail.com

**Index**

Supplementary Materials and Methods ... p. 2-10

Supplementary Figure S1 ... p. 11

Supplementary Figure S2 ... p. 12-18

Supplementary Figure S3 ... p. 19-31

Supplementary Figure S4 ... p. 32-42

Supplementary Figure S5 ... p. 43

Supplementary Figure S6 ... p. 44

Supplementary Figure S7 ... p. 45-46

Supplementary Figure S8 ... p. 47

Supplementary Figure S9 ... p. 48

Supplementary Table S4 ... p. 49

Supplementary Video captions ... p. 49

Supplementary Tables S1-S3 are provided separately.

**Supplementary Materials and Methods**

**Microtomography**

Overall tissue structures were visualized with simple projection microtomography at the BL20XU^1^ beamline of SPring-8 using monochromatic radiation at 12 keV (Supplementary Table S2). Absorption contrast images of the N3 tissues were recorded with a CMOS-based imaging detector (ORCA-Flash2.8, Hamamatsu Photonics, Japan) during the beamtime of 2011.12. The layer V position (Supplementary Table S1) of the N3 tissues was estimated from the obtained image. Absorption contrast images of other tissues were recorded with a CMOS-based imaging detector (ORCA-Flash4.0, Hamamatsu Photonics, Japan) during the beamtime of 2013.1 or later. The obtained images along with Nissl sections were used for estimating the position of layer V (Supplementary Table S1), as reported previously.^2^ Nissl sections of paraffin-embed tissues showed shrinkage compared with the microtomographic images of the resin-embed tissues. Examples of microtomographic images and Nissl sections are shown in Supplementary Fig. S8. The data collection conditions are summarized in Supplementary Table S2.

**Data collection procedure for nanotomography**

In the nanotomography experiment, the data of each sample was collected as follows. First, the upper and lower ends of the sample were searched in order to determine the positions of the pial surface and tissue bottom. Then the sample was moved so as to approximately place the bottom of the internal pyramidal layer (layer V) in the viewing field. It has been reported that the spine density in layer V of the frontal cortex shows no significant difference between schizophrenia and control cases.^3^ Therefore, it should be difficult to distinguish a disease case from a control case only from their layer V images. This eliminated possible biases in the model building. The sample stage was first moved horizontally and then vertically to search for a neuron having an apical dendrite. The first neuron found in the search was placed in the center of the viewing field. Since most neurons were longer than the height of the detector viewing field, multiple datasets were collected by shifting the sample along the vertical axis to cover the entire neuronal structure. After each dataset was collected, the sample was shifted along the vertical axis toward the pial direction to search for the next neuron. This process was repeated until the predetermined beamtime for one sample was used up. In some cases, the search was also performed from the upper end of layer V toward the white matter.

Radiation damage appears as sample drifts or deformations in the reconstructed tomograms depending on the photon flux, exposure per frame, or frames per dataset. In this study, the experiment conditions were set so as not to induce any damage to the sample. Tomograms without the sample damage ensured that the possible structural changes due to the x-ray exposure were smaller than the resolution.

**Dataset coding and analysis**

Nanotomography datasets were analyzed with the role allotment of data management to RS and data analysis to RM. In the preliminary stage of the study, the data manager provided seven datasets of S1A, S1D, N2A, N3A, N3C, N3D, and N4D (Supplementary Table S3) together with their case names to the analyst. The analyst used these data to establish analysis algorithms and examine the methodological feasibility of the procedures described below. The data manager and the analyst had no previous information regarding the differences then to be found between the cases. In this stage, the analyst identified differences in the distributions of the spine curvatures between cases, although differences in the neurites were not identified because of lack of data. The obtained results were not disclosed to the data manager.

In the next stage of the study, the data manager provided the rest of the datasets to the data analyst in several batches without the case information in order to eliminate human biases in the model building. Although the voxel sizes differed depending on the experiment date, the same voxel size of 48.3 nm was used throughout the analysis, because the experiment dates were not disclosed to the data analyst. The data analyst analyzed the structure and returned the number of spines for each dataset to the data manager without acknowledging the kind of structure represented with that number. The provided number was only designated as "a summable indicator of the amount of analysis". No other results were disclosed to the data manager. The data manager sorted the number for each case to manage the progress of the study and to average the amount of analysis between cases. The sorted results were not returned to the data analyst. Datasets to be further analyzed were chosen from the data stock and provided to the analyst without the case information. In the final nanotomography experiments in October 2017, the amount of analysis was used for choosing samples for the data collection. The data manager chose two schizophrenia (S2 and S3) and two control (N1 and N3) cases. The reconstructed tomographic datasets were coded and provided to the data analyst. The binned voxel size of 52.0 nm was used for these final round data.

After the analyst finished building the model for all of the datasets, the data manager disclosed the experiment date of each dataset to the analyst to correct the voxel size. After the voxel size was corrected, all coordinate files of the tissue structure models in Protein Data Bank format were locked down. Then the case information was disclosed to the data analyst. Two dummy datasets unrelated to this study were included in order to shuffle datasets of the APS beamtime of 2016.6. All datasets except the two dummy sets were used in the subsequent analysis. The data analyst calculated the geometric parameters of the obtained models to analyze the structures.

There was a possibility of structural differences arising from differences in the experimental conditions. In order to examine this possibility, we performed preliminary analyses of the obtained geometric parameters prior to the analysis reported in the main text. We calculated the mean neurite curvature and the mean neurite torsion of each dataset according to the method described below and analyzed their differences between nanotomography beamtimes by using a Kruskal-Wallis test. The obtained *p*-values showed that structural differences between the beamtimes are not significant (*p* = 0.61 for neurite curvature and *p* = 0.40 for neurite torsion). In contrast, when the datasets were divided into schizophrenia and control groups, the curvature difference became highly significant (*p* = 2.2 × 10^-6^ for curvature and *p* = 0.064 for torsion). These results indicated that the neurite curvature is significantly different between schizophrenia and control cases, and that this curvature difference cannot be ascribed to differences in the experimental conditions between nanotomography beamtimes. Therefore, in the further analyses reported in the main text, a total of 55 geometric data from the four schizophrenia and four control cases were grouped according to the case information and used for the geometric analysis.

In the course of these structural analyses, the data analyst found little allowance to introduce human biases into the model building. This is because (1) most parts of the models were automatically generated by a defined algorithm, (2) a Cartesian coordinate model can be built only in accordance with the three-dimensional image, since it is difficult to place models arbitrarily in three-dimensional space, and (3) the obtained model was refined with conjugate gradient minimization of a target function that represents the model fitness to the three-dimensional image. Indeed, crystal structures of a huge number of macromolecules have been built without data coding even when native and ligand-bound forms were analyzed to compare their structures. Therefore, we suggest that the data coding procedure described above was not essential for identifying differences of neuronal structures between cases, though we paid much attention to the data coding.

**Cartesian coordinate models**

Cartesian coordinate models were built in four steps: (1) manual assignment of large structural constituents such as somata and blood capillary vessels, (2) automatic tracing to build a computer generated model and its subsequent refinement, (3), examination of the entire three-dimensional image and manual intervention to modify the working model, and (4) final structural refinement. This model building procedure, as well as structural analysis described below, was implemented in the MCTrace software available from https://mizutanilab.github.io under the BSD 2-Clause License.

The structural model built with this procedure is composed of nodes. The position of the node is represented with a three-dimensional Cartesian coordinate. Each node has a node name, such as A001. Adjacent nodes composing the same structural constituent (cell, neurite, or blood capillary vessel) are connected to form an inter-node edge. A radius is also assigned to the node to represent the size of the neurite or soma. Nodes of the same constituent are organized into a group, which is defined with the group category and its number, such as PYR 1 for pyramidal neuron 1. Therefore, each node has a node name, group name, group number, coordinate, and radius. The following procedure was designed to determine these parameters for each node.

First, the entire three-dimensional image was loaded into the software to build large structural constituents. In this step, voxels in 3 × 3 × 3 bins were averaged, and the resultant three-dimensional map of each 50 × 50 × 50 μm volume was sequentially examined. Neuronal somata and blood capillary vessels were modelled manually by placing and connecting nodes in the three-dimensional map. Each soma was observed as a large structure with a hollow sphere of the cellular nucleus. The node at the cellular nucleus was defined as the soma node and labeled. Capillary blood vessels were observed as luminal structures with partitions, presumably showing blood cells in the vessel. Apical or basal dendrites with diameters approximately larger than 2 μm were also built in this step. The initial model of dataset N2C is shown in Fig. 1b. This step is essential for the following automatic model generation, since large structures should be masked to facilitate a computerized search.

In the second step, neurites and dendritic spines were automatically modelled by using gradient vector flow^4^ and Sobel filter^5^ algorithms. This step is composed of two parts: start-point search and intensity tracing from the start point. First, an envelope of the initial model was defined with node spheres and cylinders connecting the nodes. Voxels within the envelope of the initial model were excluded from the automatic model building. The image was loaded with 2 × 2 × 2 binning in order to improve the signal-to-noise ratio of the image, since the spatial resolution is retained even after the binning. When the memory was insufficient to load the entire map, the three-dimensional image was divided into several horizontal sections and the following procedure was repeated for each section automatically. The intensity threshold for the model building was defined to be approximately 1.5˗2.5 times the standard deviation (1.5˗2.5 σ) of the voxel intensity depending on the noise level. The start-point search was performed by calculating the gradient vector flow.^4^ Every grid (~100 nm) position in the three-dimensional image was scored with a gradient vector flow and ranked. Then the coordinates of the top-20000 scores were sorted as start-point candidates to place a node. Hereafter, this start node is designated the "parent node". The Cartesian coordinate of the parent node was refined to place the node at the intensity-weighed center of the local structure. Its radius was determined from a sphere in which 90% of the volume had intensities higher than the threshold. In order to determine the trace direction, every vector from the parent node was evaluated with a three-dimensional Sobel filter.^5^ A child node was placed along the best-scored vector at a distance of 0.8–1.5 times the radius of the parent node. The tracing procedure was stopped if (1) the Sobel-filter score at the child node became lower than a predefined threshold, (2) the child node encountered other nodes, (3) the child node ran into a large structure such as soma, or (4) radius of the child node was two times larger or smaller than the mean radius of the five previous nodes. Then, the child node was used as a parent node of the next step and the tracing procedure was repeated until the stop condition was reached. When the tracing procedure stopped but only one direction from the original start node had been built, the opposite direction was also traced. If both directions were traced from the original start node, the next coordinate in the candidate list was used as the start node to repeat tracing. Most neurites were built in this step.

The obtained model was refined with conjugate gradient minimization of a target function:

$$E_{\mathrm{total}}=E_{\mathrm{CT}}+w_{\mathrm{bond}}\sum_{i} k_{\mathrm{bond}}\left( L_{i}-L_{i}^{\mathrm{ideal}} \right)^{2}+w_{\mathrm{angle}}\sum_{i} k_{\mathrm{angle}}\left( \theta_{i}-\theta_{i}^{\mathrm{ideal}} \right)^{2}$$

where *E*_CT_ denotes the observation term, *w* represents the weight of each restraint term, and *k* represents a force constant. *L* and *θ* represent the distance between two adjacent nodes and the angle formed by three consecutive nodes, respectively. To avoid large deviations from the pre-refinement structure, ideal values of the restraint terms were taken from the pre-refinement working model. Force constants of 100 μm^-2^ and 100 radian^-2^ were used in this study. The weights were set to be 1.0. The observation term *E*_CT_ was calculated with

$$E_{\mathrm{CT}}={-w}_{\mathrm{CT}}\frac{n_{\mathrm{node}}}{n_{\mathrm{voxel}}}\sum_{i\in\mathrm{model}} {k_{\mathrm{CT}}\rho}_{i}$$

where *n*_node_ represents the number of nodes in the model and *n*_voxel_ represents the number of voxels in the model envelope. *ρ_i_* represents voxel intensity scaled to 8-bit values. *n*_node_ was incorporated in order to balance the restraint terms, since the restraint terms are nearly proportional to the number of nodes in the model. The force constant *k*_CT_ was set to 2.0, and the weight *w*_CT_ to 1.0. Conjugate gradient minimization of the target function *E*_total_ was performed to refine the working model. The refinement process was monitored by calculating the mean intensity of the voxels within the model envelope. The automatically generated and refined model of dataset N2C is shown in Fig. 1c.

In the third step, the entire three-dimensional image was manually examined. The working model was edited to connect adjacent traces to each other, to remove traces built into ring artifacts, and to add traces into intensities which were not traced in the automatic model generation. These manual interventions were performed according to the method of protein crystallography.^6^ The working model was examined in a node-by-node manner using graphical interfaces implemented in the MCTrace software.^7^ A cage representation of the three-dimensional image was superposed on the working model so that the node coordinate and radius can be edited so as to fit the model to the observed image. The automatic tracing function described above was also used in the manual model building by placing one node manually and by invoking automatic tracing from that node. To facilitate the software operation, a dial input device (https://github.com/mizutanilab) was used along with a computer mouse. Most spines were modeled in this step, while some spines were traced in the automatic model generation.

Finally, the obtained working model was refined by minimizing the target function described above. In some cases, several nodes showed coordinate shifts larger than half the voxel size. Those largely shifted nodes were examined to clarify the reason for the shifts. In most cases, the shifted nodes moved toward a neighboring structure with strong voxel intensity, such as cell soma or blood vessels. This is because no repulsion term was included in the target function in this study. Those nodes were manually adjusted to fit the three-dimensional image. The final model of N2C is shown in Fig. 1d.

In crystallography, the Luzzati plot^8^ or sigma-A plot^9^ has been used for evaluating coordinate errors of models. Those errors are approximately 1/10th of the spatial resolution. In this study, models of thin neurites and spine necks were built into structures represented with a few grids (100–300 nm) of the 2 × 2 × 2 binned three-dimensional image. Model misalignments became discernible when coordinate displacements were larger than approximately half the voxel size (~50 nm). Such misalignments were corrected through the structural refinement described above. Therefore, the coordinate errors of the models of this study should be less than 50 nm. Although methods for calculating model fitness to the image and for evaluating coordinate errors of the model should be established, we roughly estimated the coordinate errors of the obtained models to be 20–30 nm, since the models of this study were built and refined according to the methods of protein crystallography, and the resolution of the three-dimensional image was 180–300 nm.

**Geometric analysis**

The geometric analysis was performed in three steps: cell typing, structure annotation, and geometric parameter calculation.

In the cell typing step, trace groups which have soma nodes were analyzed to determine their cell type. In this procedure, the soma node was defined as the first node and nodes adjacent to the first node were defined as second nodes. The third nodes were defined similarly. All branches ramifying from the first, second, or third node were tracked down to the node radius of 1 μm. These branches were defined as stems. Stems longer than 30 μm were defined as shafts. Most pyramidal neurons were found to have at least one shaft and a number of additional short stems. These correspond to apical and basal dendrites, respectively. Some neurons at image ends have a short stem that cannot be regarded as a shaft. Those neurons were manually inspected and assigned as pyramidal neurons if their morphology suggested the cell type. In the S4 case structures, two modified pyramidal neurons were found to point their apical dendrites toward the white matter. Interneurons were identified to have no shaft but several stems. Some neurons which cannot be categorized from their structures were designated as non-typed neurons. It has been reported that spindle-form neurons are found in the anterior cingulate cortex of the human brain.^10^ Such neurons should have two shafts and no stems. Although no spindle neuron fitting this definition was identified, the S3B and N1J datasets showed structures similar to spindle neurons. Neurites that cannot be tracked to their somata were also observed in the image. Most of them have spines. Since these neurites exhibited structures similar to pyramidal and interneuron neurites, they were designated as orphan neurties. Gliaform cells were identified from highly branching structures of eight or more stems and were easily determined from their three-dimensional image. Blood capillary vessels were also observed in the image, as described above. The number of tissue constituents in each structure is summarized in Supplementary Table S3.

In the next step, dendritic spines in the model were searched and annotated with labels. Neurites were divided into segments in this step. All neurites were tracked from the soma node. Orphan neurites that have no soma node were tracked from one end of the neurite. At a ramification node, all downstream neurites or spines connected to the ramification node were scored with

$$r\left( \frac{\cos\theta+1}{2} \right)$$

where *r* is the radius of the first node of the downstream neurite, and *θ* is the angle between the upstream and downstream vectors shown in Supplementary Fig. S9a. The downstream neurite that showed the maximum score was regarded as a continuing segment. Therefore, each apical dendrite was treated as one segment in this study. The other ramifying neurites were regarded as separate segments starting from the ramification node. A segment which met the following criteria was defined as dendritic spine: (1) angle *θ* larger than 30 degrees, (2) the segment length less than or equal to 8.0 μm, and (3) the minimum node radius less than or equal to 0.5 μm. Other segments were regarded as neurite ramifications. Although most spines were automatically identified with these criteria, all annotations were examined and edited according to the morphology using the model building interface described above. In this study, branched spines with multiple heads were divided into multiple spines by using the ramification score.

Finally, the geometric parameters were calculated from the annotated structures. The length of a neurite segment was defined as the sum of distances between nodes. Since dendritic spines are regarded as protrusions from the dendrite, the radius of the end node was added to the spine length and the radius of the stem node was subtracted from the length (Supplementary Fig. S9b).

The curvature *c_i_* at node *i* was calculated with:

$$c_{i}=\frac{\theta_{i}}{\frac{\left| \mathbf{p}_{\boldsymbol{i}} \right|+\left| \mathbf{q}_{\boldsymbol{i}} \right|}{2}}$$

where **p***_i_* is the upstream vector, **q***_i_* the downstream vector, and *θ_i_* the angle between vectors **p***_i_* and **q***_i_* (Supplementary Fig. S9c). The weighted-mean curvature *c* of a neurite segment was calculated with

$${c=\sum_{i} {\left( \frac{\left| \mathbf{p}_{\boldsymbol{i}} \right|+\left| \mathbf{q}_{\boldsymbol{i}} \right|}{2} \right)c}_{i}}/{\sum_{i} \left( \frac{\left| \mathbf{p}_{\boldsymbol{i}} \right|+\left| \mathbf{q}_{\boldsymbol{i}} \right|}{2} \right)}$$

If node *i* of a dendritic spine is connected to node *j* of its parental dendrite, the weight term was replaced with

$$\left( \left| \mathbf{p}_{\boldsymbol{i}} \right|-r_{j} \right)+\frac{\left| \mathbf{q}_{\boldsymbol{i}} \right|}{2}$$

where *r_j_* is the radius of node *j*.

Torsion *τ_ij_* at an edge between node *i* and node *j* was defined using the upstream vector **p***_i_*, downstream vector **q***_j_*, and edge vector **s***_ij_* (Supplementary Fig. S9d). Binormal vectors **b***_i_* at node *i* and **b***_j_* at node *j* were defined as

$$\mathbf{b}_{i}=\frac{\mathbf{p}_{i}\times\mathbf{s}_{ij}}{\left| \mathbf{p}_{i}\times\mathbf{s}_{ij} \right|}$$

$$\mathbf{b}_{j}=\frac{\mathbf{s}_{ij}\times\mathbf{q}_{j}}{\left| \mathbf{s}_{ij}\times\mathbf{q}_{j} \right|}$$

If the inner product of these binormal vectors was less than 0, vector **b***_j_* was multiplied by -1. Then torsion *τ_ij_* was calculated with

$$\tau_{ij}=\left\{ \begin{aligned} -\frac{\left| \mathbf{b}_{j}-\mathbf{b}_{i} \right|}{\left| \mathbf{s}_{ij} \right|}, \mathrm{if} \left( \frac{\mathbf{s}_{ij}}{\left| \mathbf{s}_{ij} \right|}-\frac{\mathbf{p}_{i}}{\left| \mathbf{p}_{i} \right|} \right)\cdot\left( \mathbf{b}_{j}-\mathbf{b}_{i} \right)>0 \\ \frac{\left| \mathbf{b}_{j}-\mathbf{b}_{i} \right|}{\left| \mathbf{s}_{ij} \right|}, \mathrm{if} \left( \frac{\mathbf{s}_{ij}}{\left| \mathbf{s}_{ij} \right|}-\frac{\mathbf{p}_{i}}{\left| \mathbf{p}_{i} \right|} \right)\cdot\left( \mathbf{b}_{j}-\mathbf{b}_{i} \right)\leq0 \end{aligned} \right.$$

The weighted-mean torsion *τ* of a neurite segment was calculated with:

$${\tau=\sum{\left| \mathbf{s}_{ij} \right|\tau}_{ij}}/{\sum\left| \mathbf{s}_{ij} \right|}$$

**Statistical tests**

The statistical tests of the geometric parameters and spine density were performed using the R software. Significance was defined as *p* < 0.05. Equality of medians of the neurite curvature and torsion were examined with the Kruskal-Wallis test. Two-sided Welch's t-tests were used to examine the equality of the mean neurite curvature and mean spine density between the schizophrenia and control cases.

**References of Supplementary Materials and Methods**

1. Suzuki Y *et al.* Construction and commissioning of a 248 m-long beamline with X-ray undulator light source. *AIP Conference Proceedings* 2004; **705**: 344–347.
2. Mizutani R *et al.* Microtomographic analysis of neuronal circuits of human brain. *Cereb Cortex* 2010; **20**: 1739–1748.
3. Kolluri N, Sun Z, Sampson AR, Lewis DA. Lamina-specific reductions in dendritic spine density in the prefrontal cortex of subjects with schizophrenia. *Am J Psychiatry* 2005; **162**: 1200–1202.
4. Xu C, Prince JL. Snakes, shapes, and gradient vector flow. *IEEE Trans Image Process* 1998; **7**: 359–369.
5. Al-Kofahi KA *et al.* Rapid automated three-dimensional tracing of neurons from confocal image stacks. *IEEE Trans Inf Technol Biomed* 2002; **6**: 171–187.
6. Kleywegt GJ, Jones TA. Model building and refinement practice. *Methods Enzymol* 1997; **277**: 208–230.
7. Mizutani R, Saiga R, Takeuchi A, Uesugi K, Suzuki Y. Three-dimensional network of Drosophila brain hemisphere. *J Struct Biol* 2013; **184**: 271–279.
8. Luzzati V. Traitement statistique des erreurs dans la determination des structures cristallines. *Acta Cryst* 1952; **5**: 802–810.
9. Read RJ. Improved Fourier coefficients for maps using phases from partial structures with errors. *Acta Cryst* 1986; **A42**: 140–149.
10. Nimchinsky EA, Vogt BA, Morrison JH, Hof PR. Spindle neurons of the human anterior cingulate cortex. *J Comp Neurol* 1995; **355**: 27–37.

**a b**


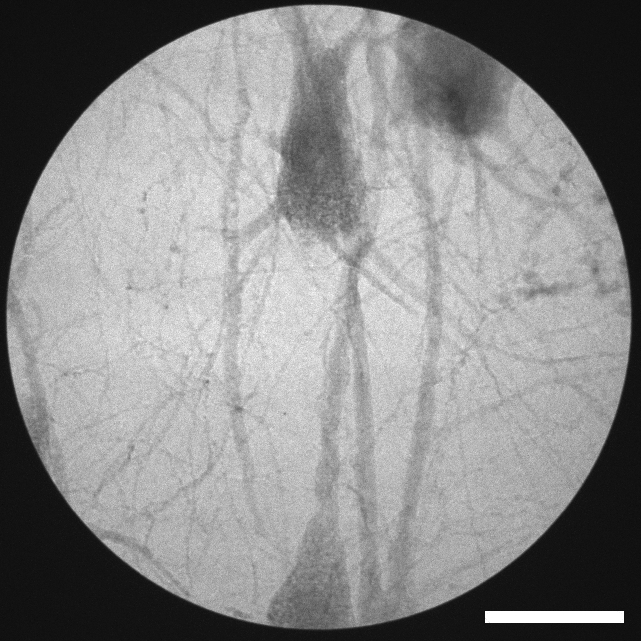

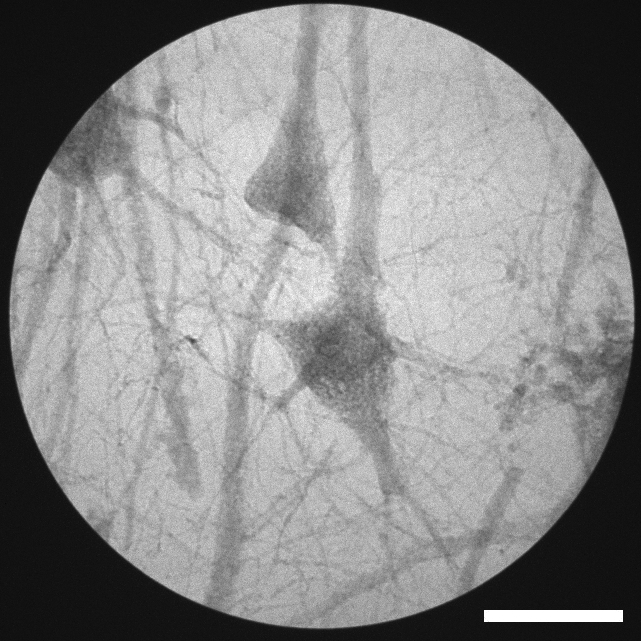


**c d**


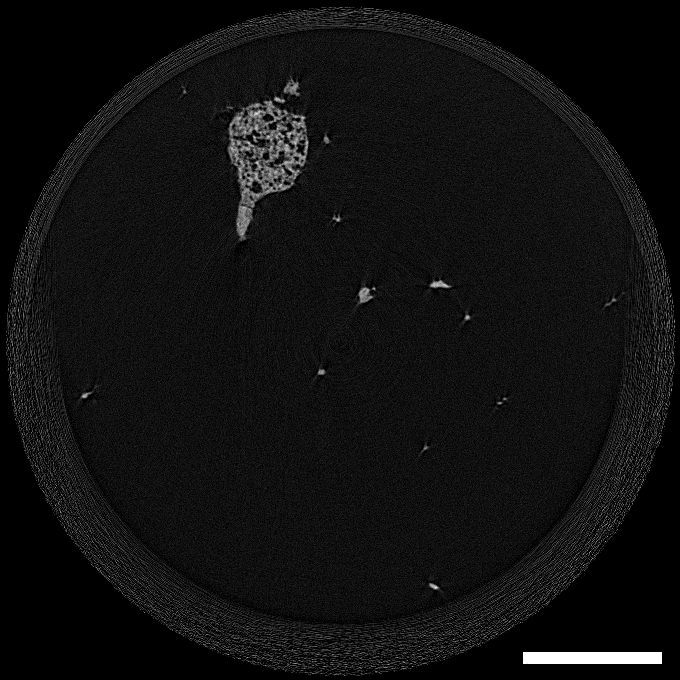

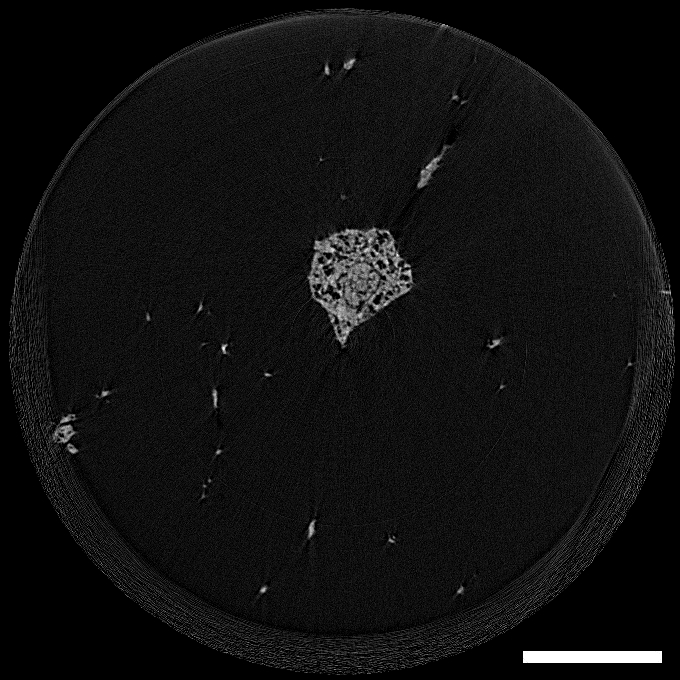


**Supplementary Figure S1.** Absorption-contrast raw images (**a**,**b**) and reconstructed slices (**c**,**d**) of dataset N2C of the control N2 case. The three-dimensional rendering of this structure is shown in Figure 1a. Scale bars: 20 μm. (**a**) Raw image of the interneuron in Figure 1a. Pixel values of 0 to 1800 are linearly gray-scaled. (**b**) Raw image of the pyramidal neurons seen in Figure 1a. Pixel values of 0 to 1800 are linearly gray-scaled. (**c**) Tomographic slice at the soma of the interneuron in panel **a**. Voxel values of -100 to 1700 are linearly gray-scaled. (**d**) Tomographic slice at the soma of the lower pyramidal neuron in panel **b**. Voxel values of -100 to 1700 are linearly gray-scaled.

**a b**


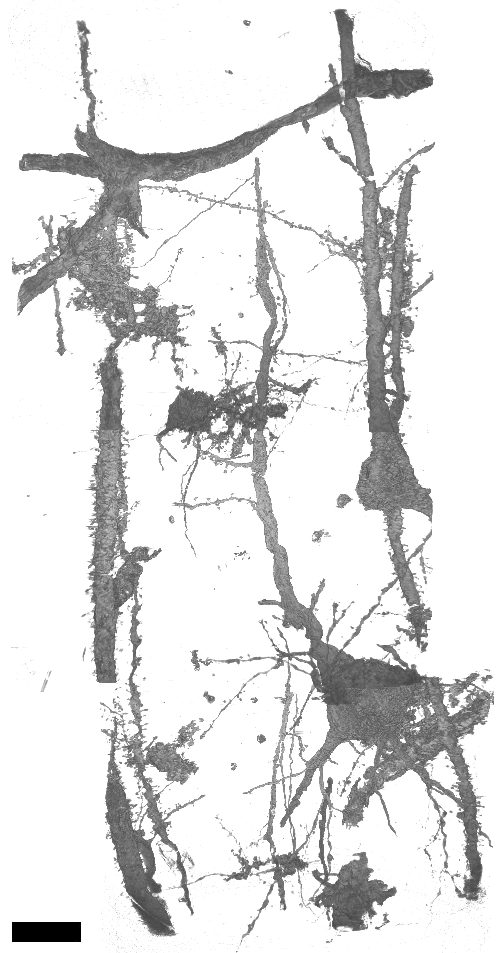

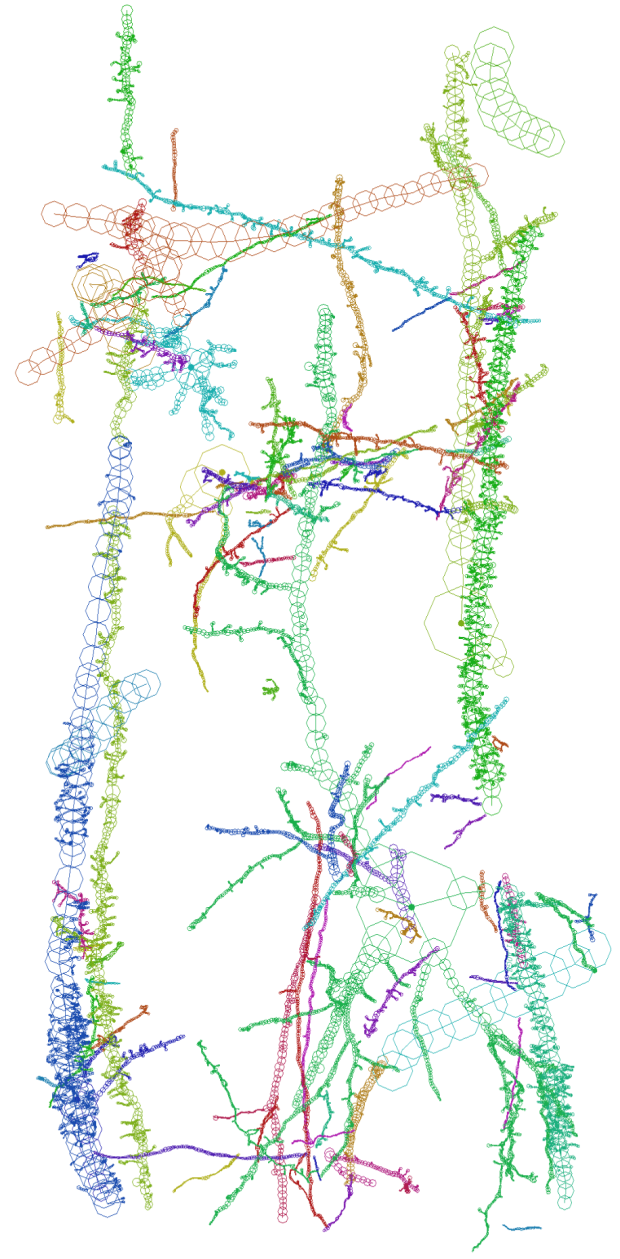


**Supplementary Figure S2.** Rendering of three-dimensional images of cerebral tissues and Cartesian coordinate models of tissue structures. Renderings and models are viewed from nearly the same direction. The pial surface is toward the top. Images were rendered with the scatter HQ algorithm in the VG Studio software. Models were drawn with the MCTrace software. Model constituents are color-coded. Nodes composing each constituent are indicated with circles. Dots indicate somata nodes. Scale bars: 20 μm. (**a**) Rendering of image dataset S1B of the schizophrenia S1 case. Voxel values of 200–1200 were rendered in gray scale. (**b**) Cartesian coordinate model of S1B.

**c d**


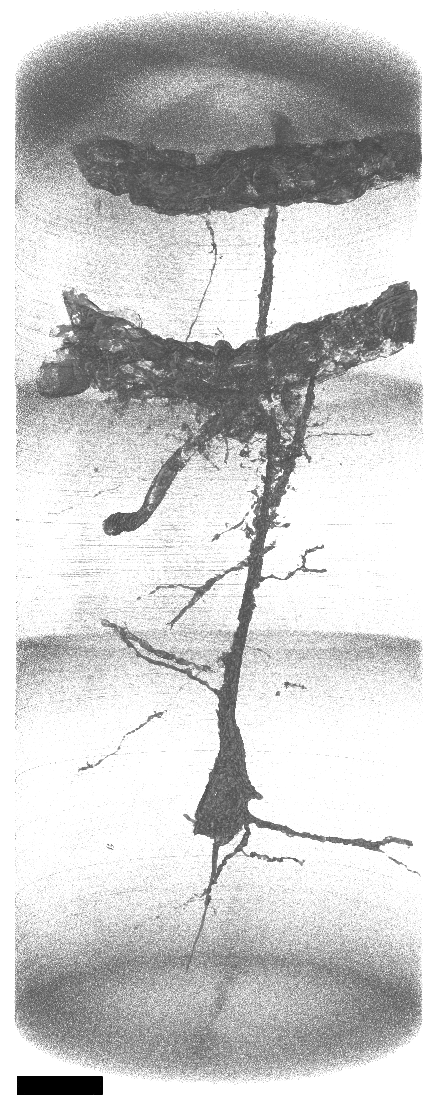

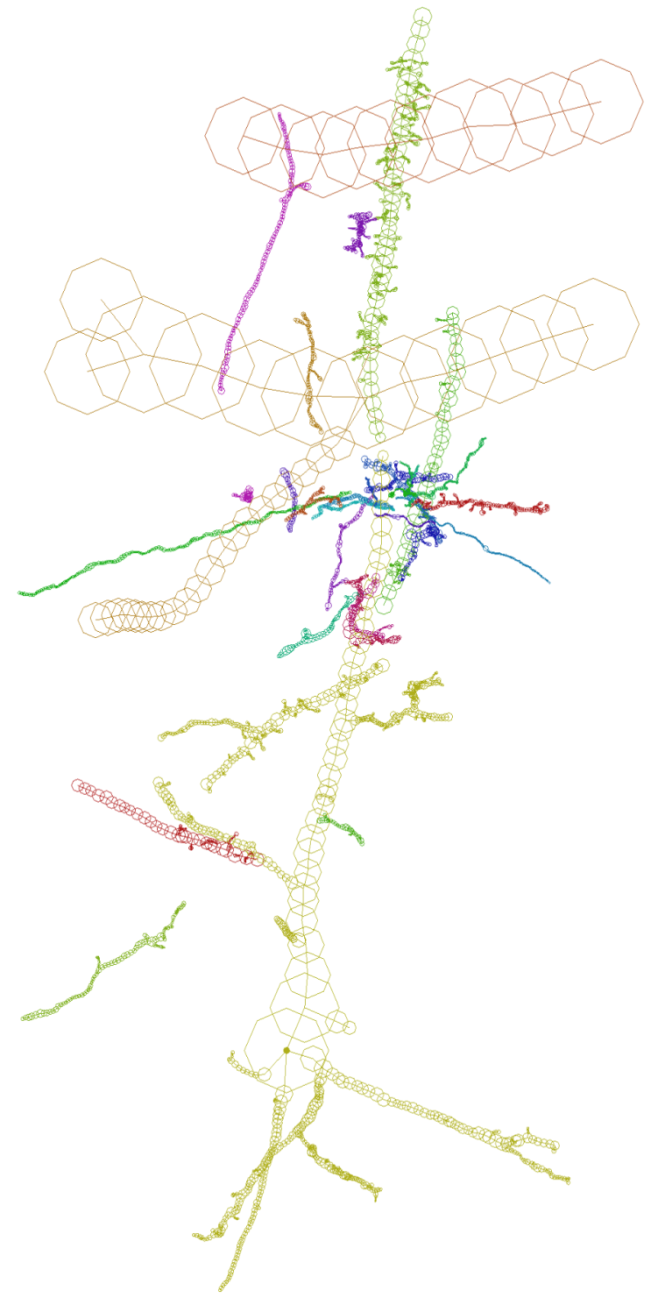


**Supplementary Figure S2 (cont'd).** Rendering of three-dimensional images of cerebral tissues and Cartesian coordinate models of tissue structures. (**c**) Rendering of schizophrenia dataset S2E. Voxel values of 500–1600 were rendered in gray scale. (**d**) Model of S2E.

**e f**


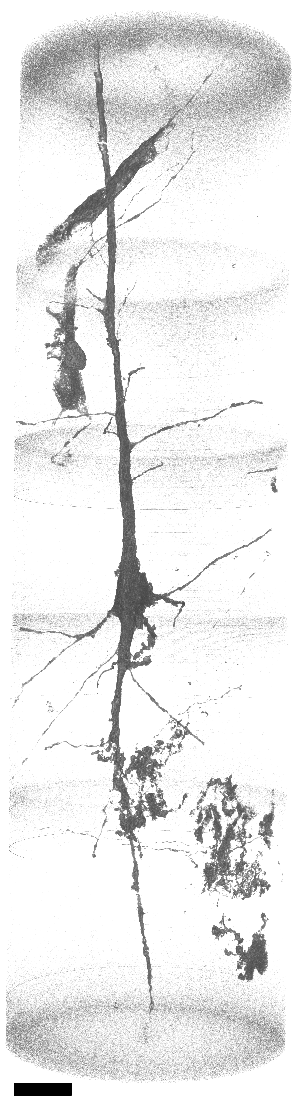

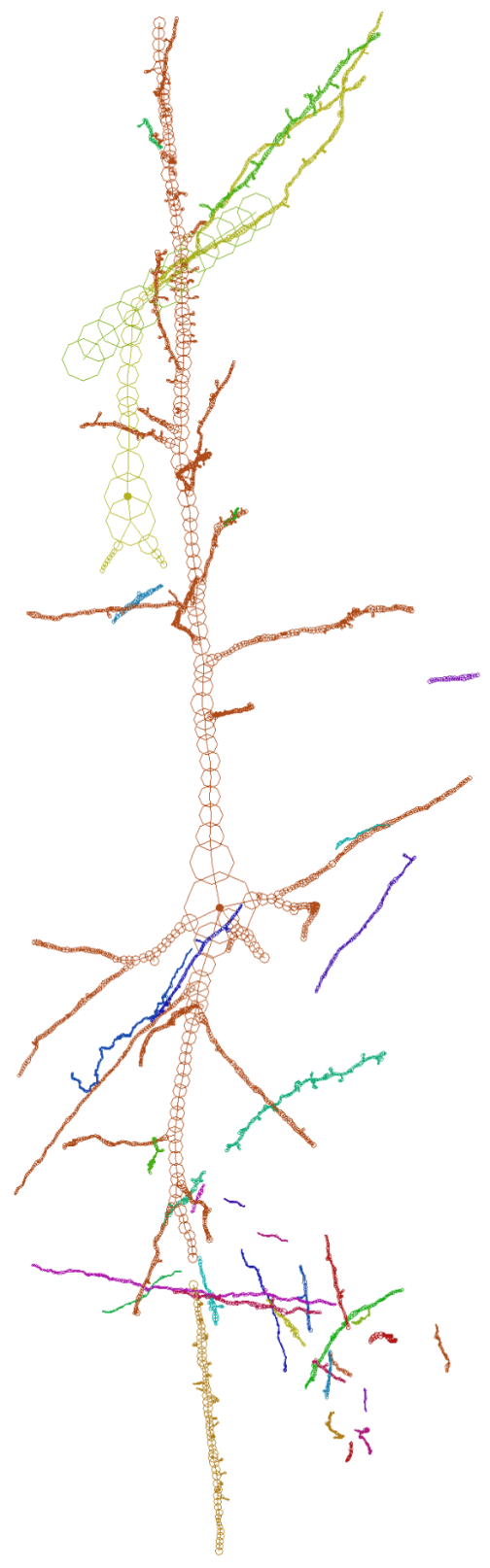


**Supplementary Figure S2 (cont'd).** Rendering of three-dimensional images of cerebral tissues and Cartesian coordinate models of tissue structures. (**e**) Rendering of schizophrenia dataset S3G. Voxel values of 500–1600 were rendered in gray scale. (**f**) Model of S3G.

**g h**


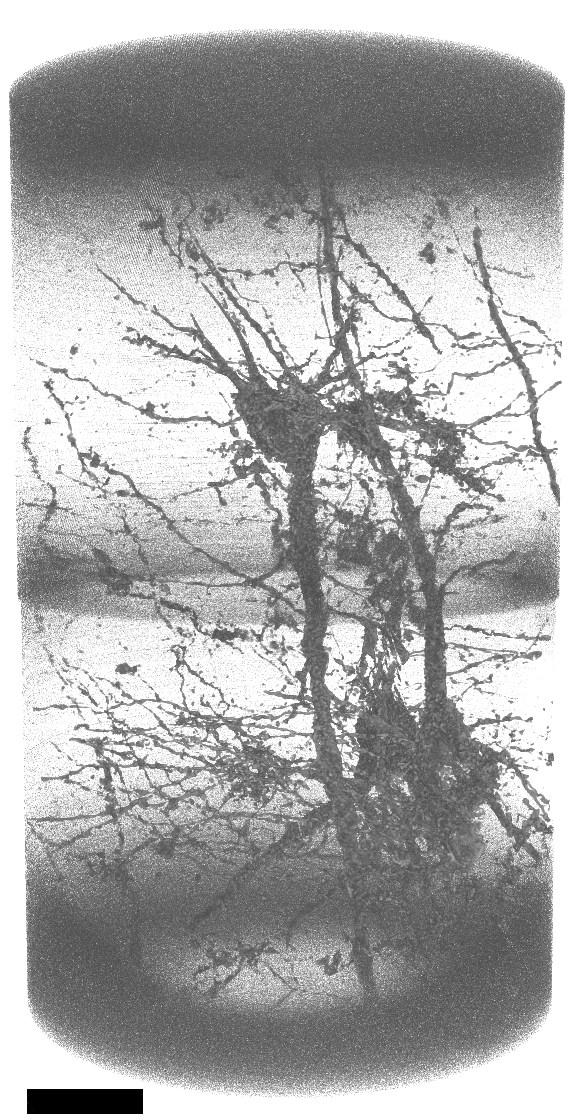

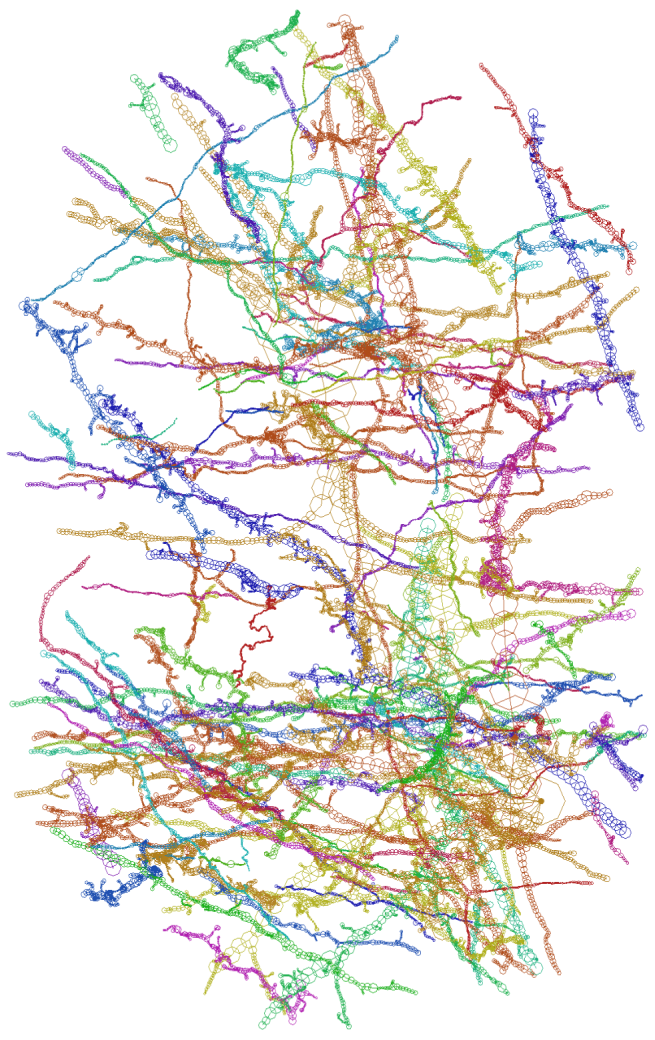


**Supplementary Figure S2 (cont'd).** Rendering of three-dimensional images of cerebral tissues and Cartesian coordinate models of tissue structures. (**g**) Rendering of schizophrenia dataset S4C. Voxel values of 500–1600 were rendered in gray scale. (**h**) Model of S4C.

**i j**


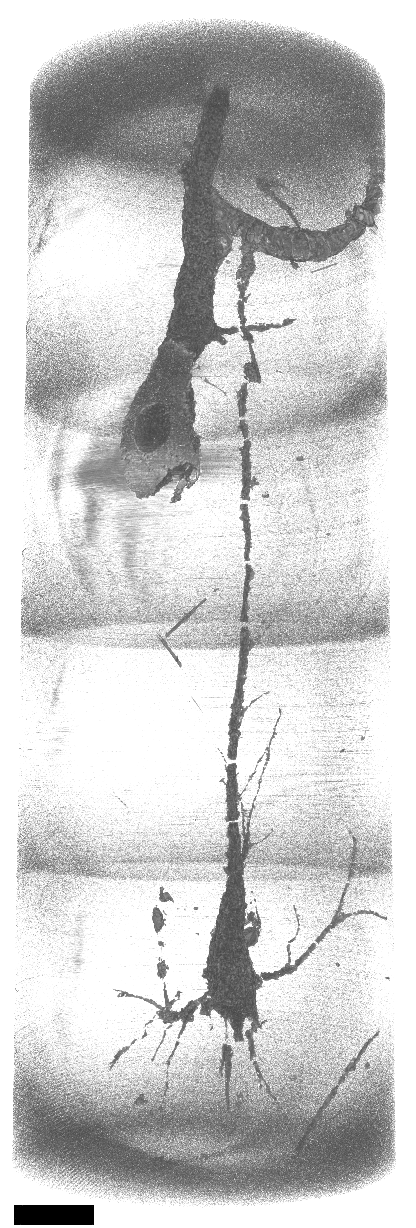

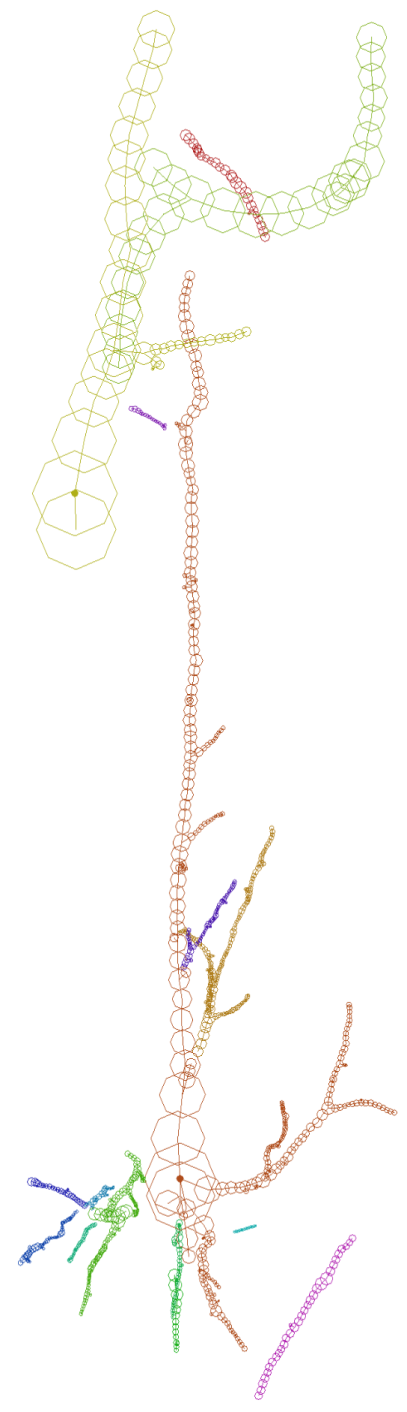


**Supplementary Figure S2 (cont'd).** Rendering of three-dimensional images of cerebral tissues and Cartesian coordinate models of tissue structures. (**i**) Rendering of control dataset N1E. Voxel values of 500–1600 were rendered in gray scale. (**j**) Model of N1E.

**k l**


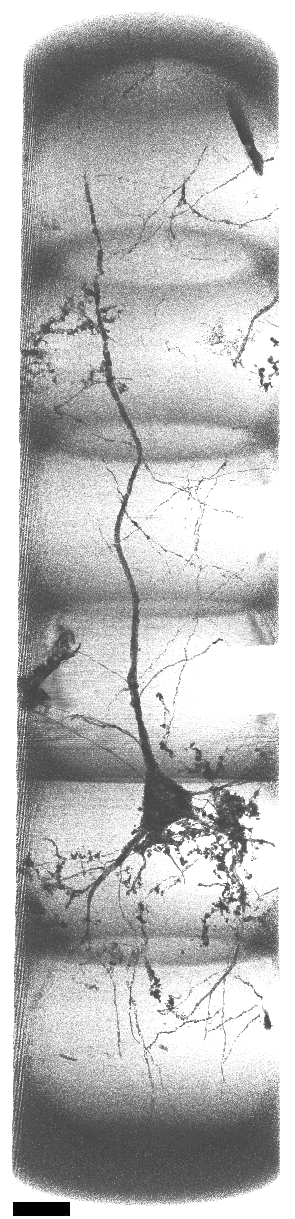

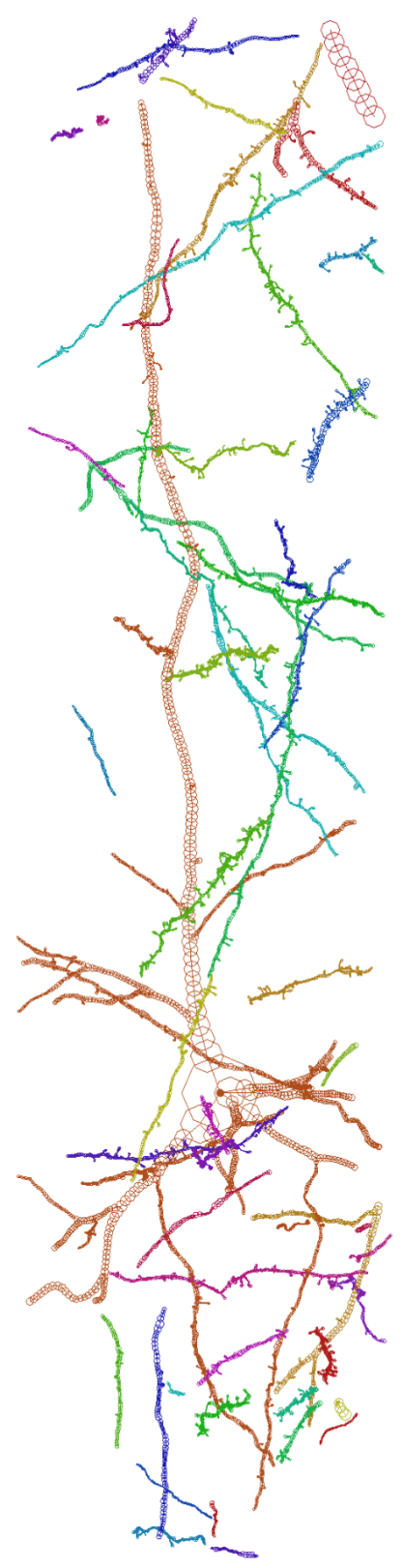


**Supplementary Figure S2 (cont'd).** Rendering of three-dimensional images of cerebral tissues and Cartesian coordinate models of tissue structures. (**k**) Rendering of control dataset N3D. Voxel values of 600–3200 were rendered in gray scale. (**l**) Model of N3D.

**m n**


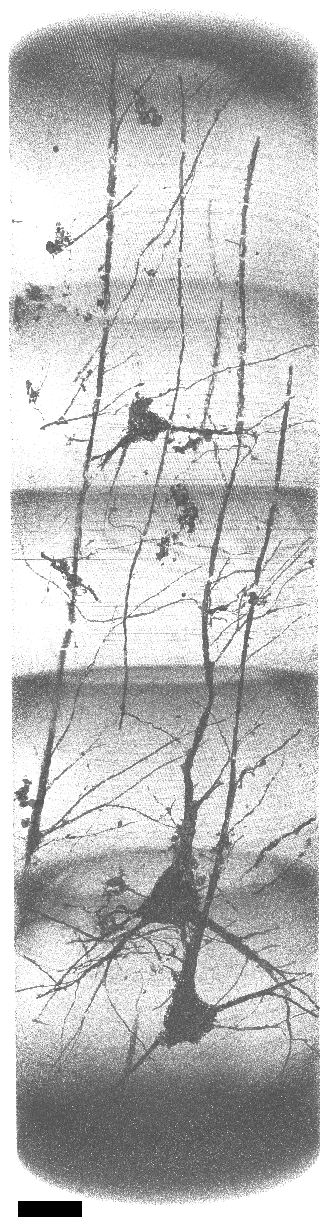

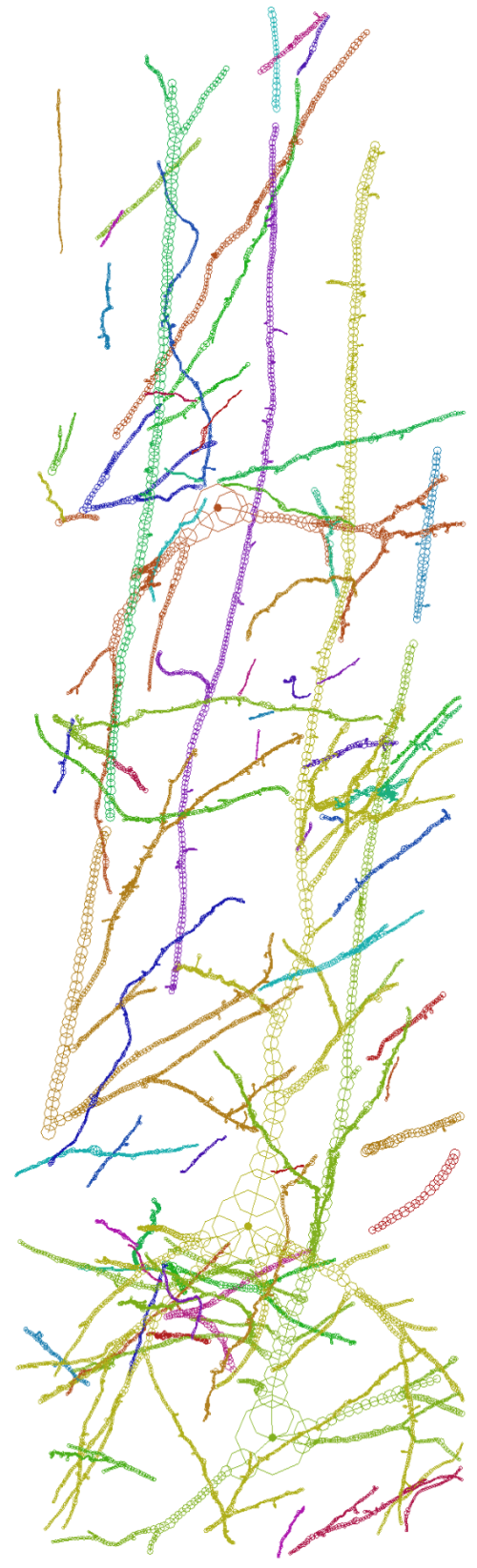


**Supplementary Figure S2 (cont'd).** Rendering of three-dimensional images of cerebral tissues and Cartesian coordinate models of tissue structures. (**m**) Rendering of control dataset N4A. Voxel values of 500–1600 were rendered in gray scale. (**n**) Model of N4A.


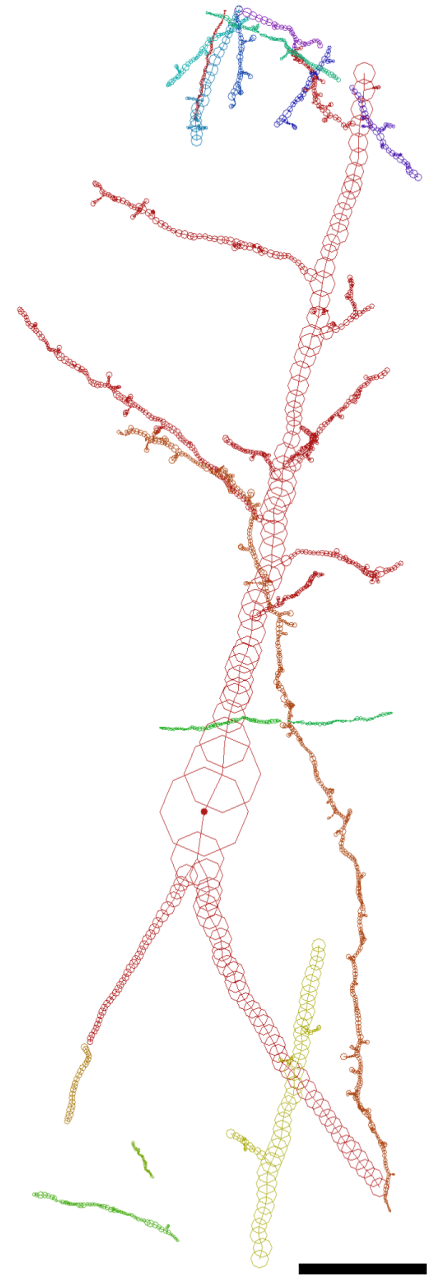

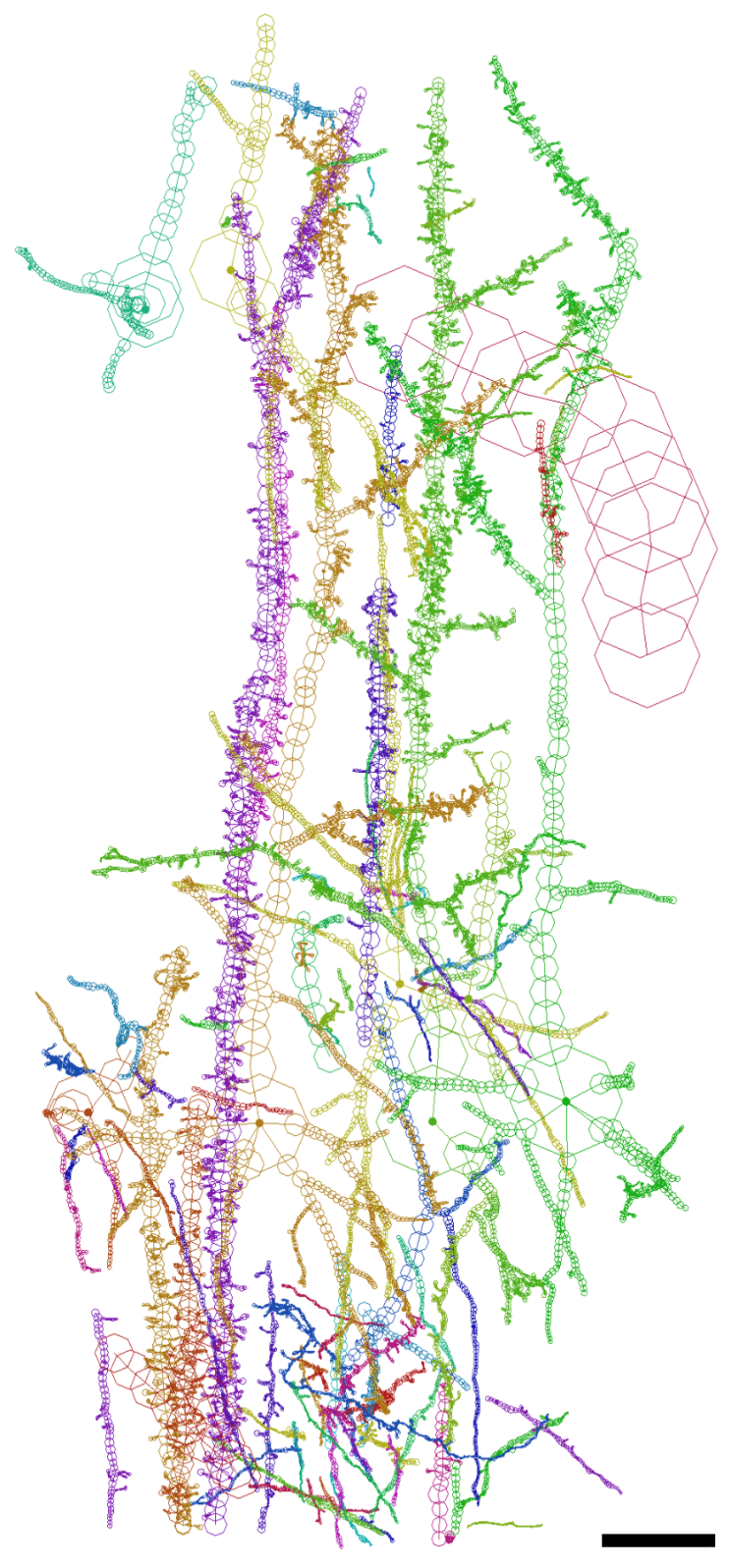


(**a**) S1A structure. (**b**) S1C structure.

**Supplementary Figure S3.** Cartesian coordinate models of schizophrenia tissue structures. The pial surface is toward the top. The models were drawn with the MCTrace software. Constituents of the models are color-coded. Nodes composing each constituent are indicated with circles. Dots indicate somata nodes. Scale bars: 20 μm.


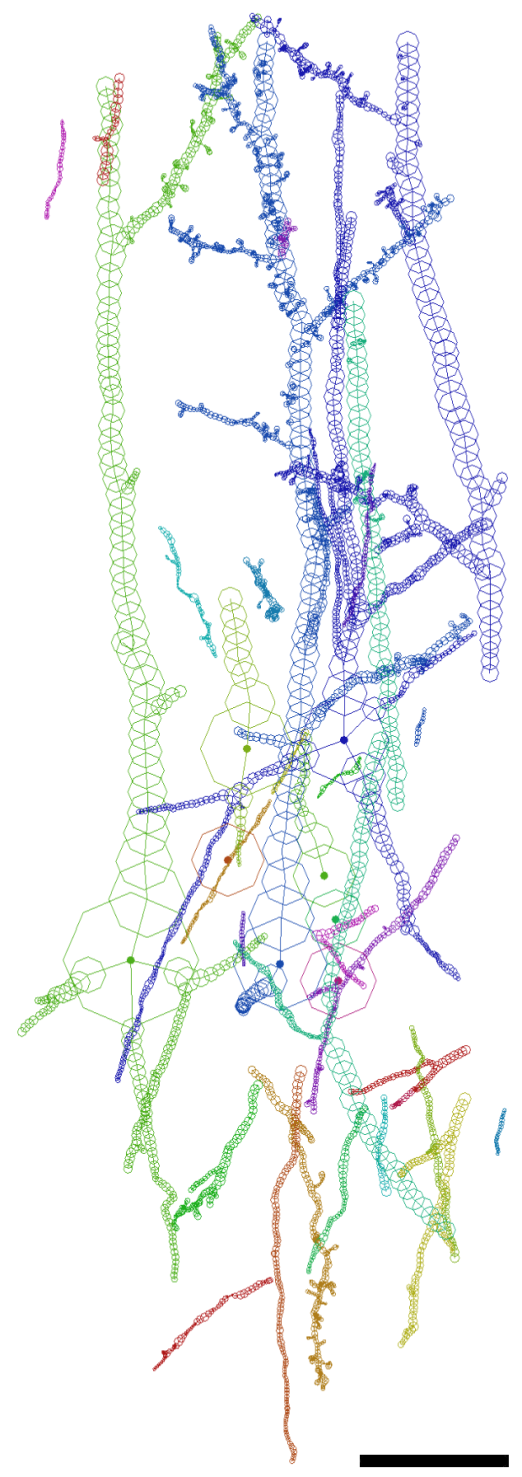

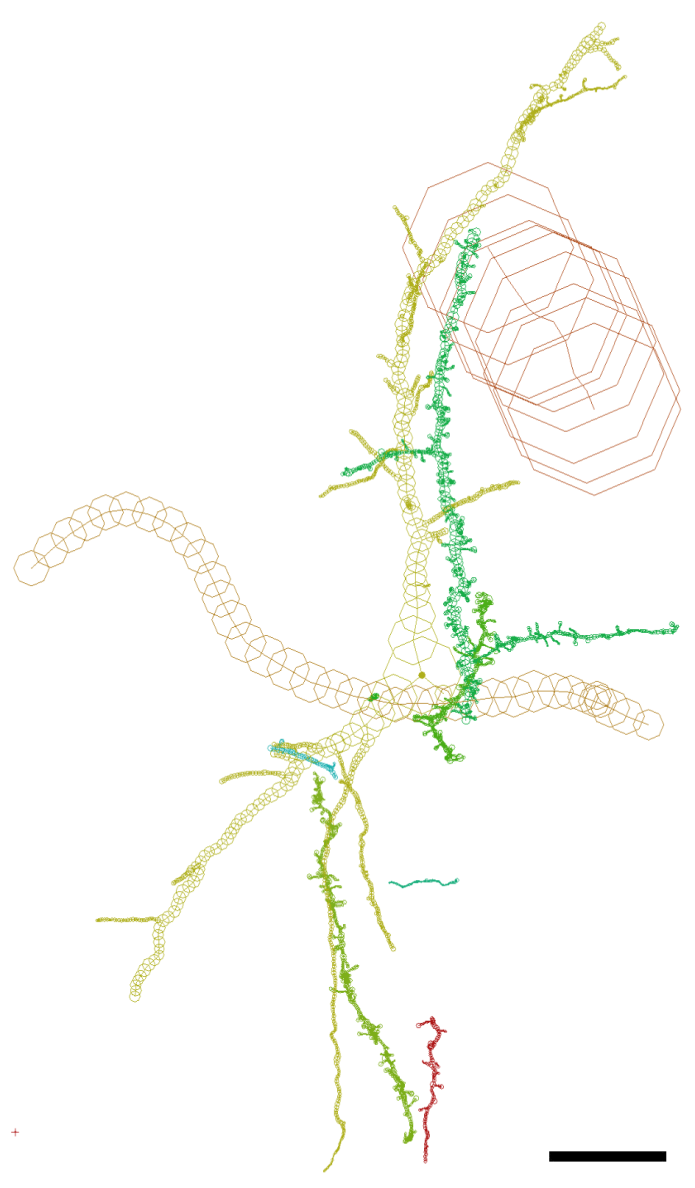


(**c**) S1D structure. (**d**) S1E structure.

**Supplementary Figure S3 (cont'd).** Cartesian coordinate models of schizophrenia tissue structures. The pial surface is toward the top. The models were drawn with the MCTrace software. Constituents of the models are color-coded. Nodes composing each constituent are indicated with circles. Dots indicate somata nodes. Scale bars: 20 μm.


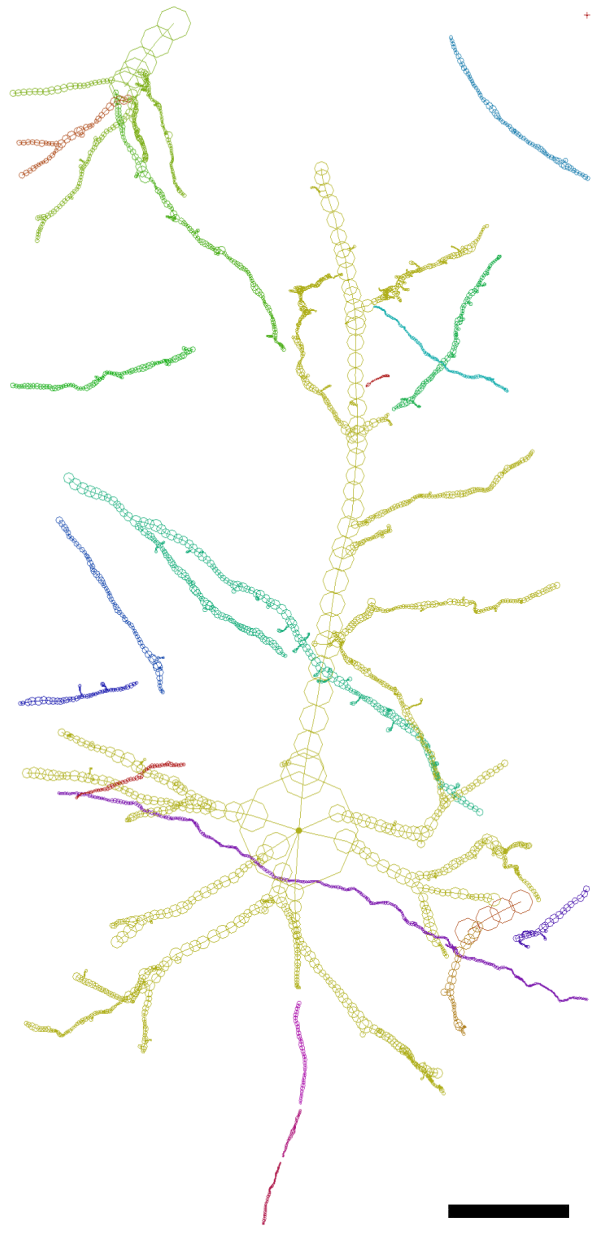

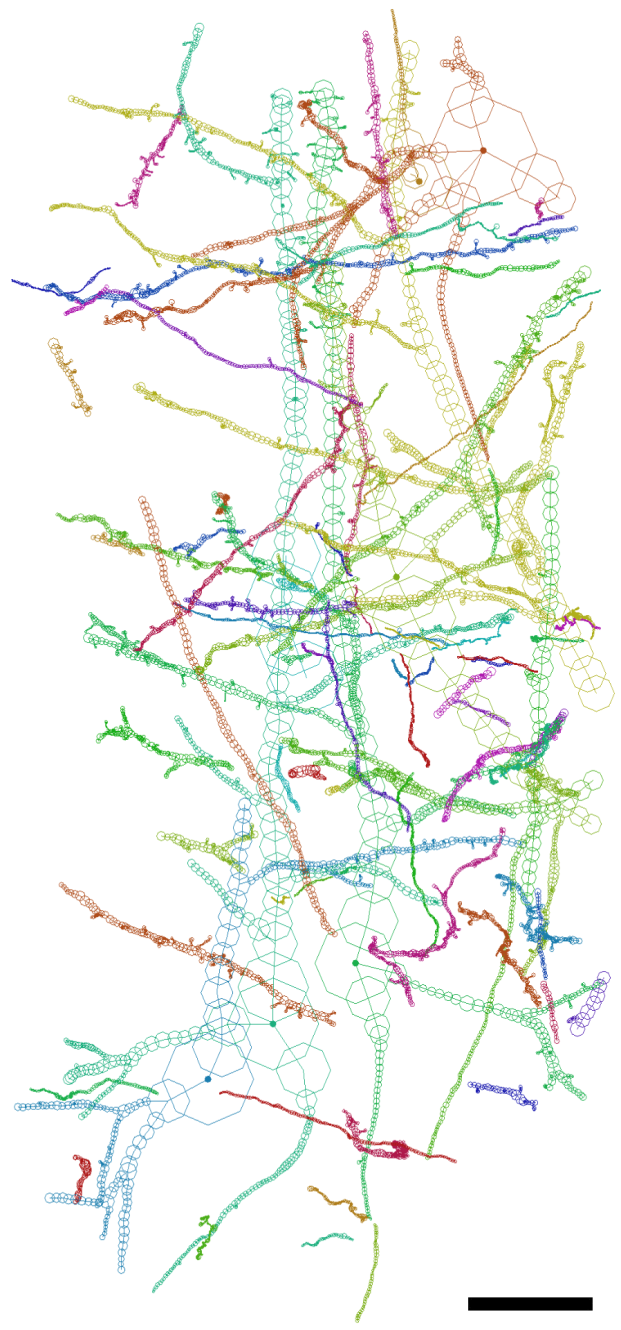


(**e**) S2A structure. (**f**) S2B structure.

**Supplementary Figure S3 (cont'd).** Cartesian coordinate models of schizophrenia tissue structures. The pial surface is toward the top. The models were drawn with the MCTrace software. Constituents of the models are color-coded. Nodes composing each constituent are indicated with circles. Dots indicate somata nodes. Scale bars: 20 μm.


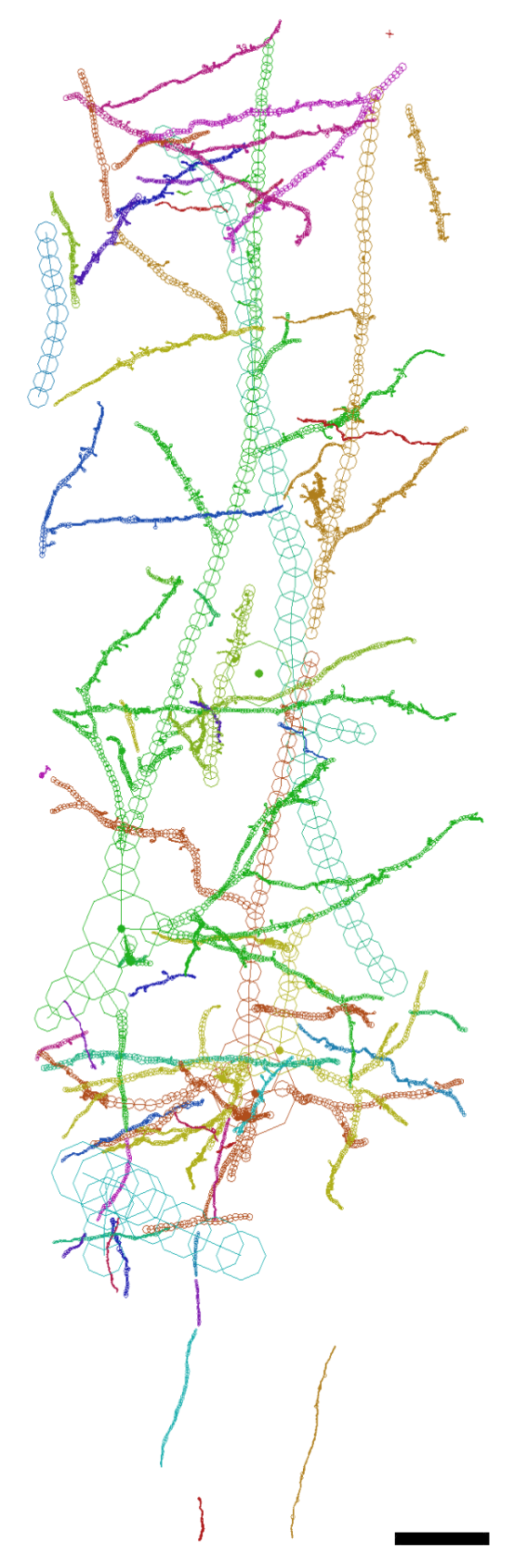

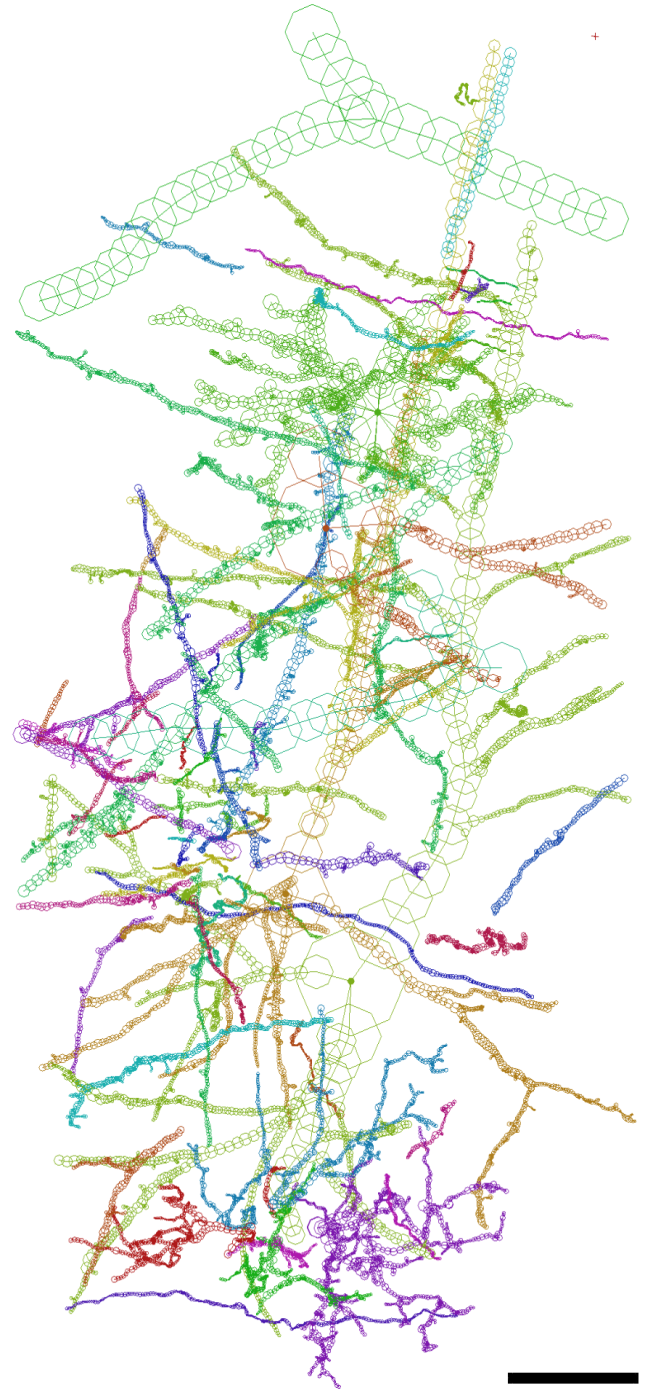


(**g**) S2C structure. (**h**) S2D structure.

**Supplementary Figure S3 (cont'd).** Cartesian coordinate models of schizophrenia tissue structures. The pial surface is toward the top. The models were drawn with the MCTrace software. Constituents of the models are color-coded. Nodes composing each constituent are indicated with circles. Dots indicate somata nodes. Scale bars: 20 μm.


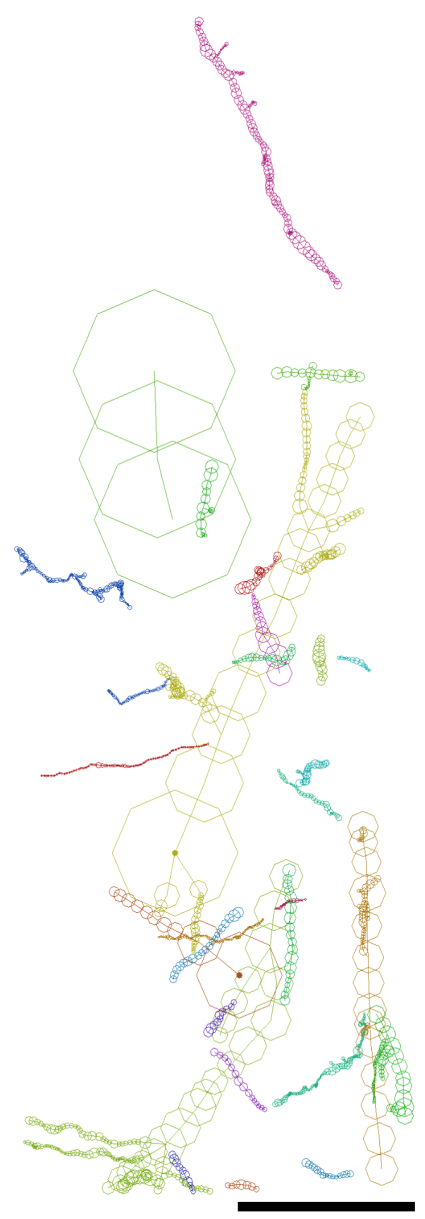

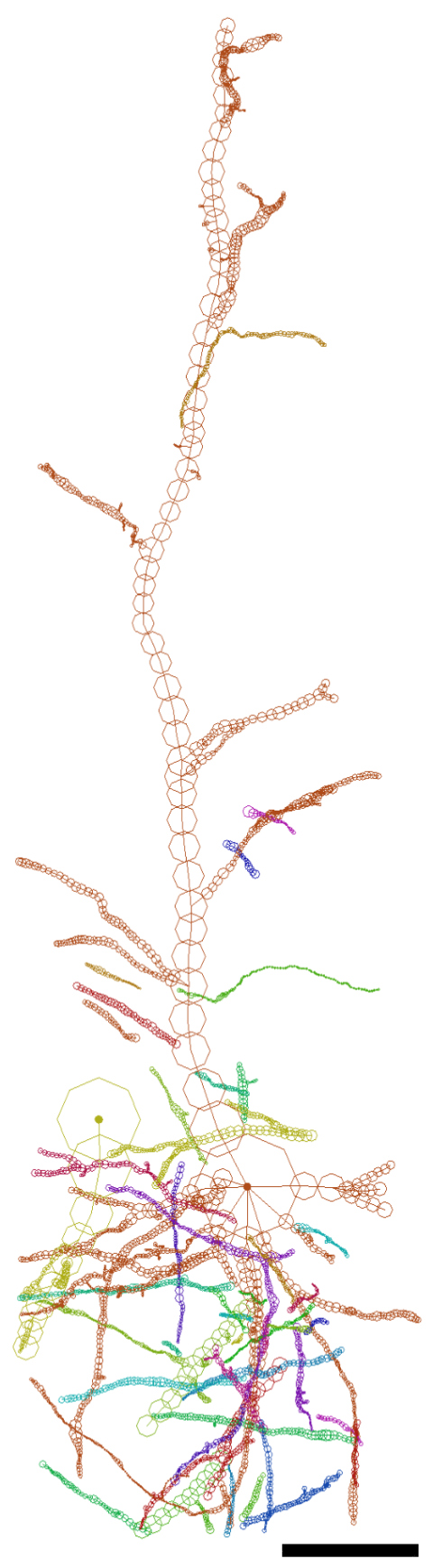


(**i**) S2F structure. (**j**) S2G structure.

**Supplementary Figure S3 (cont'd).** Cartesian coordinate models of schizophrenia tissue structures. The pial surface is toward the top. The models were drawn with the MCTrace software. Constituents of the models are color-coded. Nodes composing each constituent are indicated with circles. Dots indicate somata nodes. Scale bars: 20 μm.


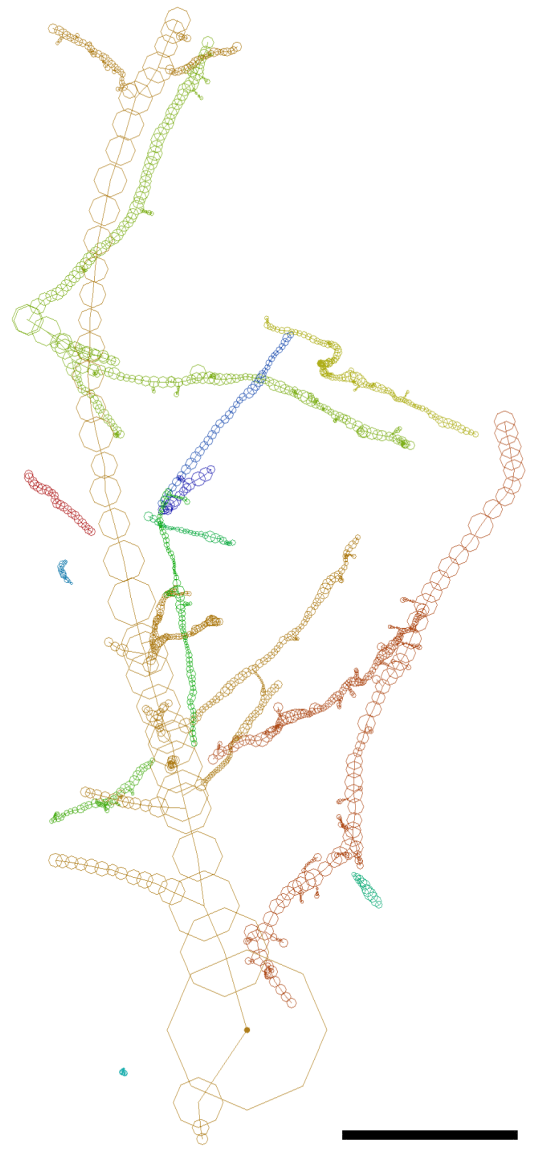

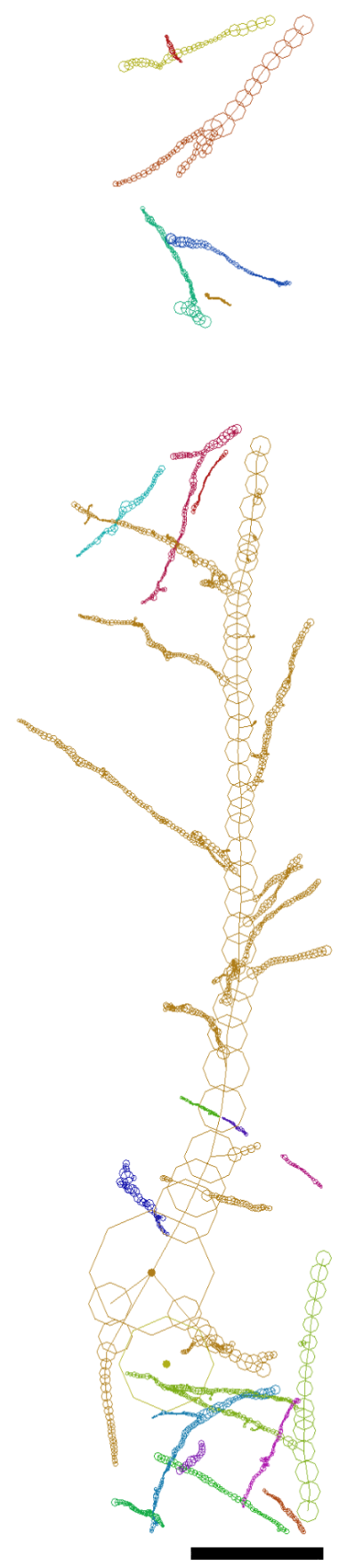


(**k**) S2H structure. (**l**) S2I structure.

**Supplementary Figure S3 (cont'd).** Cartesian coordinate models of schizophrenia tissue structures. The pial surface is toward the top. The models were drawn with the MCTrace software. Constituents of the models are color-coded. Nodes composing each constituent are indicated with circles. Dots indicate somata nodes. Scale bars: 20 μm.


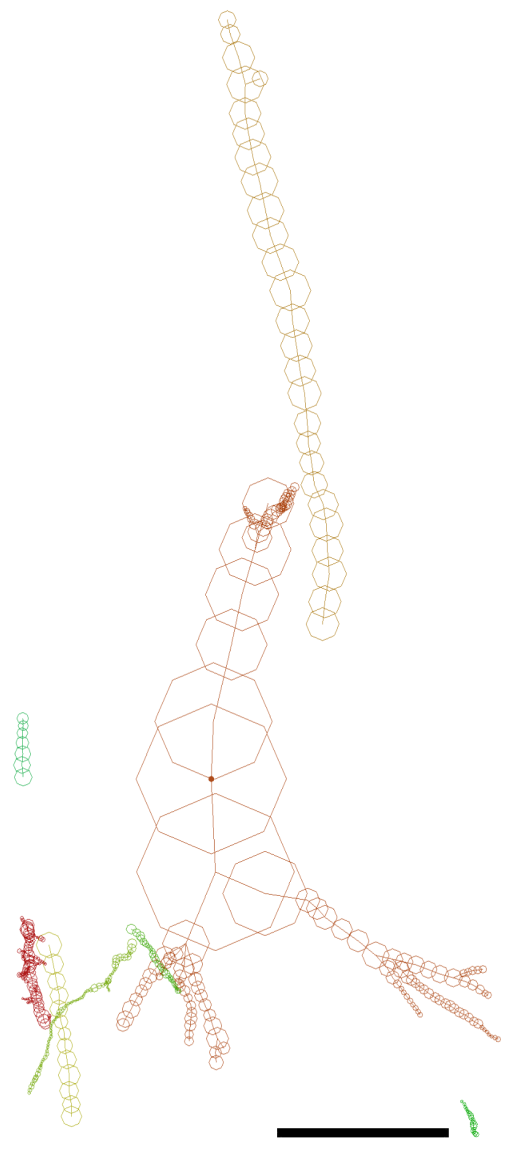

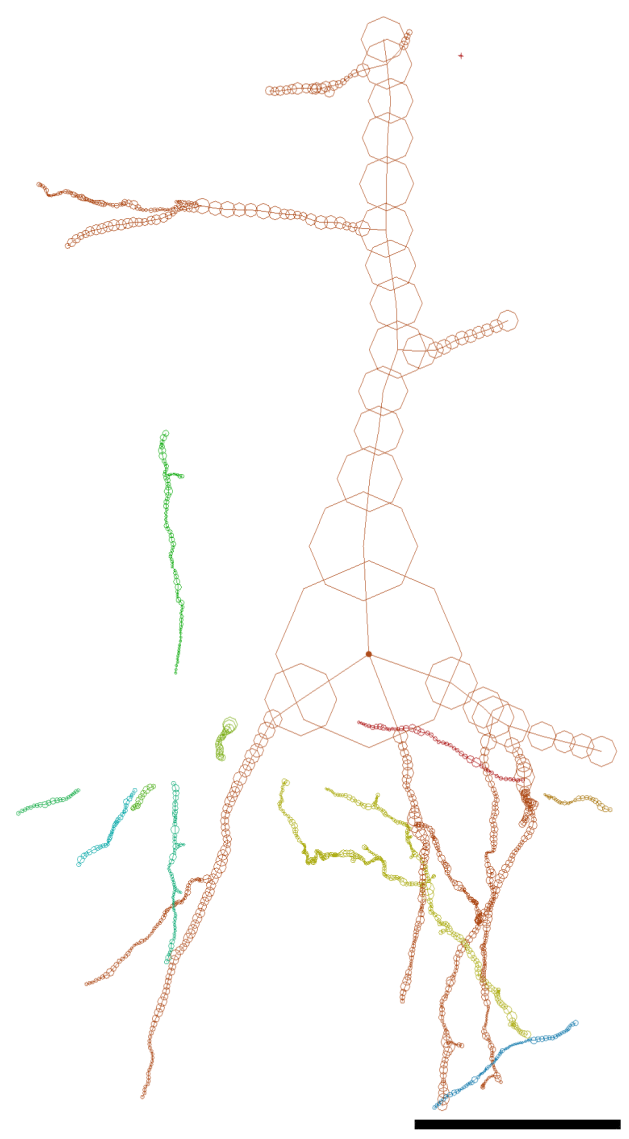


(**m**) S2J structure. (**n**) S3A structure.

**Supplementary Figure S3 (cont'd).** Cartesian coordinate models of schizophrenia tissue structures. The pial surface is toward the top. The models were drawn with the MCTrace software. Constituents of the models are color-coded. Nodes composing each constituent are indicated with circles. Dots indicate somata nodes. Scale bars: 20 μm.


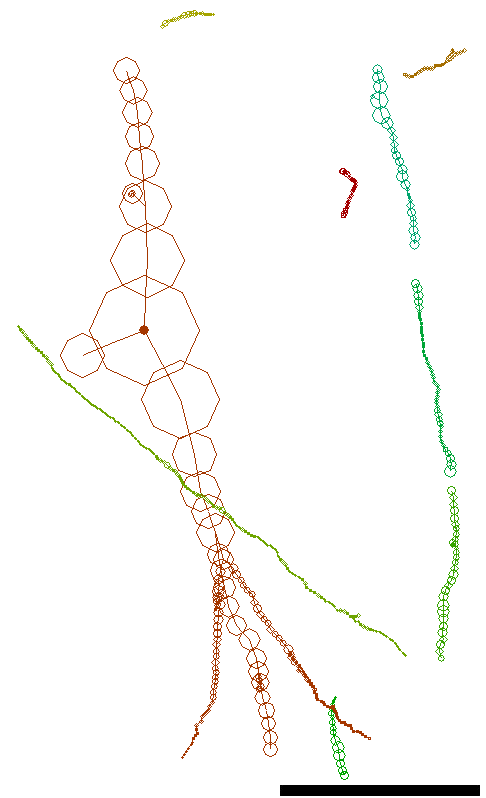

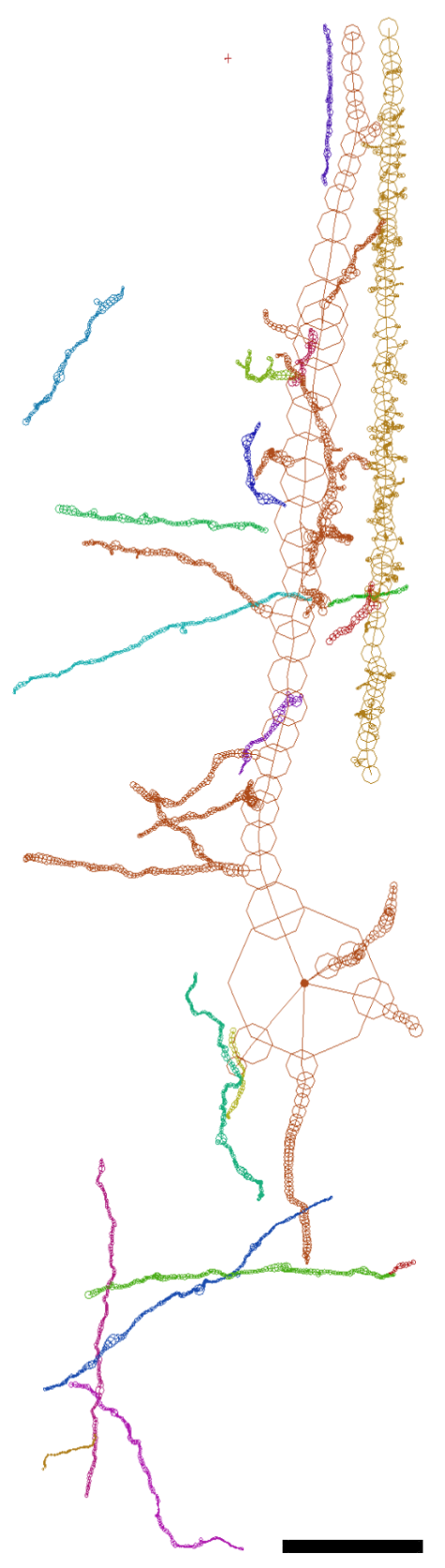


(**o**) S3B structure. (**p**) S3C structure.

**Supplementary Figure S3 (cont'd).** Cartesian coordinate models of schizophrenia tissue structures. The pial surface is toward the top. The models were drawn with the MCTrace software. Constituents of the models are color-coded. Nodes composing each constituent are indicated with circles. Dots indicate somata nodes. Scale bars: 20 μm.


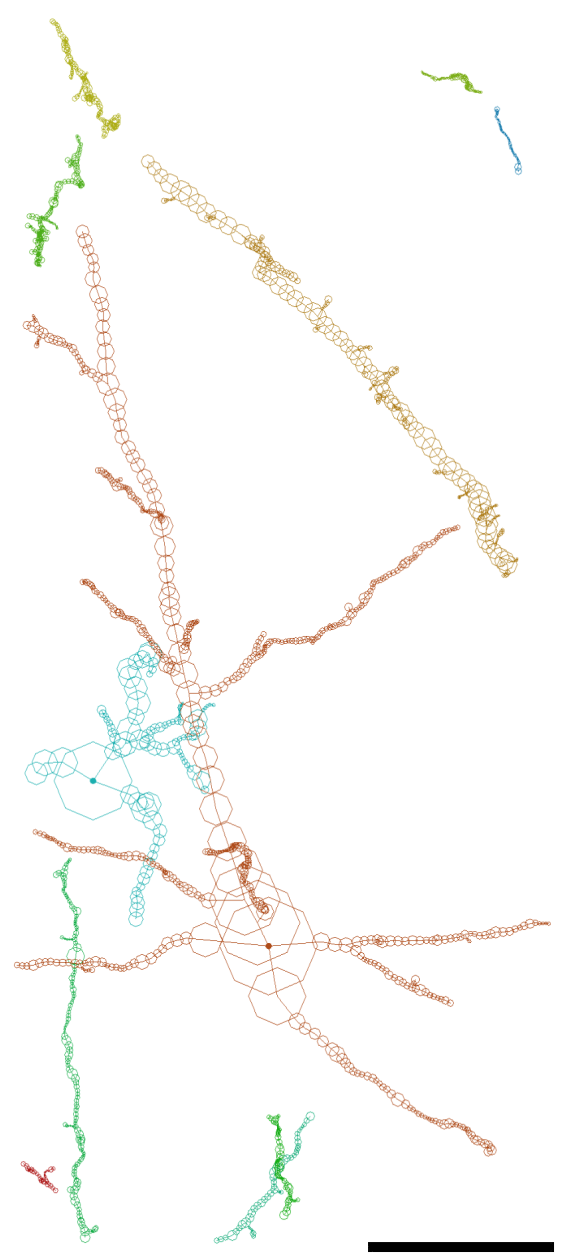

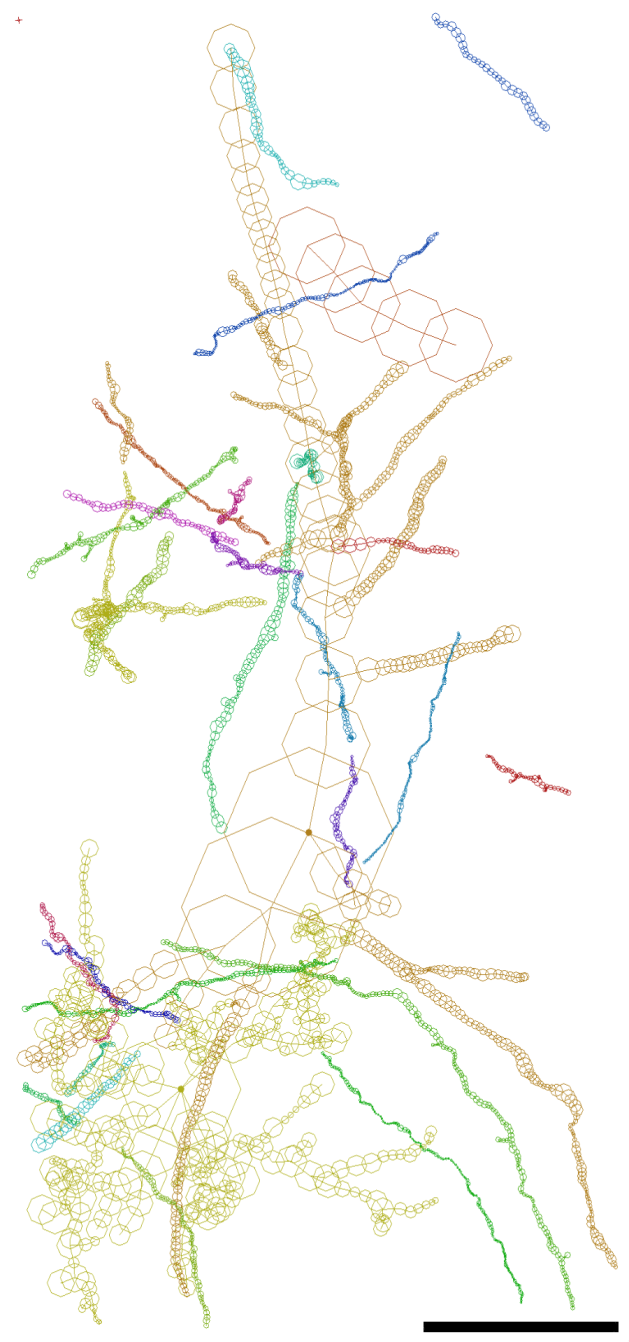


(**q**) S3D structure. (**r**) S3E structure.

**Supplementary Figure S3 (cont'd).** Cartesian coordinate models of schizophrenia tissue structures. The pial surface is toward the top. The models were drawn with the MCTrace software. Constituents of the models are color-coded. Nodes composing each constituent are indicated with circles. Dots indicate somata nodes. Scale bars: 20 μm.


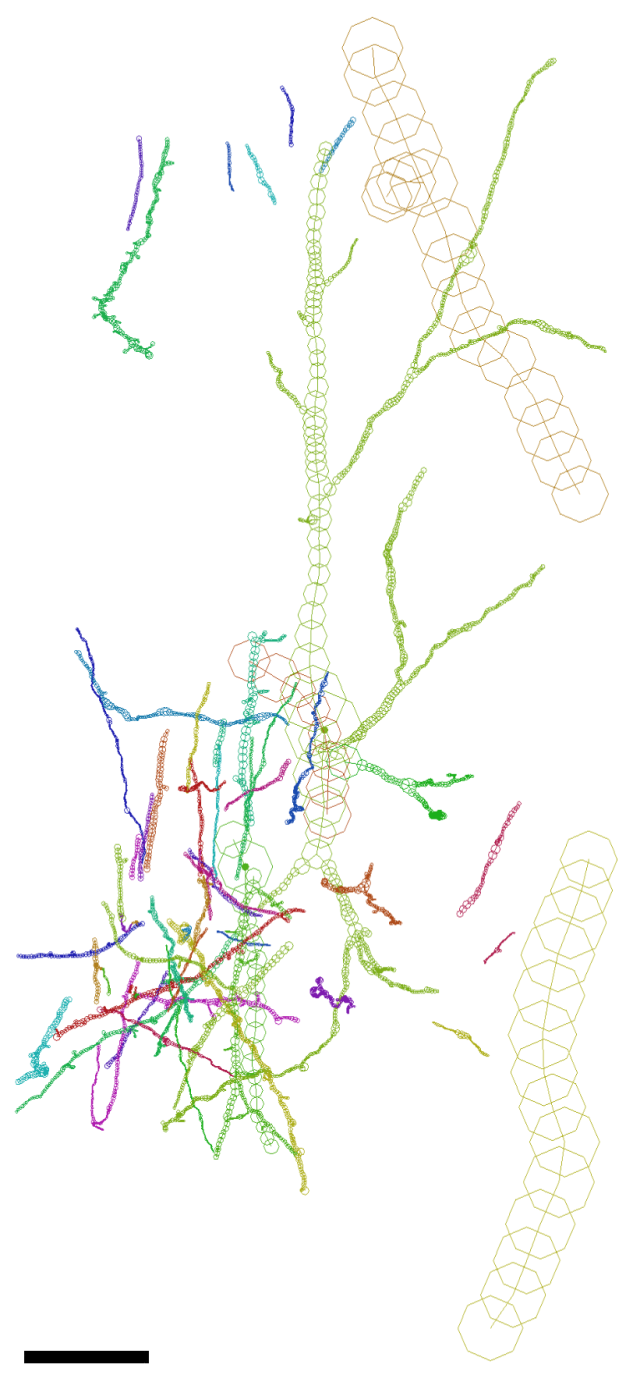

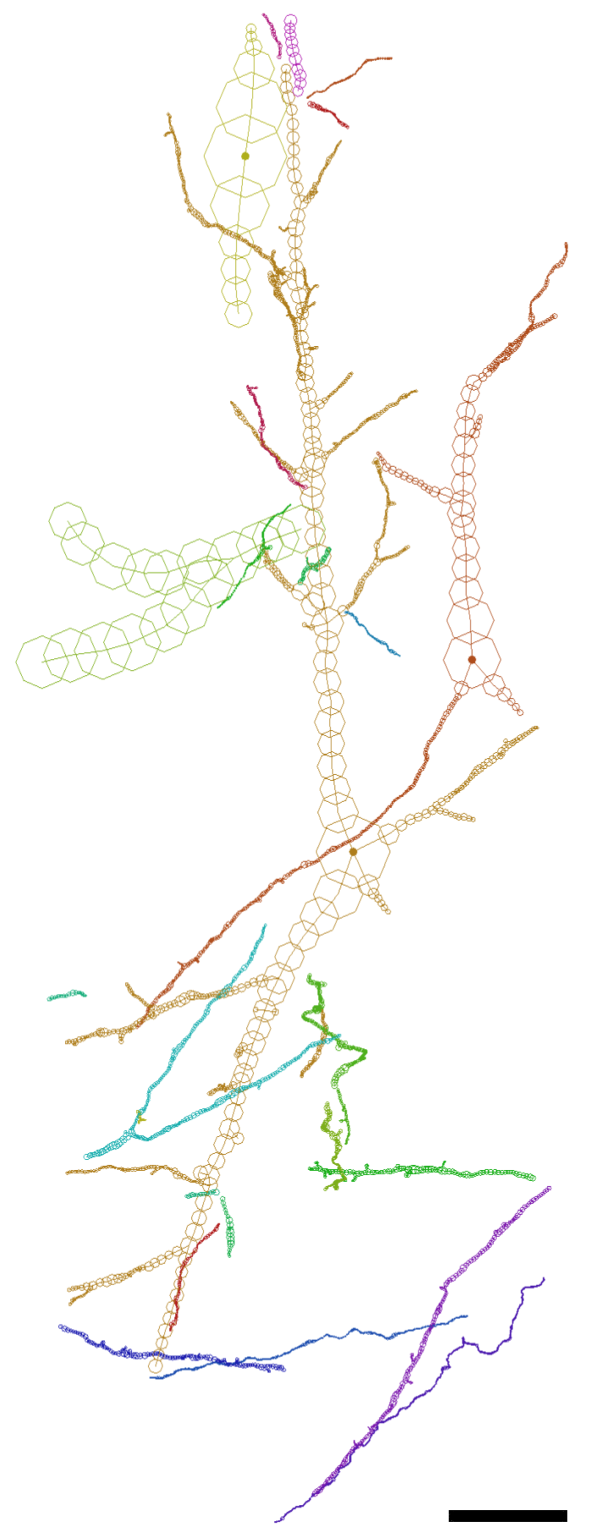


(**s**) S3F structure. (**t**) S3H structure.

**Supplementary Figure S3 (cont'd).** Cartesian coordinate models of schizophrenia tissue structures. The pial surface is toward the top. The models were drawn with the MCTrace software. Constituents of the models are color-coded. Nodes composing each constituent are indicated with circles. Dots indicate somata nodes. Scale bars: 20 μm.


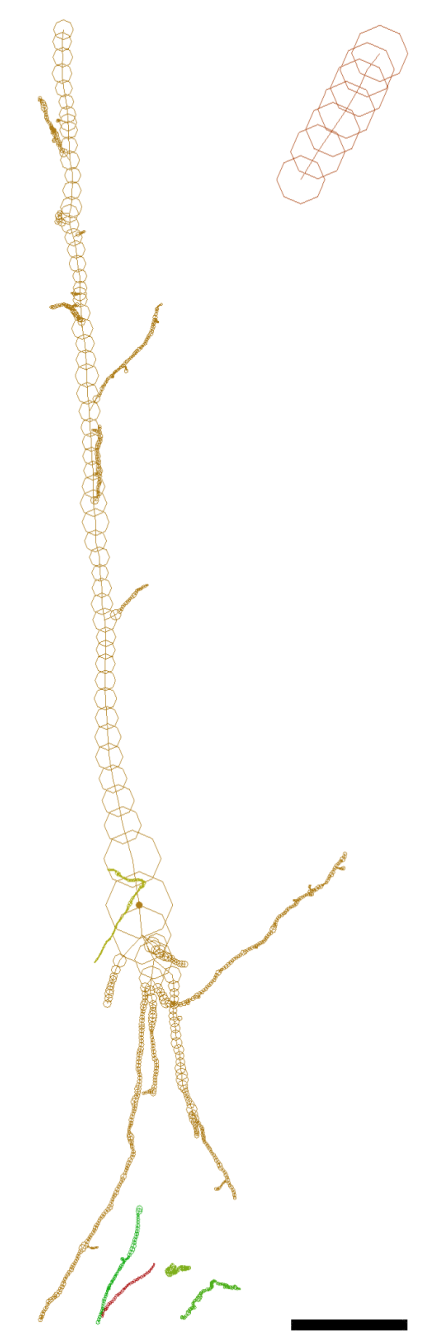

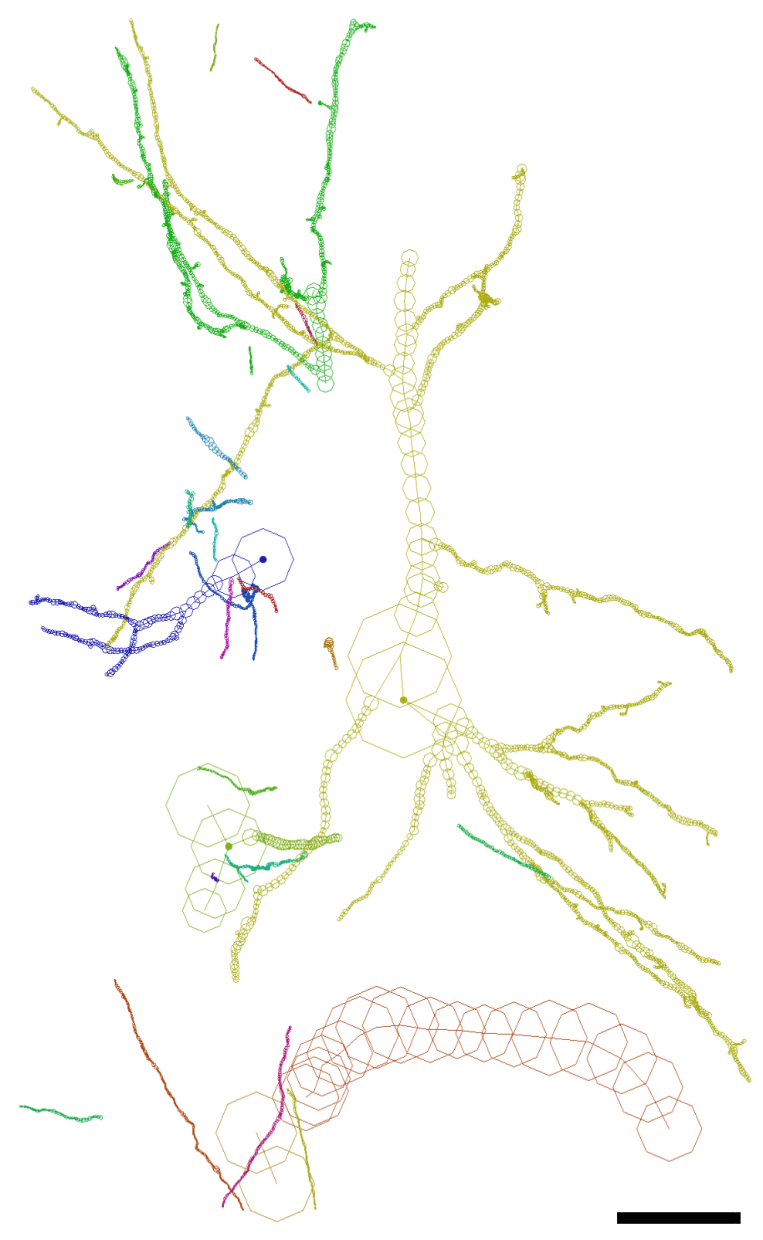


(**u**) S3I structure. (**v**) S3J structure.

**Supplementary Figure S3 (cont'd).** Cartesian coordinate models of schizophrenia tissue structures. The pial surface is toward the top. The models were drawn with the MCTrace software. Constituents of the models are color-coded. Nodes composing each constituent are indicated with circles. Dots indicate somata nodes. Scale bars: 20 μm.


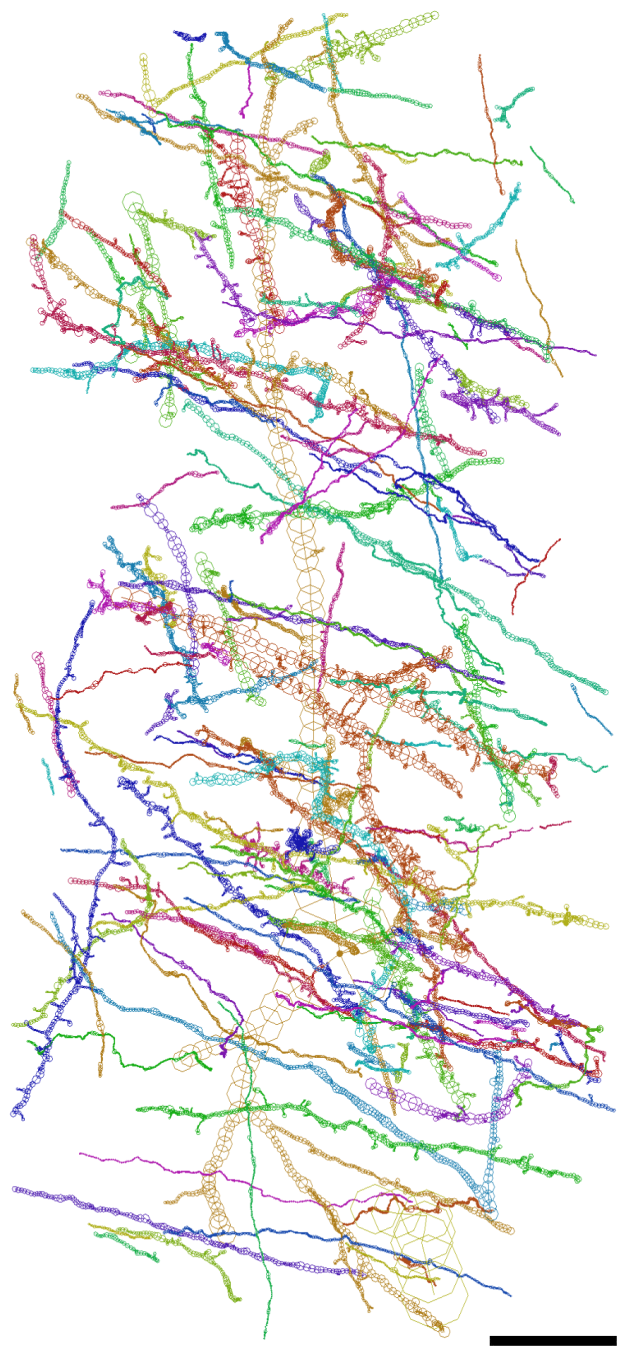

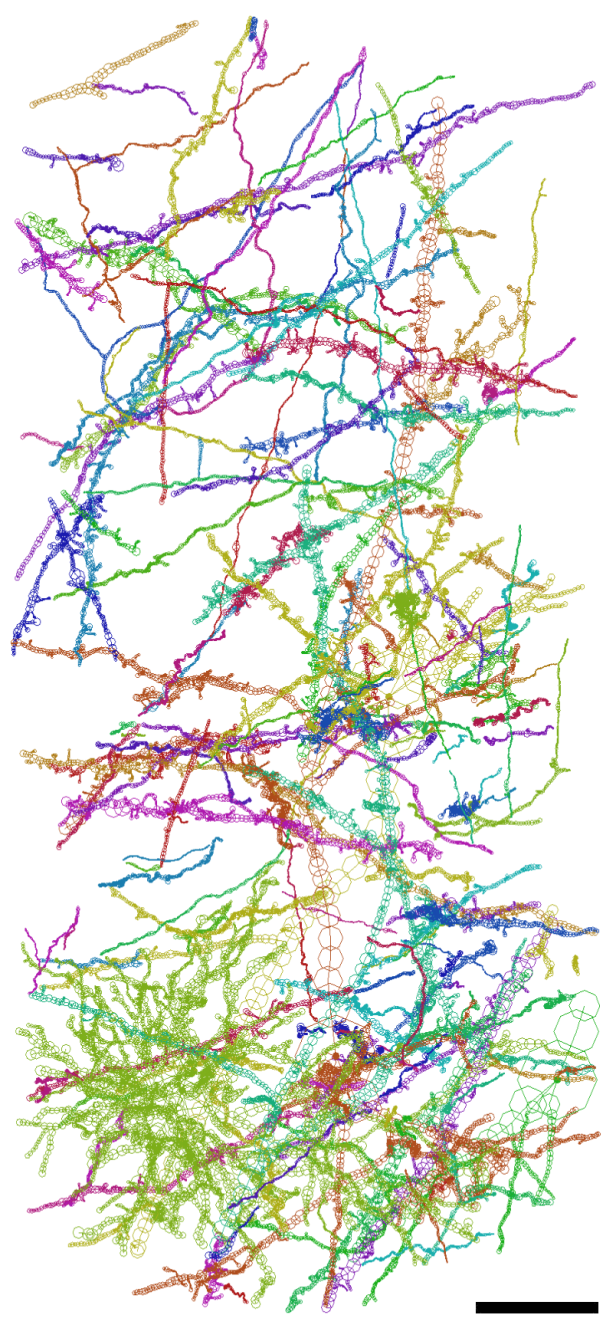


(**w**) S4A structure. (**x**) S4B structure.

**Supplementary Figure S3 (cont'd).** Cartesian coordinate models of schizophrenia tissue structures. The pial surface is toward the top. The models were drawn with the MCTrace software. Constituents of the models are color-coded. Nodes composing each constituent are indicated with circles. Dots indicate somata nodes. Scale bars: 20 μm.


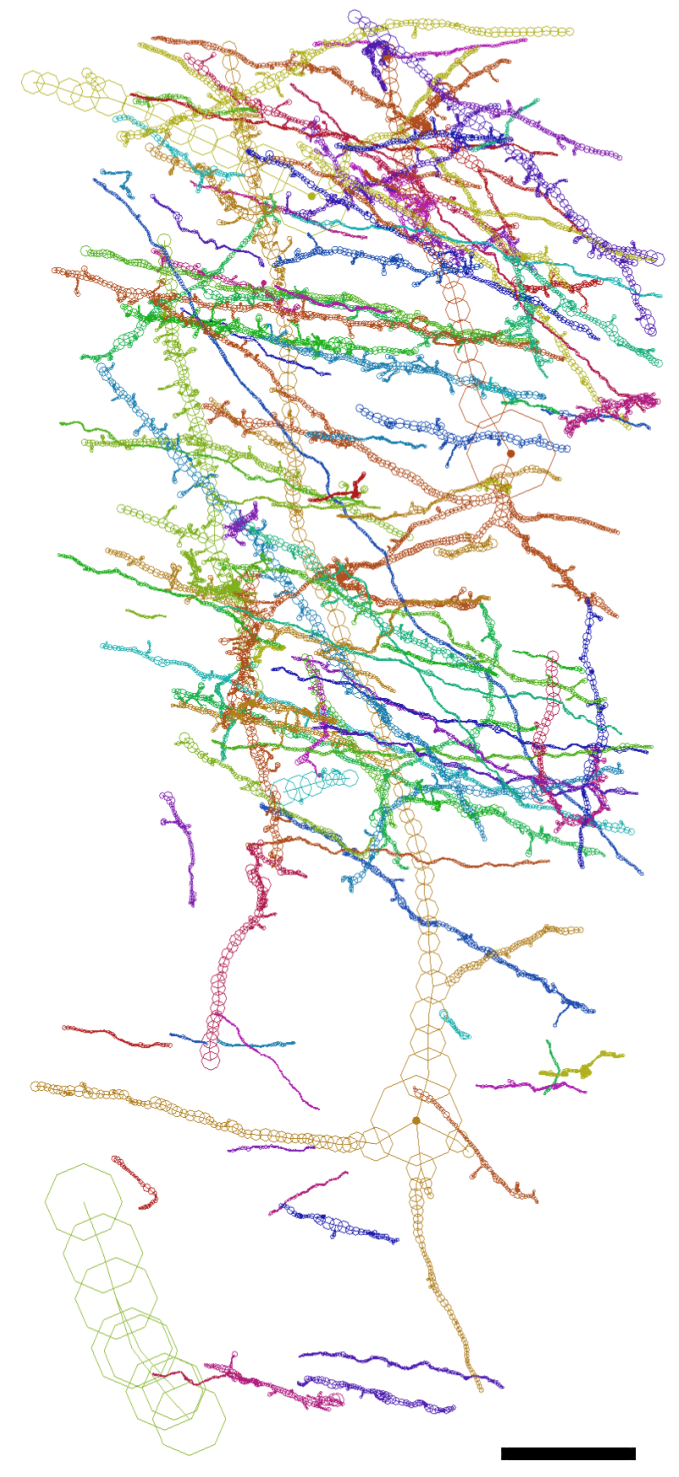


(**y**) S4D structure.

**Supplementary Figure S3 (cont'd).** Cartesian coordinate models of schizophrenia tissue structures. The pial surface is toward the top. The models were drawn with the MCTrace software. Constituents of the models are color-coded. Nodes composing each constituent are indicated with circles. Dots indicate somata nodes. Scale bars: 20 μm.


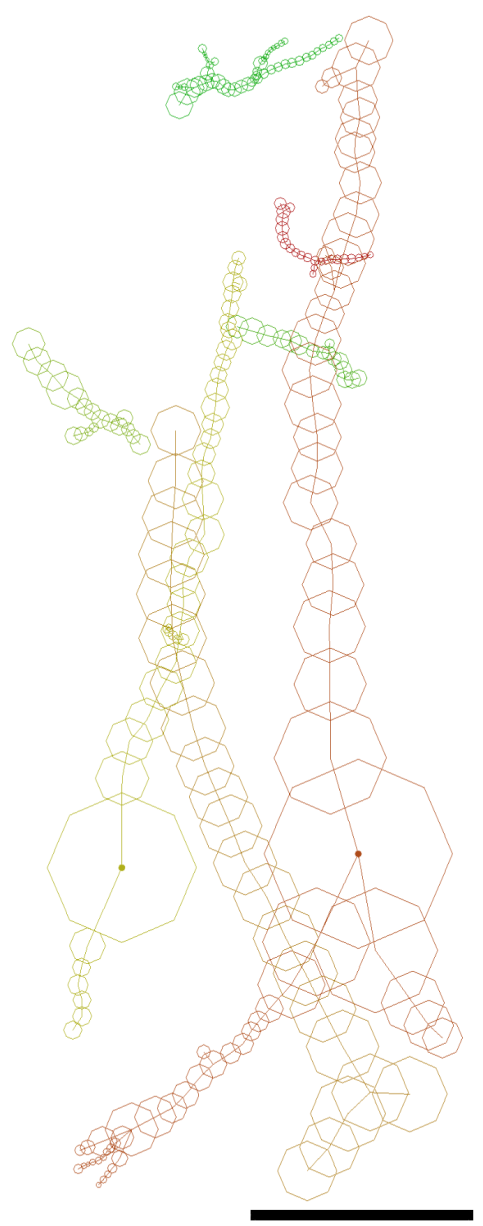

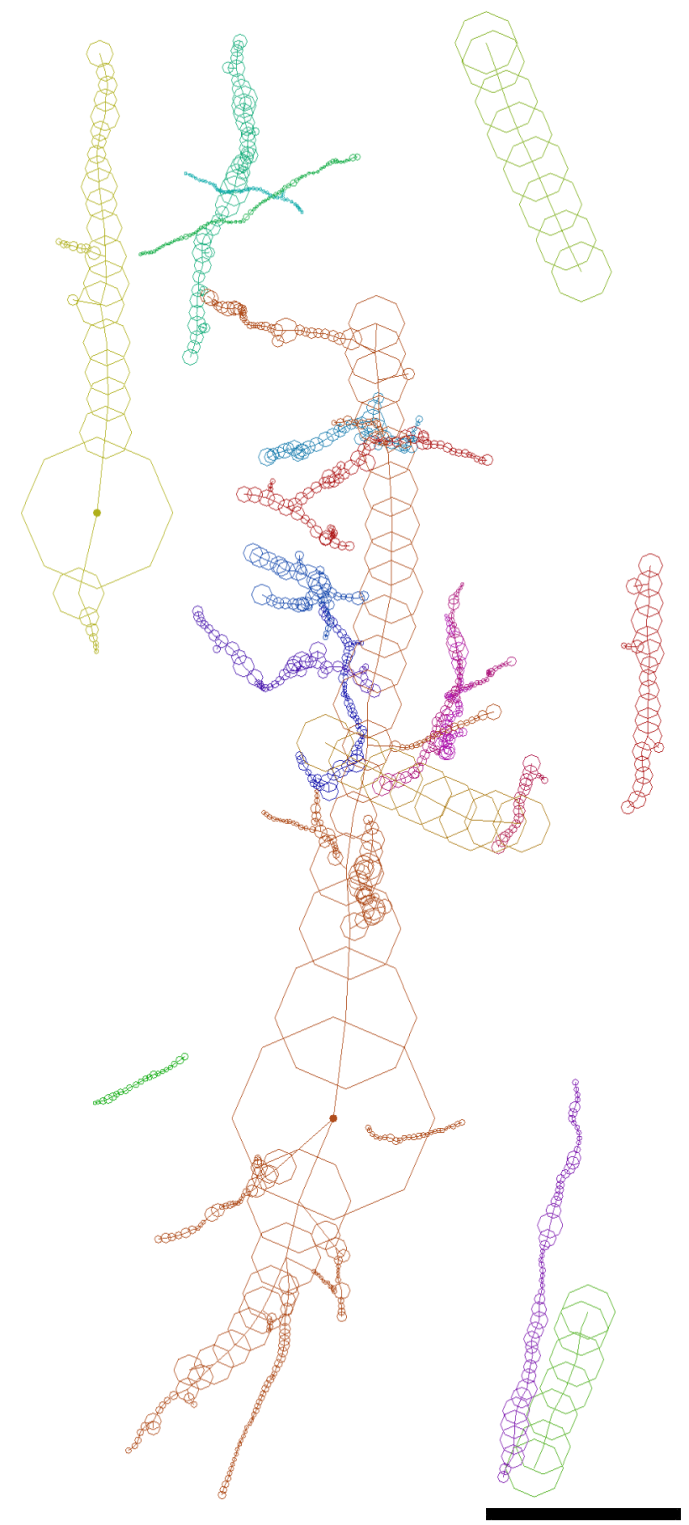


(**a**) N1A structure. (**b**) N1B structure.

**Supplementary Figure S4.** Cartesian coordinate models of control tissue structures. The pial surface is toward the top. The models were drawn with the MCTrace software. Constituents of the models are color-coded. Nodes composing each constituent are indicated with circles. Dots indicate somata nodes. Scale bars: 20 μm.


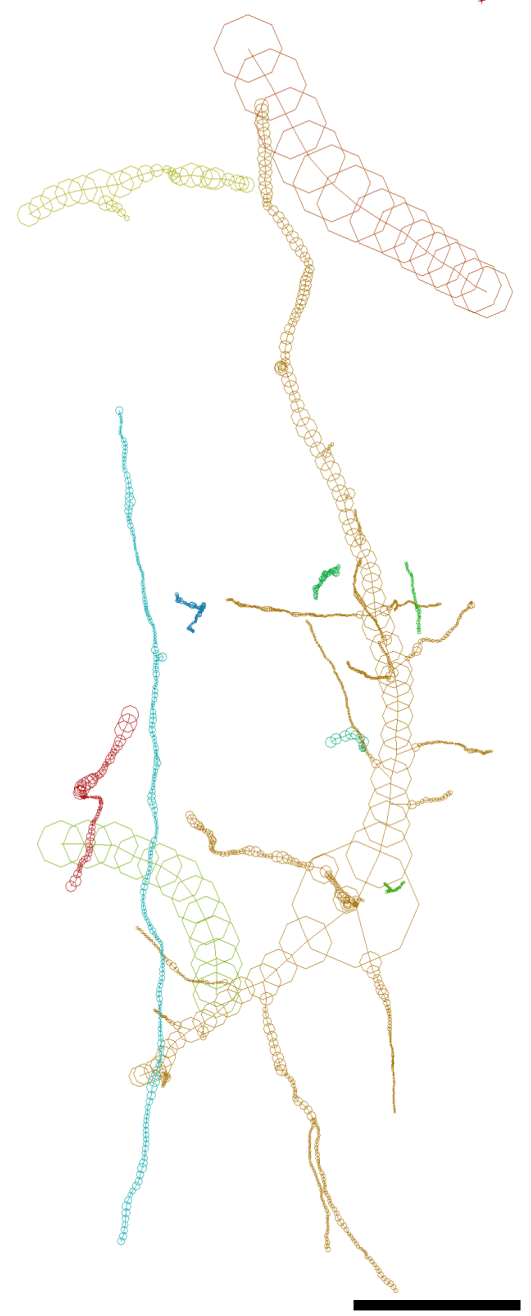

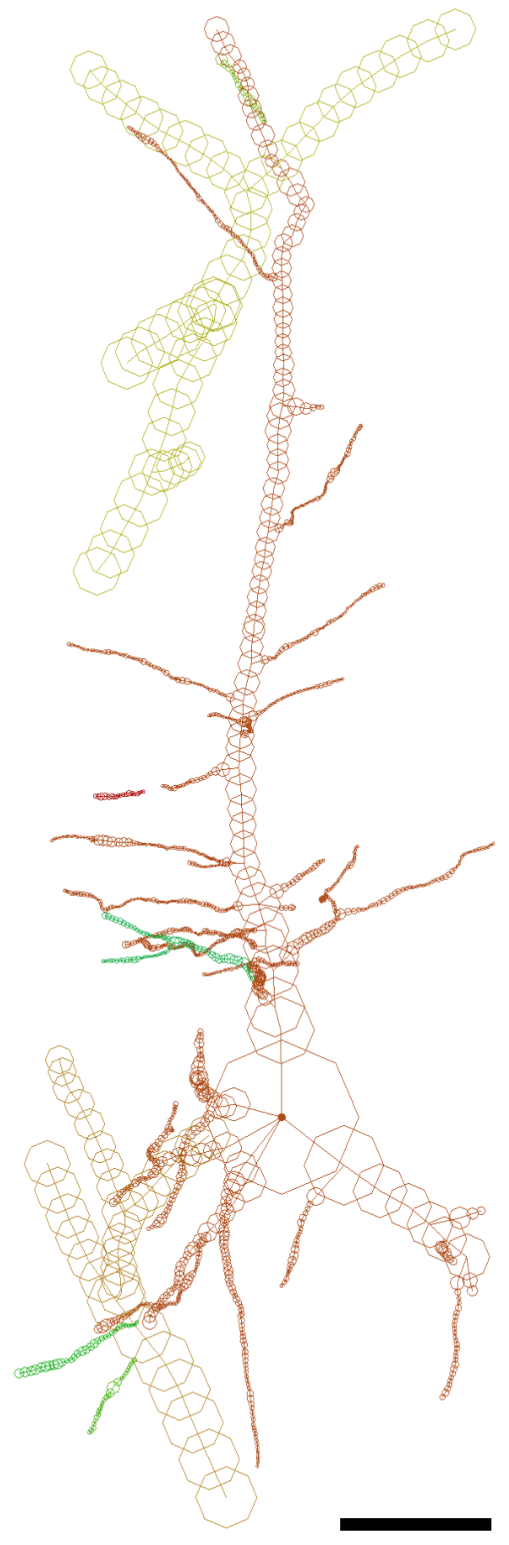


(**c**) N1C structure. (**d**) N1D structure.

**Supplementary Figure S4 (cont'd).** Cartesian coordinate models of control tissue structures. The pial surface is toward the top. The models were drawn with the MCTrace software. Constituents of the models are color-coded. Nodes composing each constituent are indicated with circles. Dots indicate somata nodes. Scale bars: 20 μm.


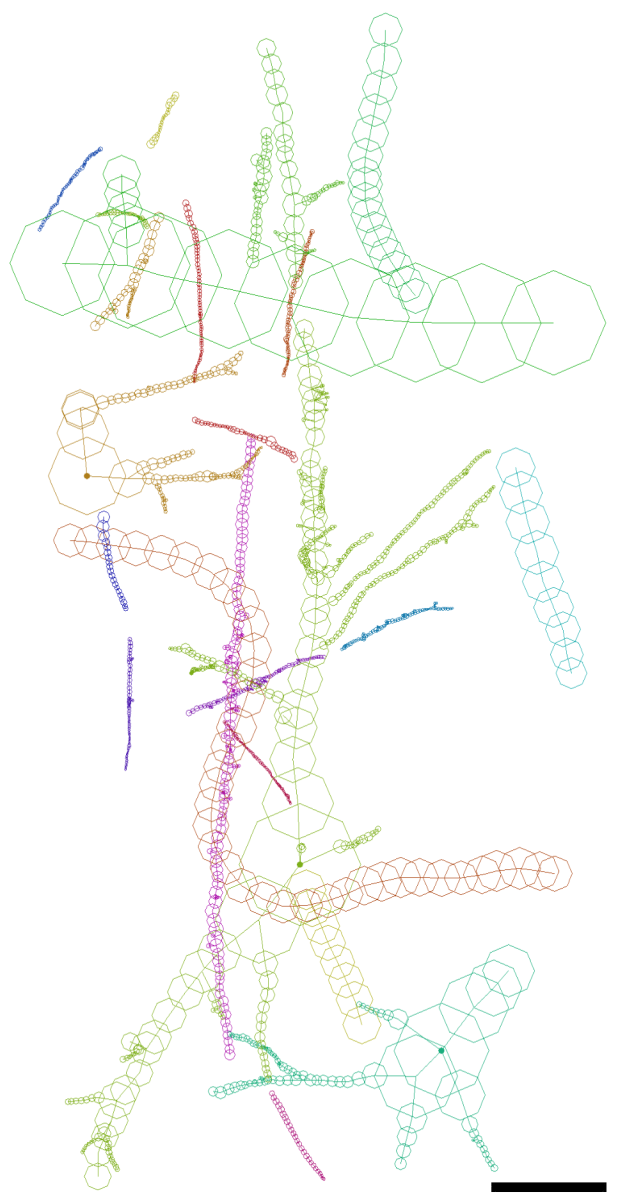

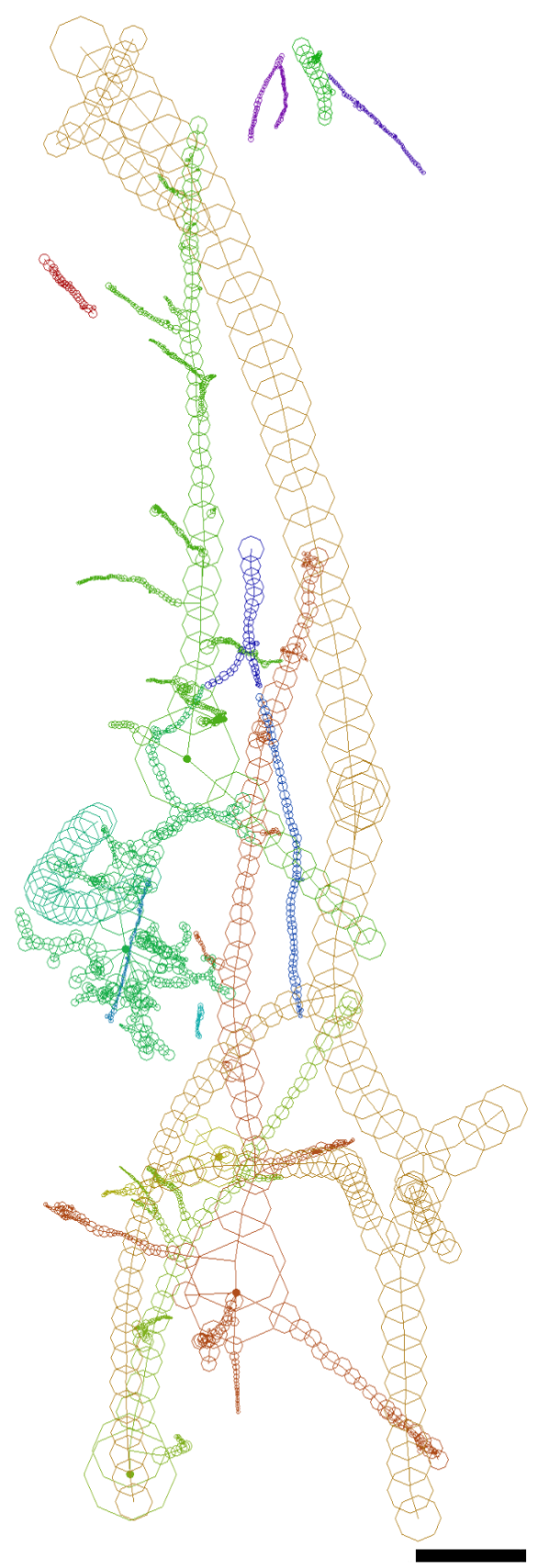


(**e**) N1F structure. (**f**) N1G structure.

**Supplementary Figure S4 (cont'd).** Cartesian coordinate models of control tissue structures. The pial surface is toward the top. The models were drawn with the MCTrace software. Constituents of the models are color-coded. Nodes composing each constituent are indicated with circles. Dots indicate somata nodes. Scale bars: 20 μm.


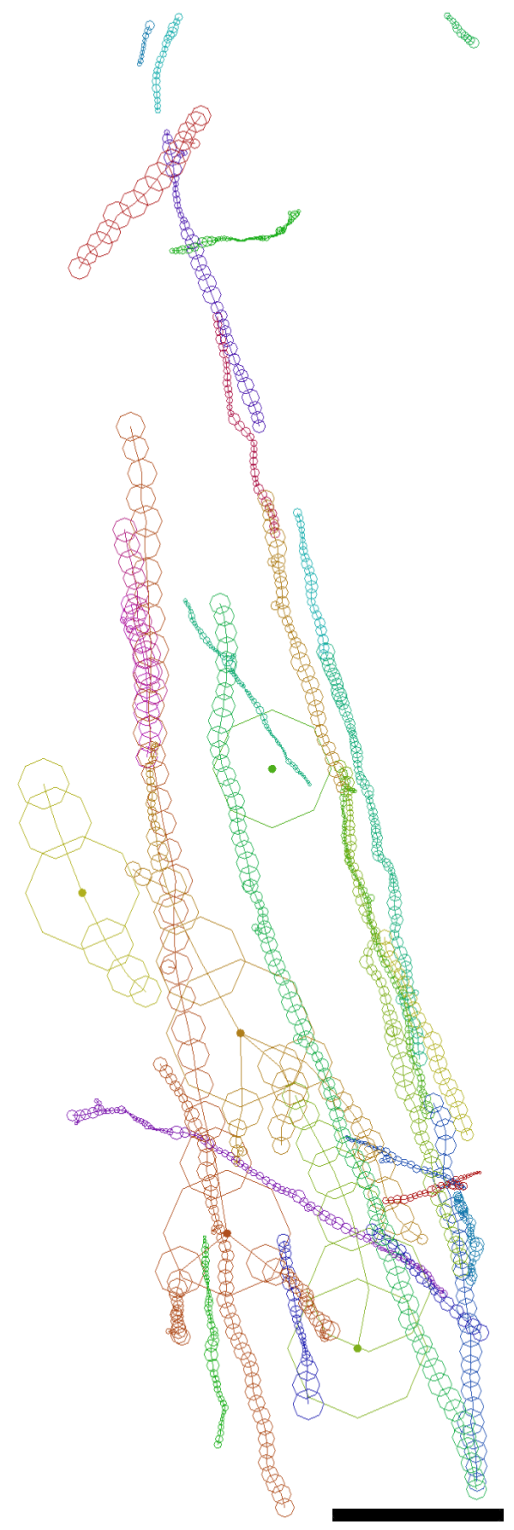

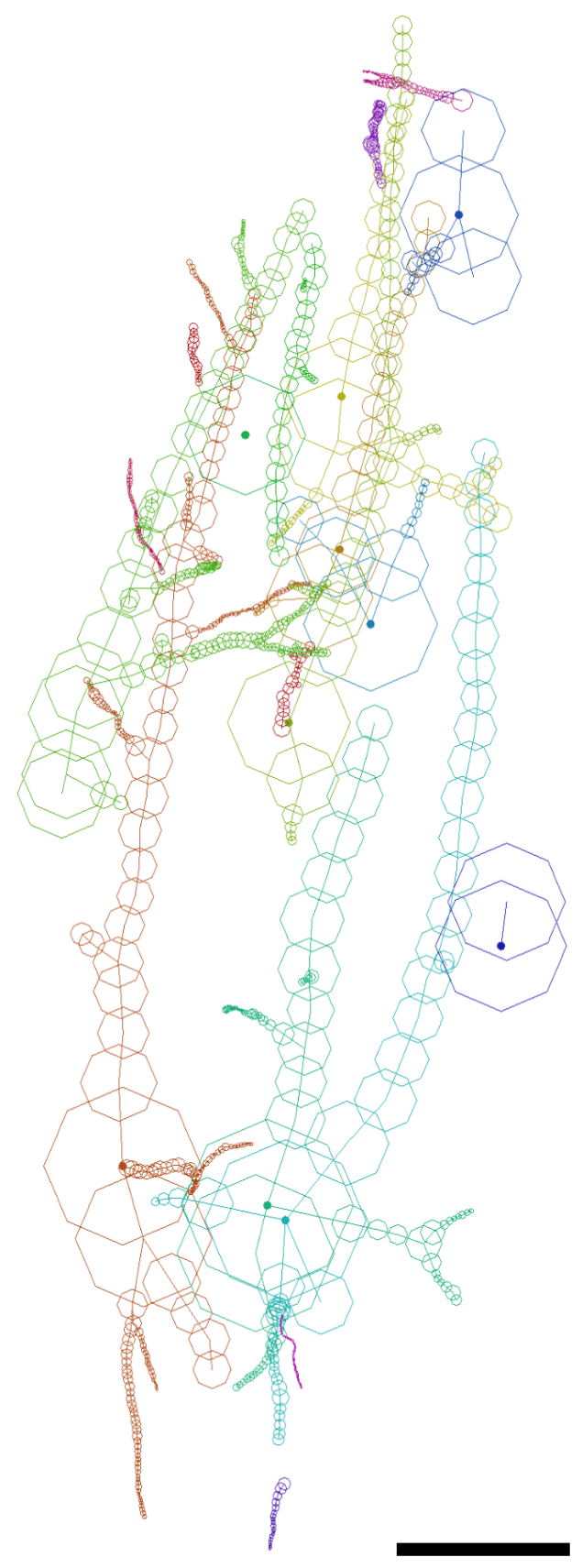


(**g**) N1H structure. (**h**) N1I structure.

**Supplementary Figure S4 (cont'd).** Cartesian coordinate models of control tissue structures. The pial surface is toward the top. The models were drawn with the MCTrace software. Constituents of the models are color-coded. Nodes composing each constituent are indicated with circles. Dots indicate somata nodes. Scale bars: 20 μm.


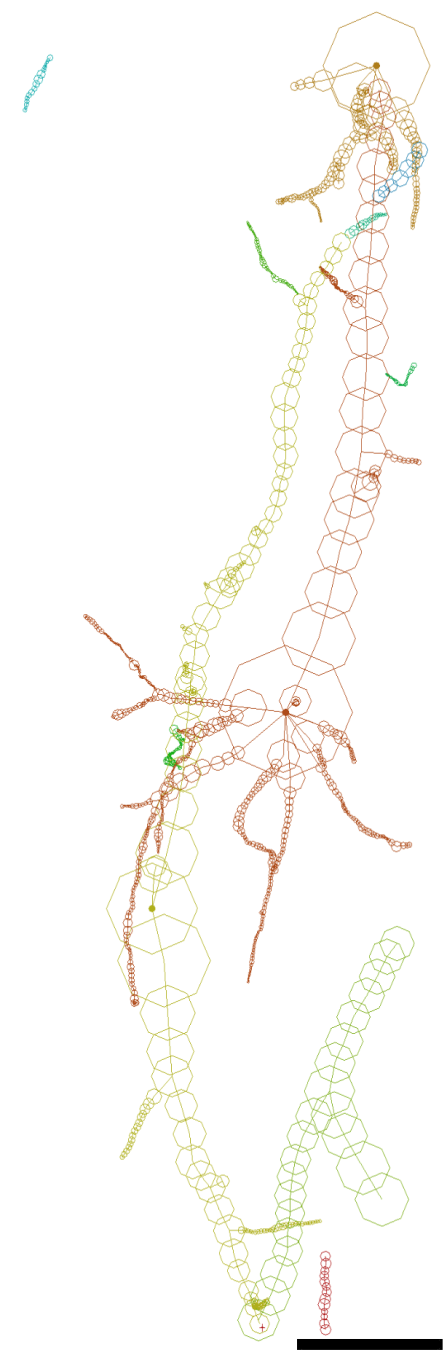

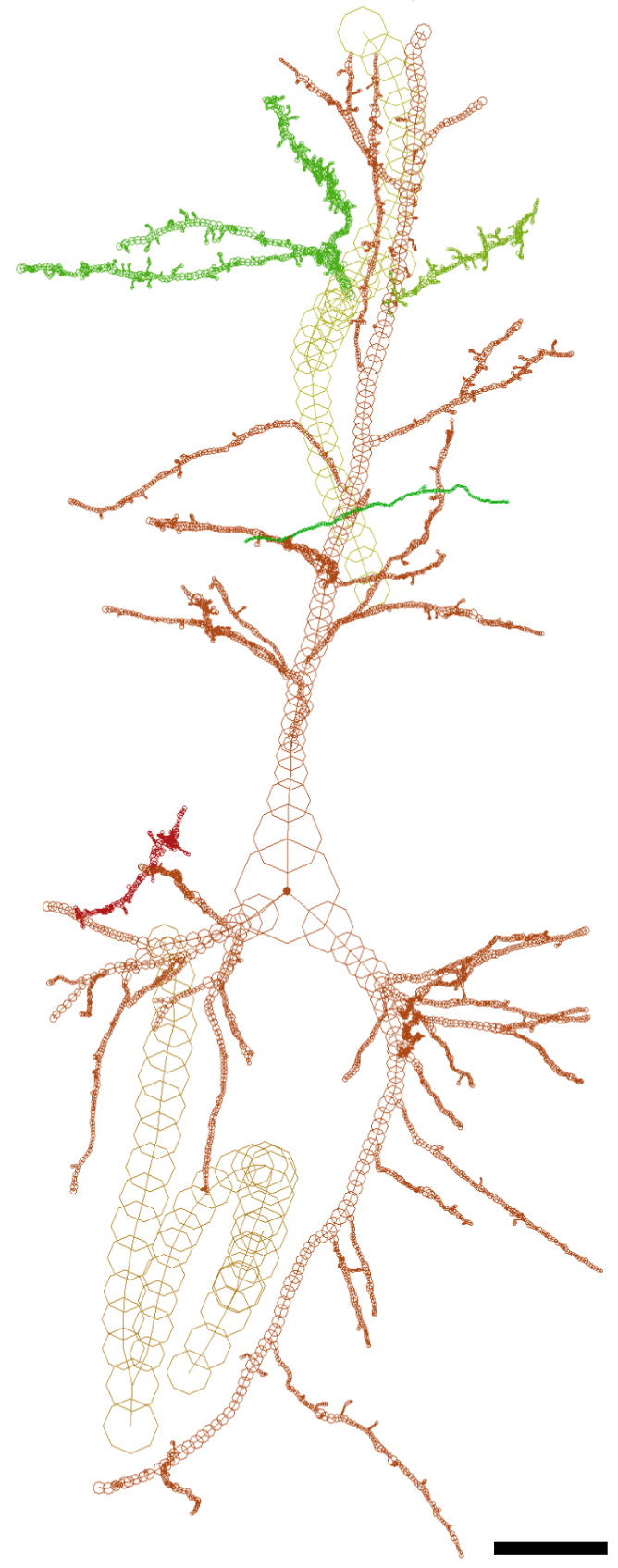


(**i**) N1J structure. (**j**) N2A structure.

**Supplementary Figure S4 (cont'd).** Cartesian coordinate models of control tissue structures. The pial surface is toward the top. The models were drawn with the MCTrace software. Constituents of the models are color-coded. Nodes composing each constituent are indicated with circles. Dots indicate somata nodes. Scale bars: 20 μm.


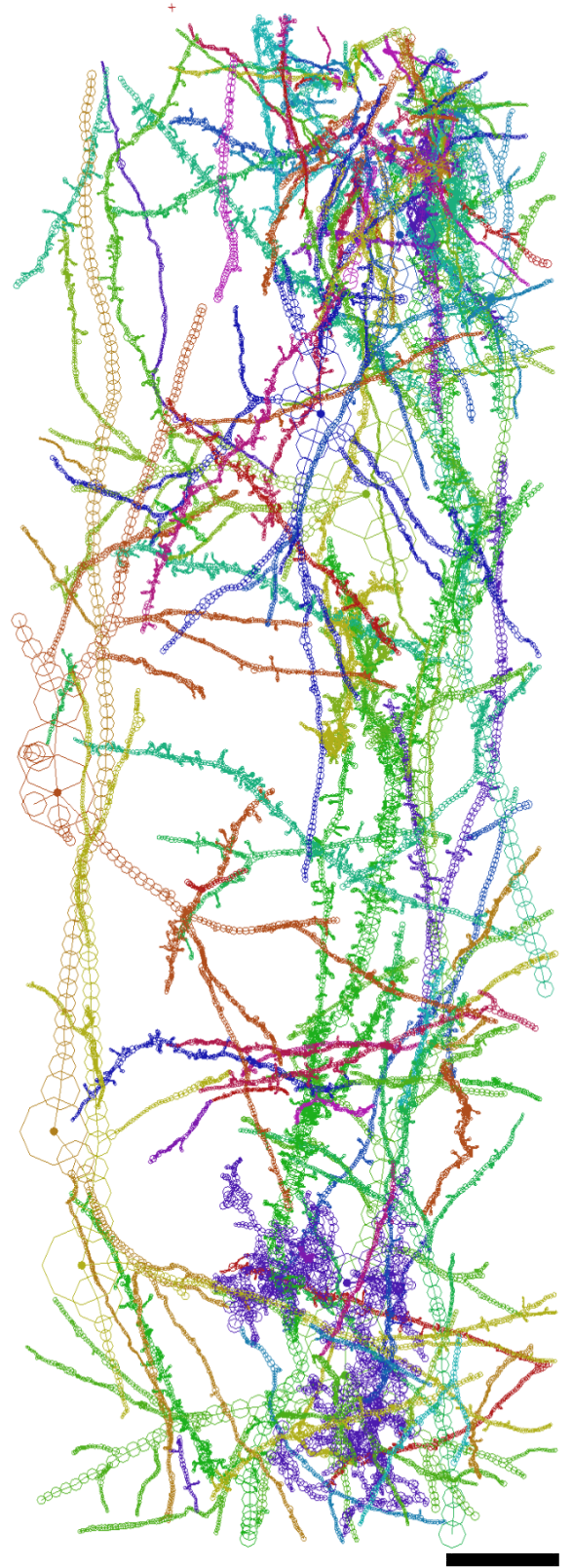

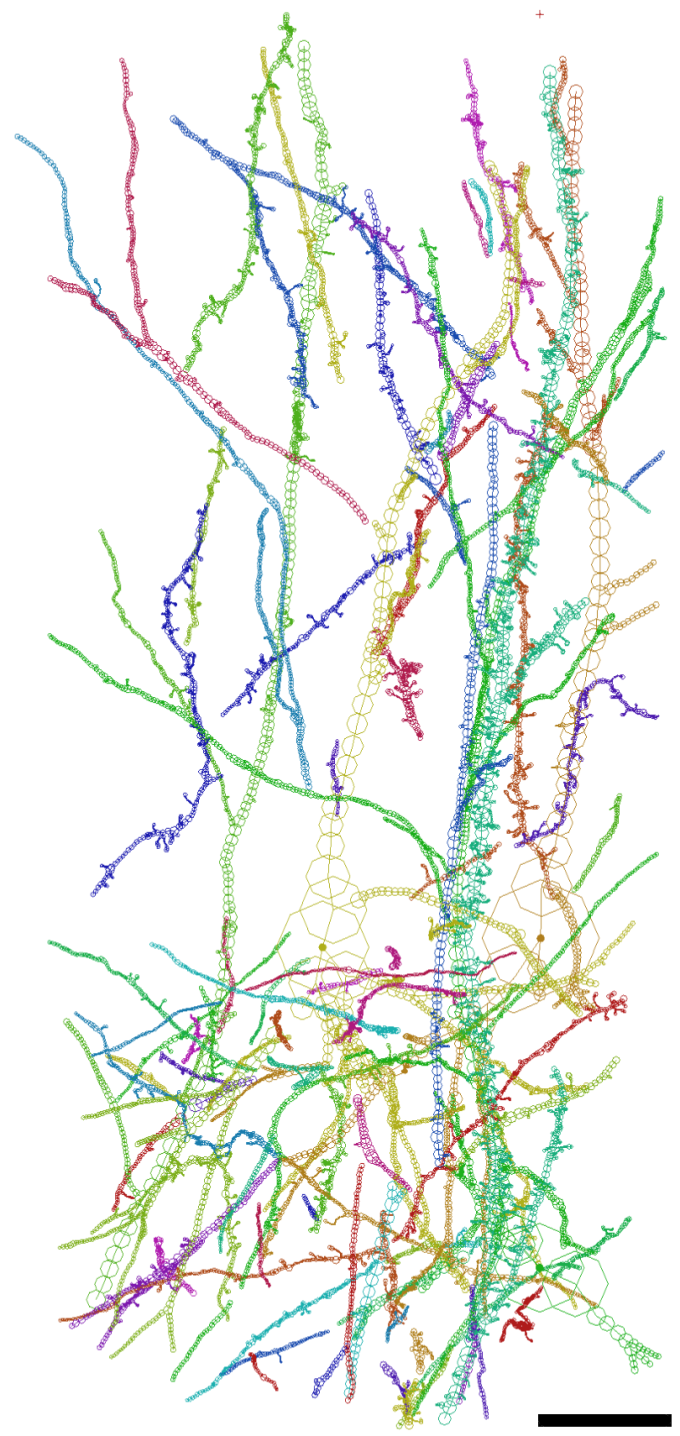


(**k**) N2B structure. (**l**) N2D structure.

**Supplementary Figure S4 (cont'd).** Cartesian coordinate models of control tissue structures. The pial surface is toward the top. The models were drawn with the MCTrace software. Constituents of the models are color-coded. Nodes composing each constituent are indicated with circles. Dots indicate somata nodes. Scale bars: 20 μm.


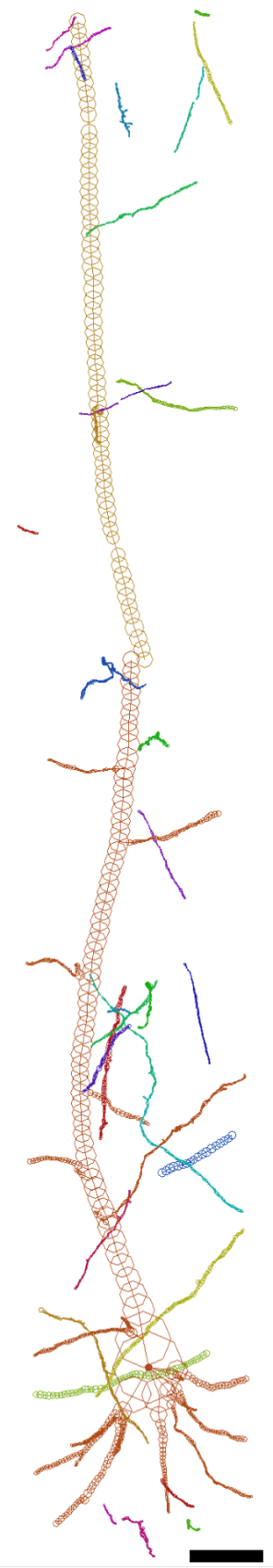

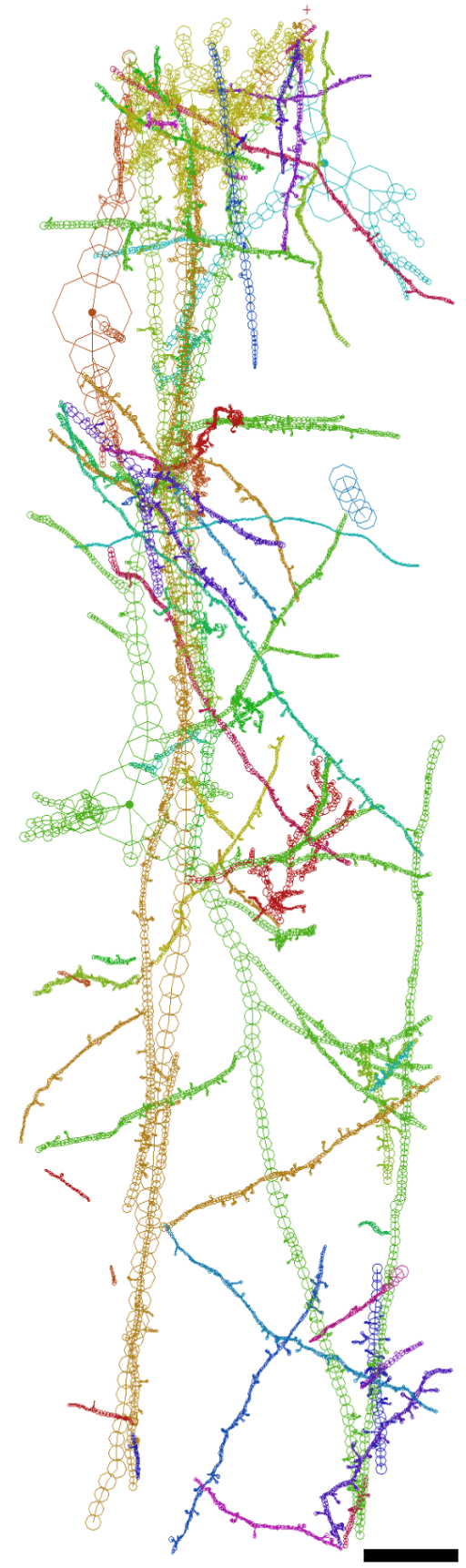


(**m**) N3A structure. (**n**) N3B structure.

**Supplementary Figure S4 (cont'd).** Cartesian coordinate models of control tissue structures. The pial surface is toward the top. The models were drawn with the MCTrace software. Constituents of the models are color-coded. Nodes composing each constituent are indicated with circles. Dots indicate somata nodes. Scale bars: 20 μm.


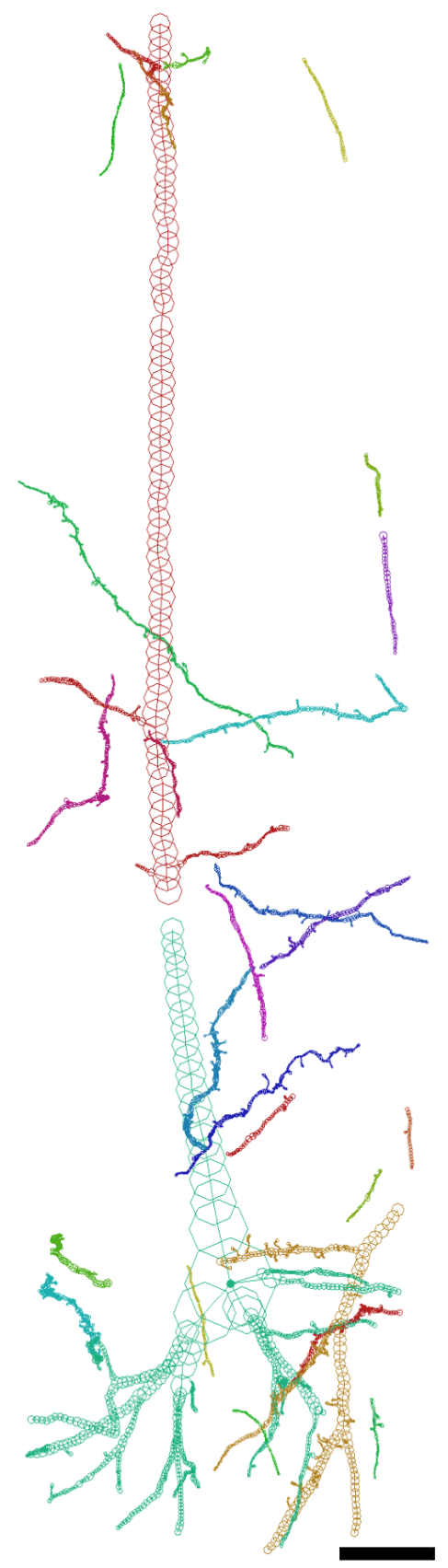

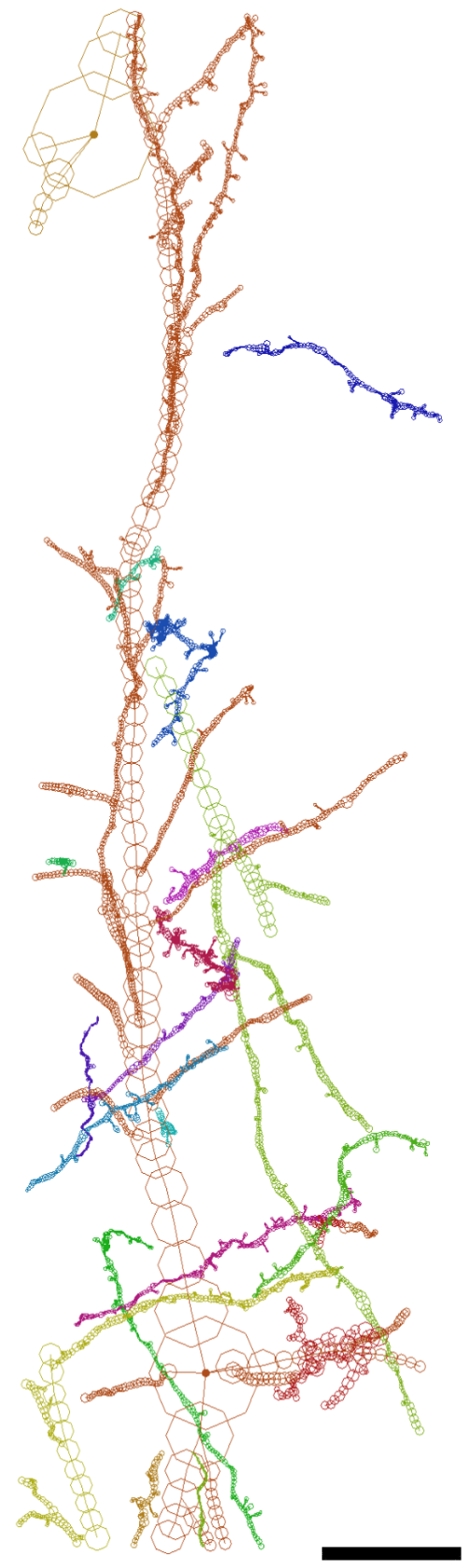


(**o**) N3C structure. (**p**) N3E structure.

**Supplementary Figure S4 (cont'd).** Cartesian coordinate models of control tissue structures. The pial surface is toward the top. The models were drawn with the MCTrace software. Constituents of the models are color-coded. Nodes composing each constituent are indicated with circles. Dots indicate somata nodes. Scale bars: 20 μm.


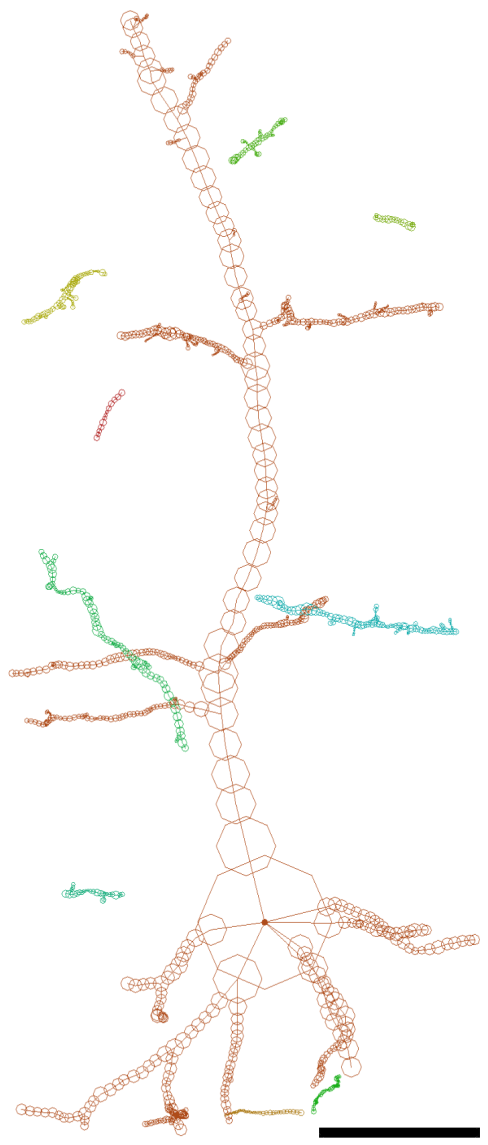

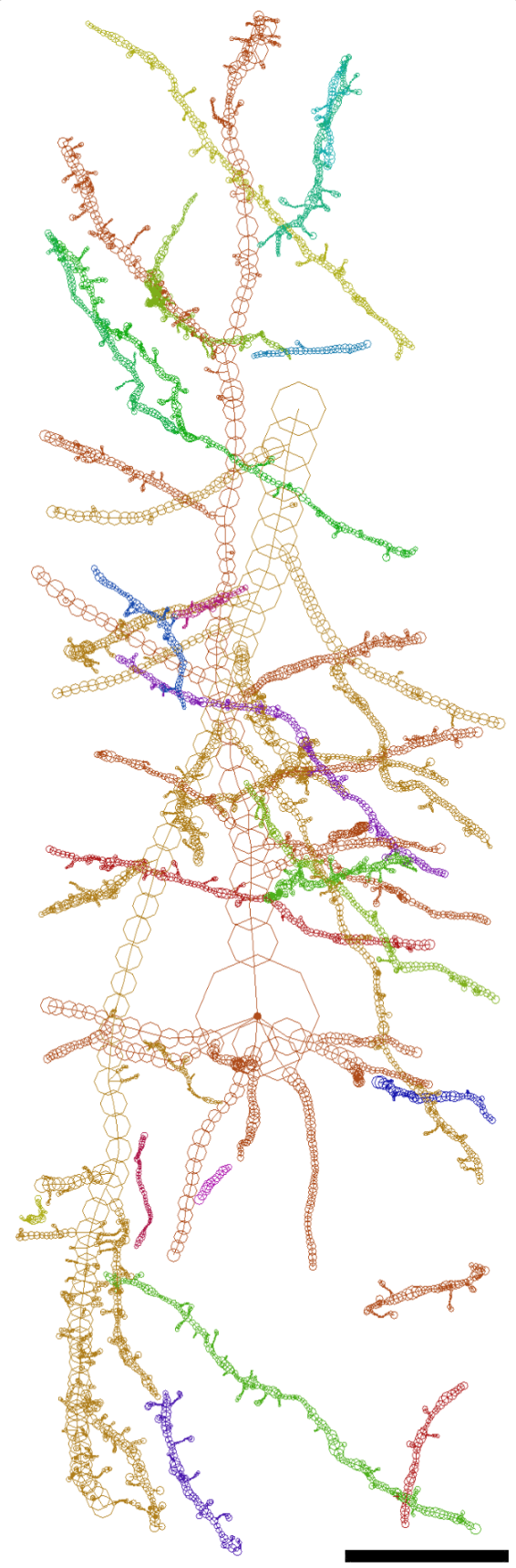


(**q**) N3F structure. (**r**) N3G structure.

**Supplementary Figure S4 (cont'd).** Cartesian coordinate models of control tissue structures. The pial surface is toward the top. The models were drawn with the MCTrace software. Constituents of the models are color-coded. Nodes composing each constituent are indicated with circles. Dots indicate somata nodes. Scale bars: 20 μm.


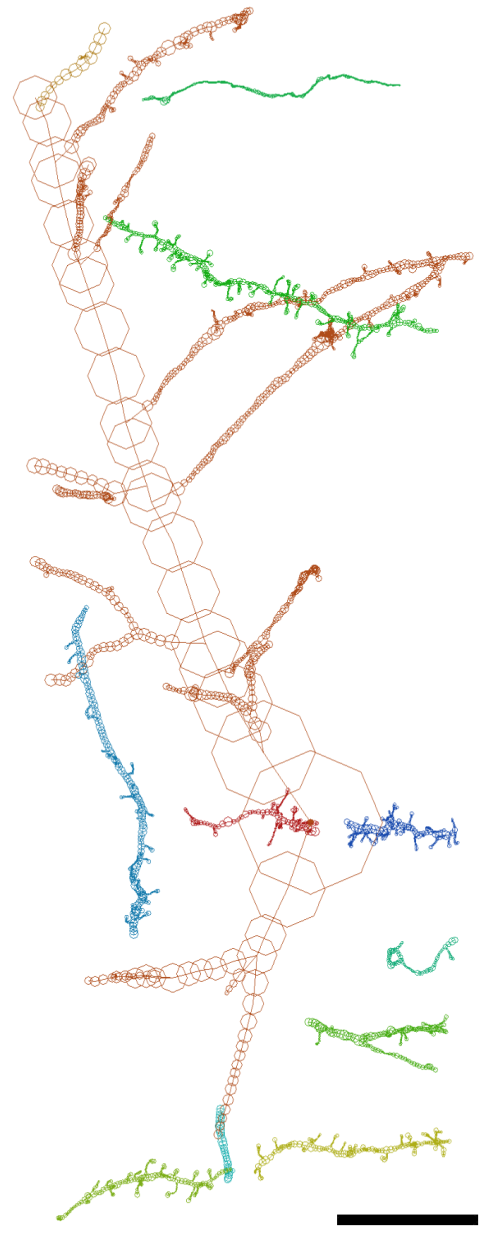

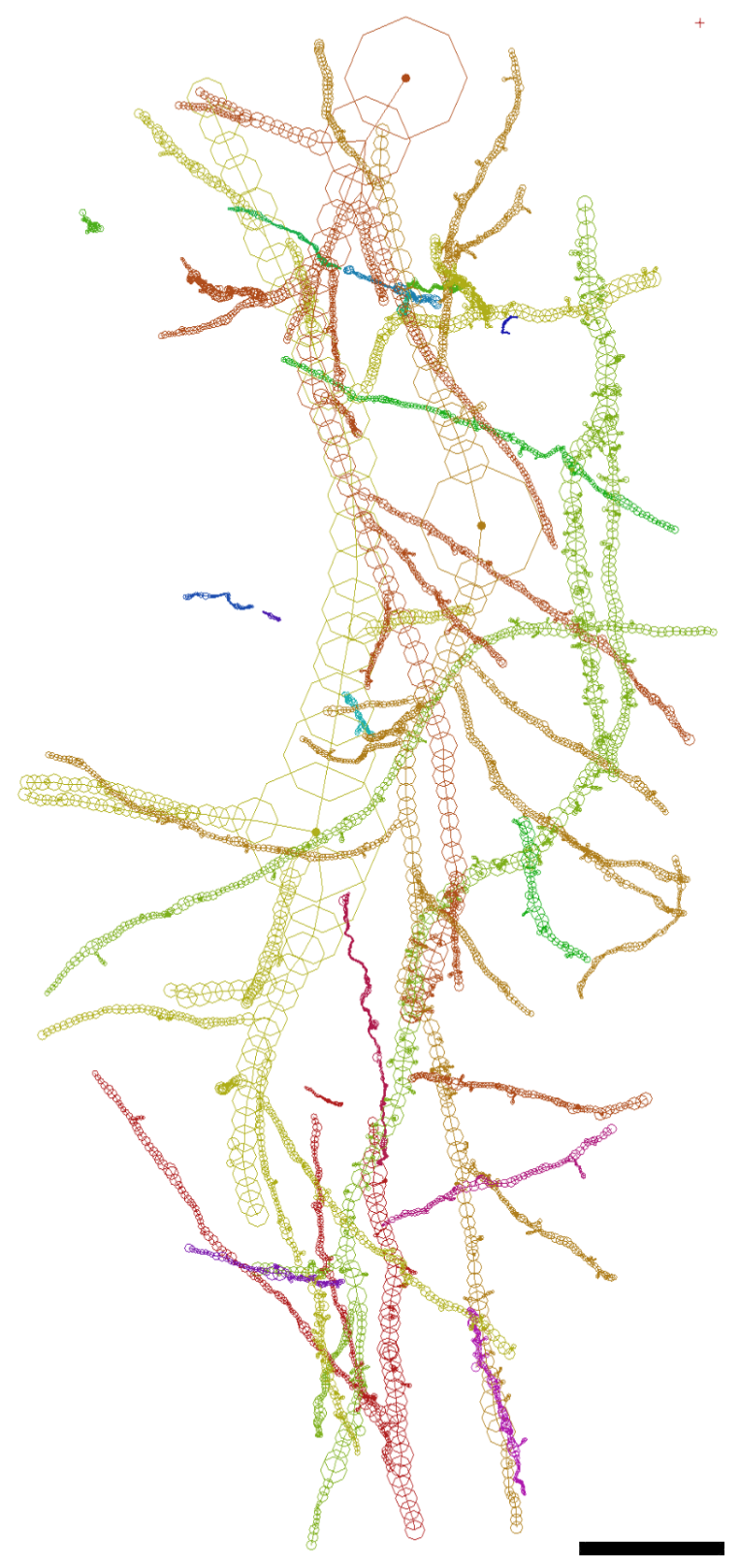


(**s**) N3H structure. (**t**) N4B structure.

**Supplementary Figure S4 (cont'd).** Cartesian coordinate models of control tissue structures. The pial surface is toward the top. The models were drawn with the MCTrace software. Constituents of the models are color-coded. Nodes composing each constituent are indicated with circles. Dots indicate somata nodes. Scale bars: 20 μm.


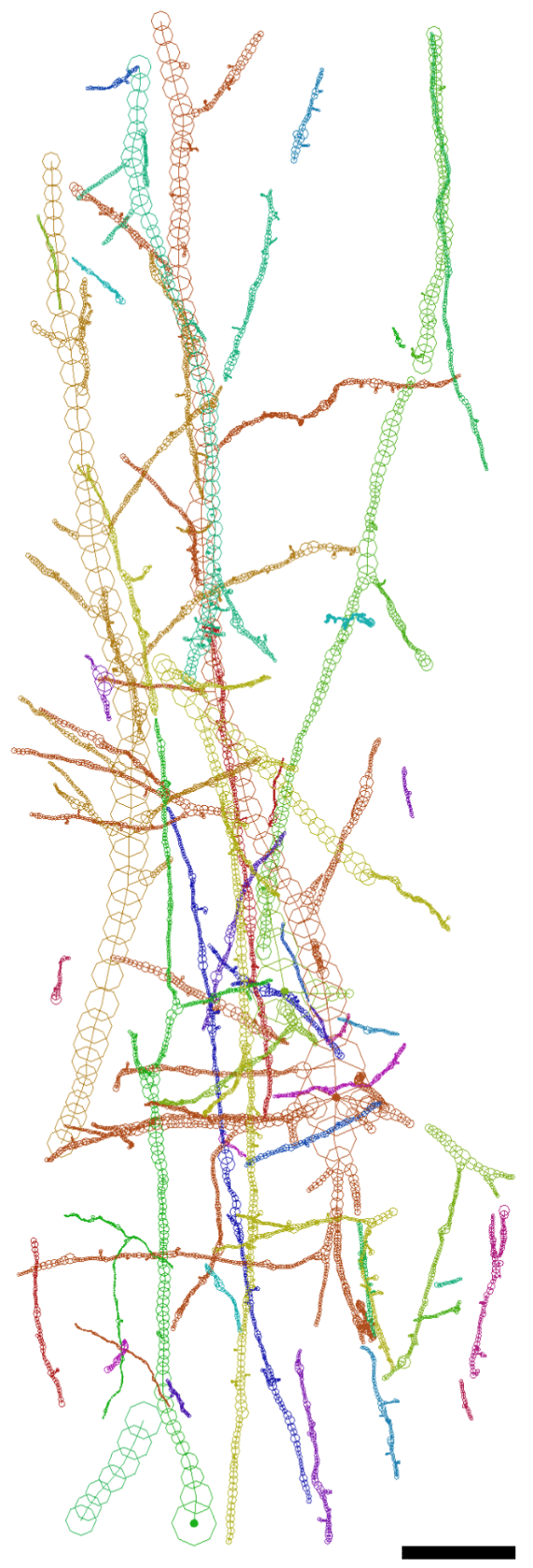

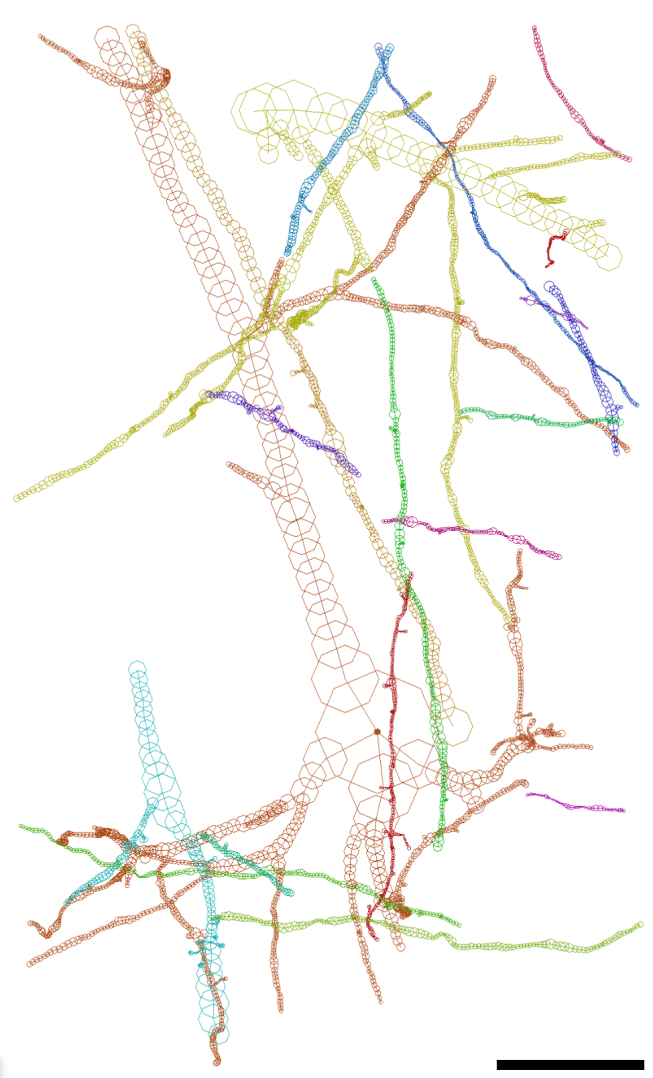


(**u**) N4C structure. (**v**) N4D structure.

**Supplementary Figure S4 (cont'd).** Cartesian coordinate models of control tissue structures. The pial surface is toward the top. The models were drawn with the MCTrace software. Constituents of the models are color-coded. Nodes composing each constituent are indicated with circles. Dots indicate somata nodes. Scale bars: 20 μm.

**a b**


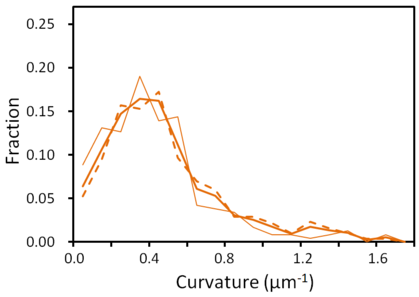

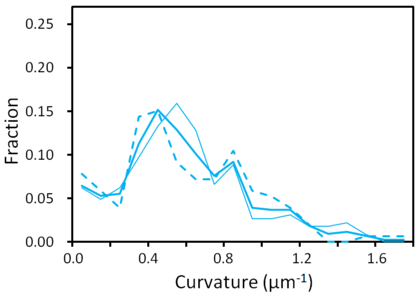


**c d**


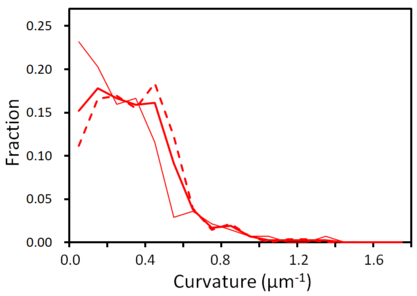

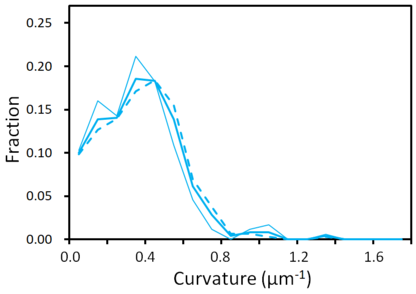


**Supplementary Figure S5.** Relative frequency of neurite in each 0.1 μm^-1^ bin of curvature. (**a**) Relative frequencies of the samples of the S2 case are separately plotted. The dashed line corresponds to datasets S2A–S2E, and the thin line to S2F–S2J. The bold line shows the overall frequency. (**b**) Relative frequency of samples of S3. The dashed line corresponds to datasets S3A–S3E, and the thin line to S3F–S3I. (**c**) Relative frequency of samples of N1. The dashed line corresponds to datasets N1A–N1G, and the thin line to N1H–N1J. (**d**) Relative frequency of samples of N3. The dashed line corresponds to datasets N3A–N3D, and the thin line to N3E–N3H.

**a**


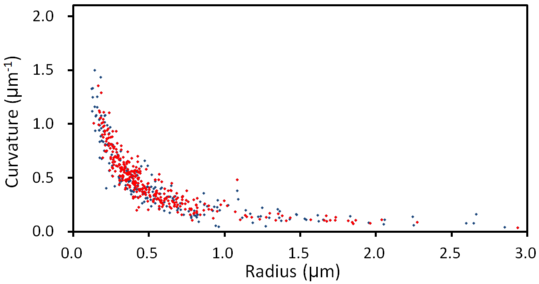


**b c**


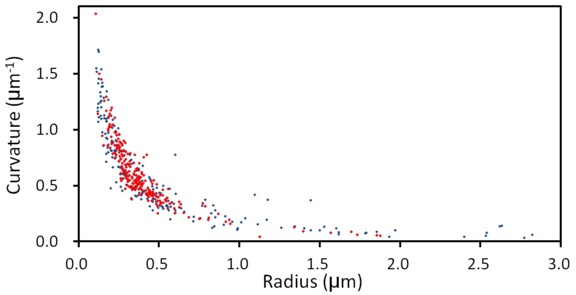

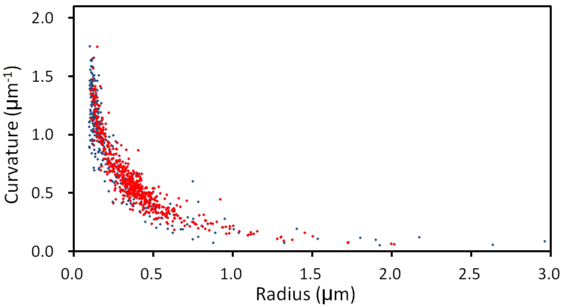


**d**


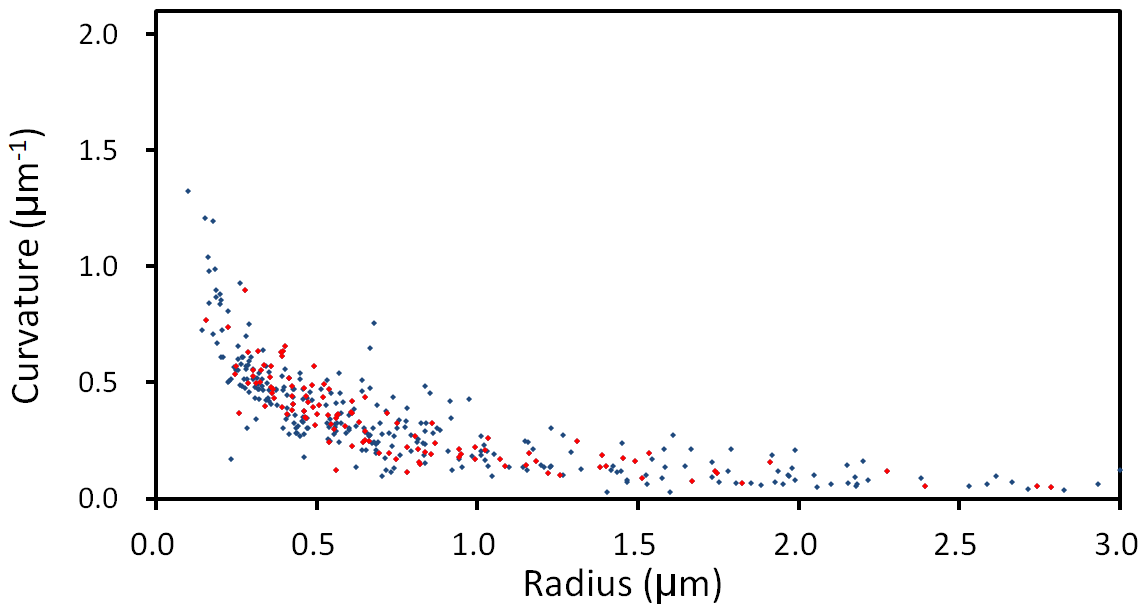


**e f**


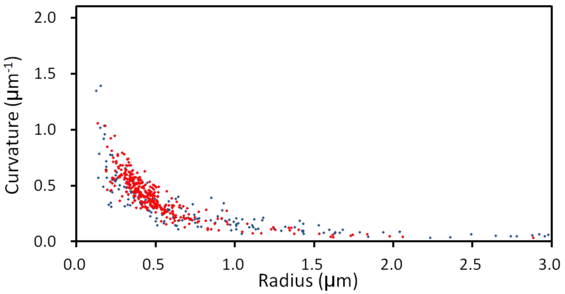

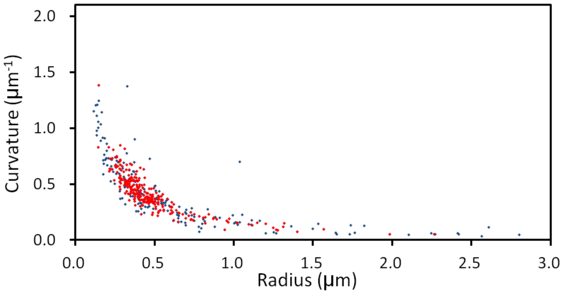


**Supplementary Figure S6.** Scatter plots of curvature and mean radius of neurites. Spiny dendrites are indicated with red dots and smooth neurites with blue. Neurites of which mean radii are larger than 3 μm are omitted. (**a**) S1. (**b**) S3. (**c**) S4. (**d**) N1. (**e**) N3. (**f**) N4.

**a b**


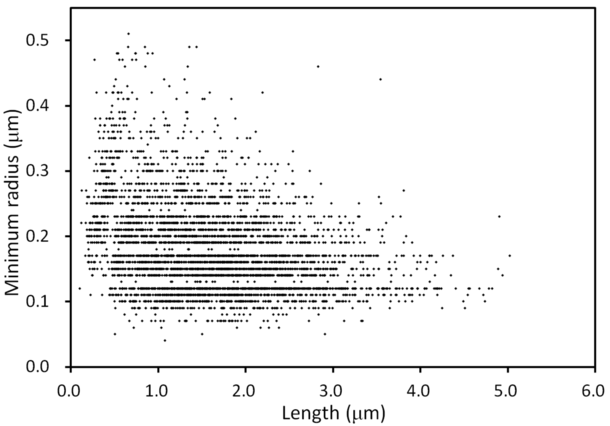

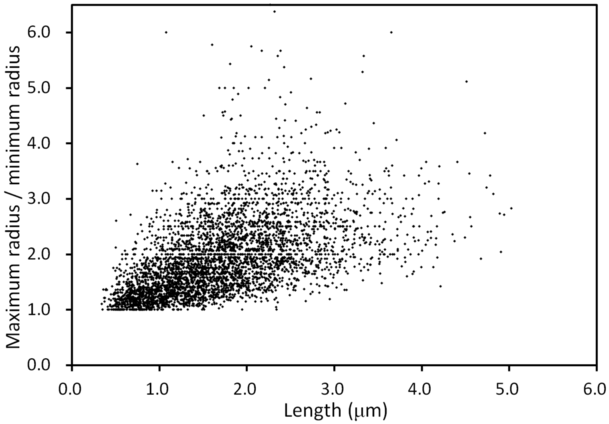


**c d**


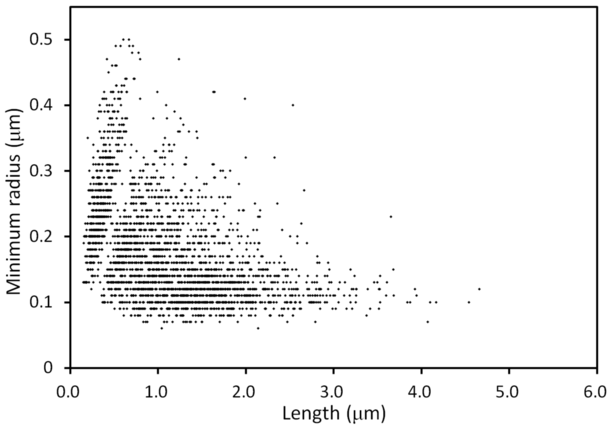

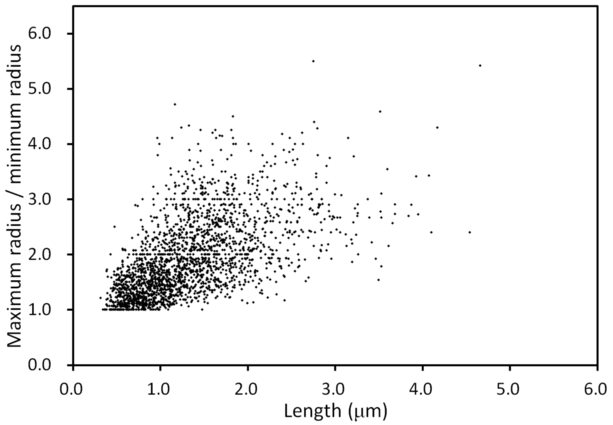


**e f**


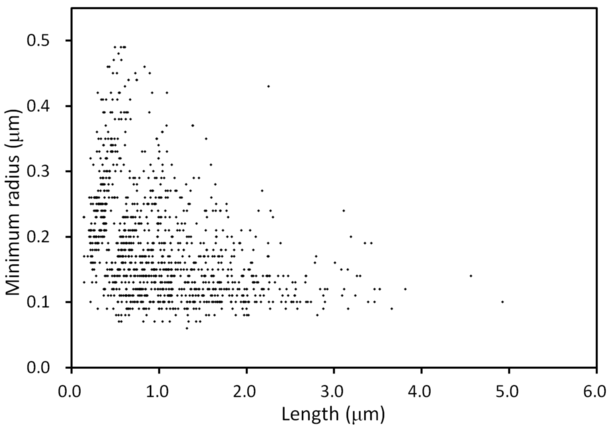

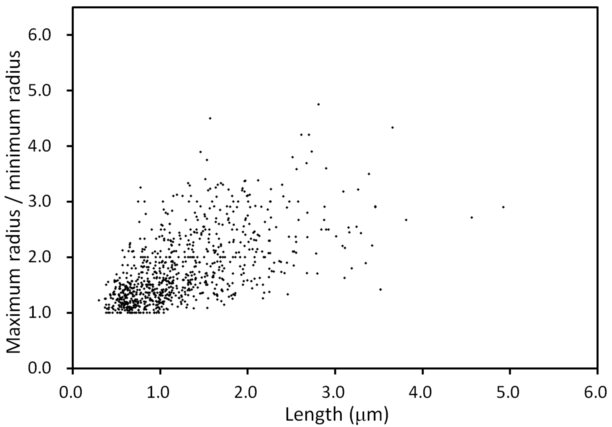


**Supplementary Figure S7.** Scatter plot of spine parameters. Minimum node radius of spine and ratio between maximum / minimum radii are plotted against spine length. (**a**) Plot of the minimum radius of the S1 case. An outlier (length/radius = 6.24/0.12) was omitted. (**b**) Radius ratio of S1. Four outliers (length/ratio = 6.24/3.58, 4.25/6.67, 2.91/9.60, and 2.27/6.50) were omitted. (**c**) Minimum radius of S2. (**d**) Radius ratio of S2. (**e**) Minimum radius of S3. (**f**) Radius ratio of S3.

**g h**


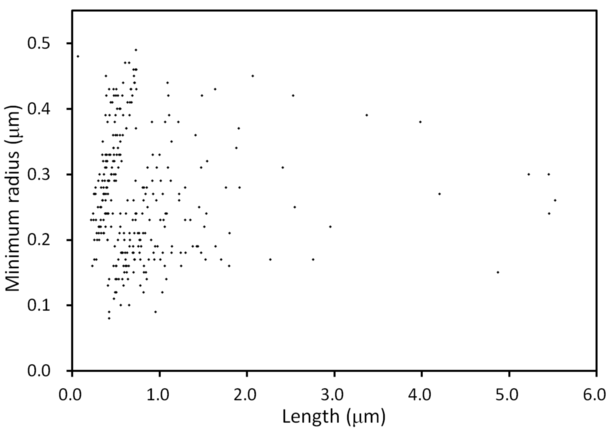

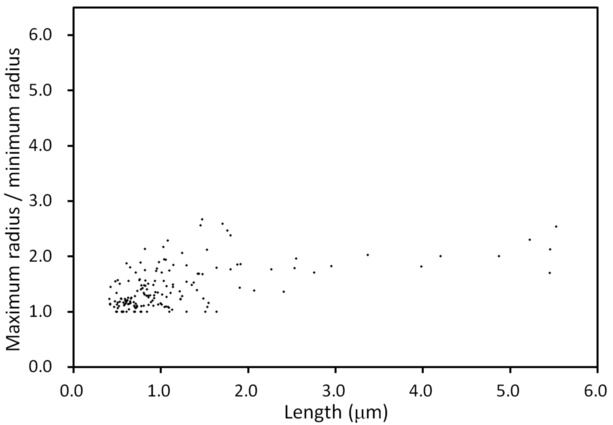


**i j**


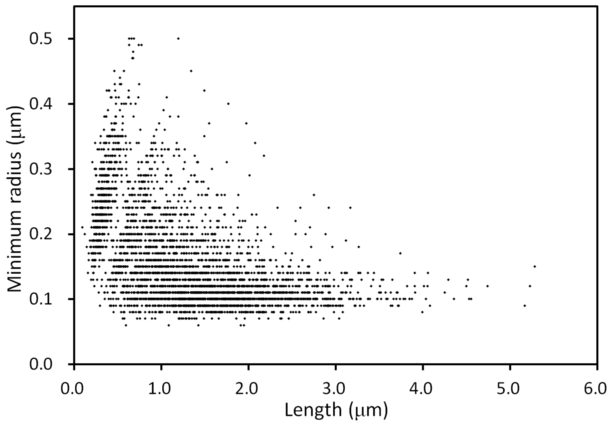

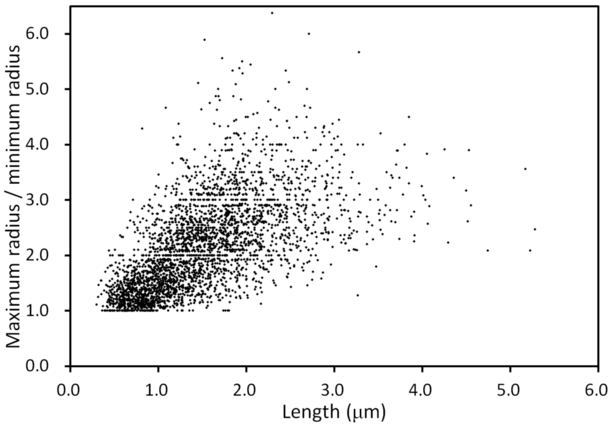


**k l**


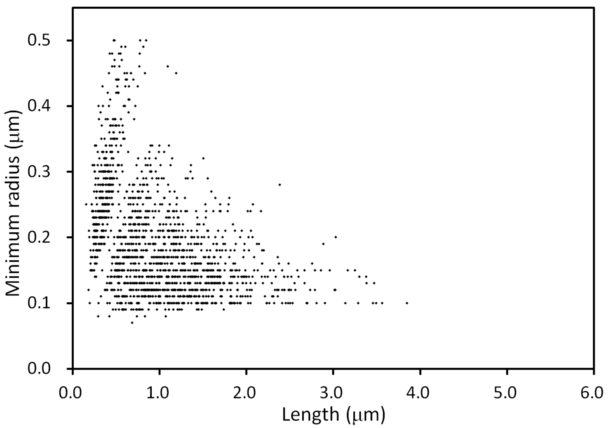

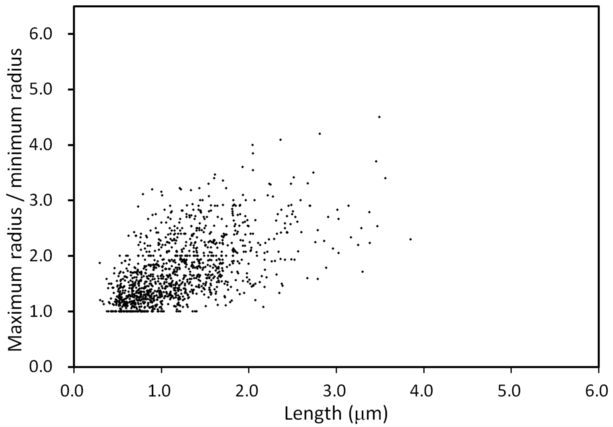


**Supplementary Figure S7 (cont'd).** Scatter plot of spine parameters. Minimum node radius of spine and ratio between maximum / minimum radii are plotted against spine length. (**g**) Minimum radius of N1. (**h**) Radius ratio of N1. (**i**) Minimum radius of N3. (**j**) Radius ratio of N3. (**k**) Minimum radius of N4. (**l**) Radius ratio of N4.

**a b c d**


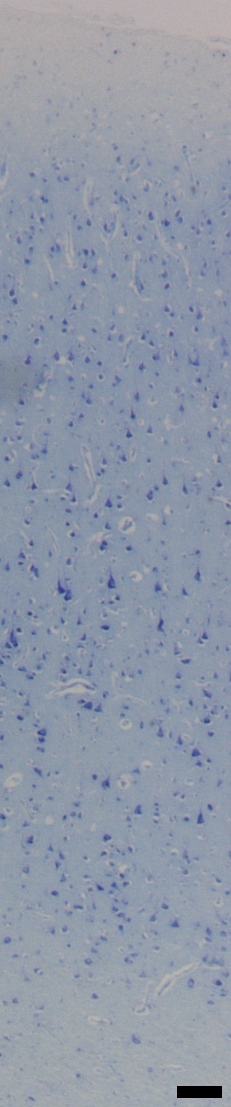

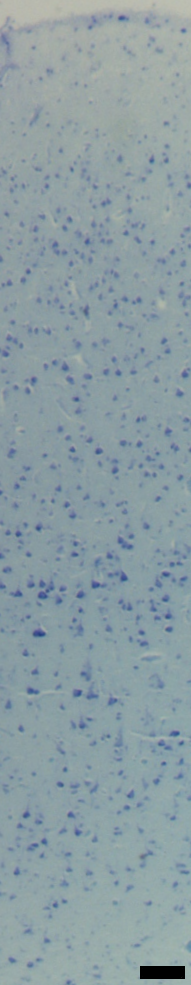

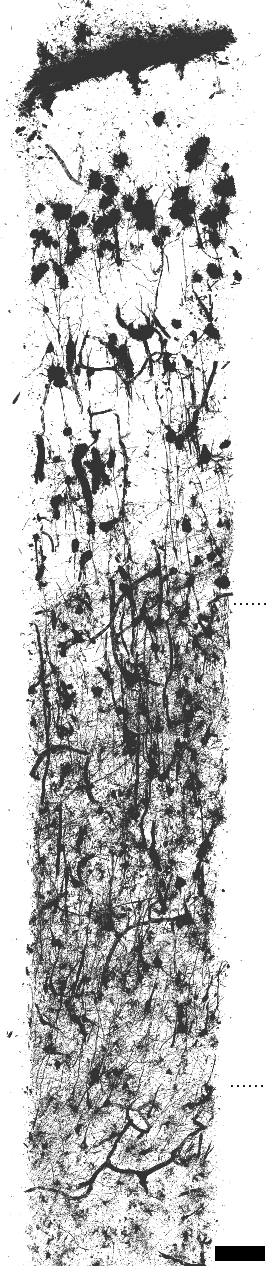

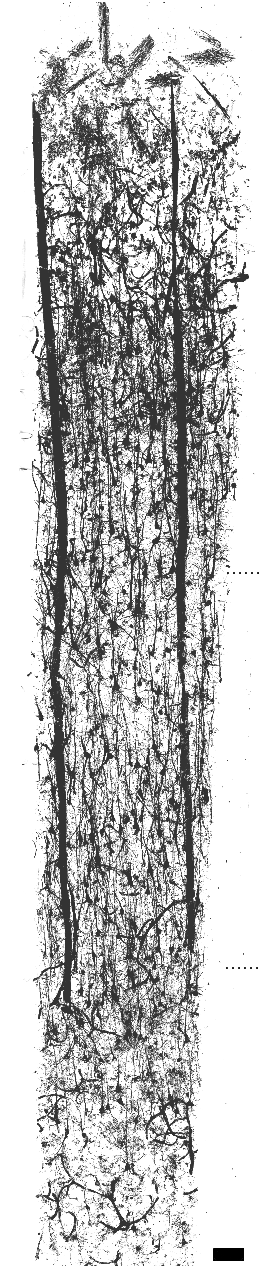


**Supplementary Figure S8.** Tissue images of S4 and N4 samples. Dot lines indicate layer V positions. Since paraffin-embed tissues showed shrinkage, Nissl sections were scaled so as to match the microtomographic image. Scale bars: 100 μm. (**a**) S4 structure visualized with microtomography. Linear attenuation coefficients of 37–56 cm^-1^ were rendered with the maximum projection method using the VG Studio software. (**b**) Nissl section of S4. (**c**) N4 structure. Linear attenuation coefficients of 36–56 cm^-1^ were rendered. (**d**) Nissl section of N4.

**a b**


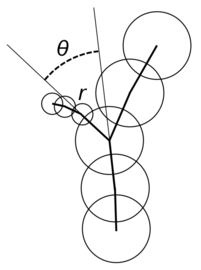

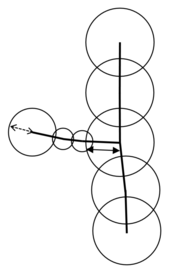


**c d**


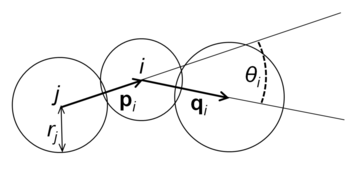

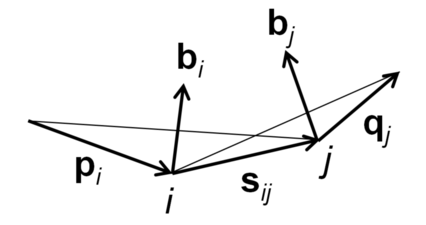


**Supplementary Figure S9.** Parameter definitions in geometry analysis. (**a**) Angle definition at ramification node. (**b**) Spine length was calculated by adding the end node radius (dashed arrow) and by subtracting the stem node radius (solid-line arrow) to obtain the protrusion length. (**c**) Vectors and parameters used in the curvature calculation. (**d**) Vectors used in the torsion calculation.

**Supplementary Table S4.** Geometric parameters of dendritic spines.

| Case | Curvature (μm^-1^) | Torsion (μm^-1^) | Length (μm) |
| --- | --- | --- | --- |
| S1 | 1.13 (0.50) / 4220 | -0.01 (1.74) / 3747 | 1.62 (0.82) / 4577 |
| S2 | 1.15 (0.52) / 2223 | 0.05 (1.99) / 1739 | 1.16 (0.70) / 2755 |
| S3 | 1.19 (0.57) / 859 | -0.08 (2.18) / 636 | 1.07 (0.69) / 1103 |
| S4 | 1.16 (0.49) / 5299 | 0.01 (1.92) / 4341 | 1.32 (0.92) / 6681 |
| N1 | 0.80 (0.54) / 162 | -0.14 (2.08) / 71 | 0.85 (0.80) / 296 |
| N2 | 1.18 (0.51) / 6023 | 0.02 (1.89) / 4986 | 1.30 (0.84) / 7393 |
| N3 | 1.16 (0.51) / 3124 | -0.03 (1.98) / 2705 | 1.36 (0.77) / 3634 |
| N4 | 1.12 (0.53) / 1187 | 0.09 (2.13) / 876 | 1.01 (0.60) / 1562 |

| Case | Maximum radius (μm) | Minimum radius (μm) |
| --- | --- | --- |
| S1 | 0.30 (0.09) | 0.18 (0.07) |
| S2 | 0.27 (0.08) | 0.17 (0.08) |
| S3 | 0.27 (0.08) | 0.19 (0.08) |
| S4 | 0.27 (0.08) | 0.17 (0.07) |
| N1 | 0.32 (0.12) | 0.27 (0.09) |
| N2 | 0.28 (0.07) | 0.17 (0.08) |
| N3 | 0.27 (0.08) | 0.16 (0.07) |
| N4 | 0.27 (0.08) | 0.19 (0.08) |

Values represent mean (sample standard deviation) / number of observations. Number of observations of the maximum and minimum radius are the same as those of the length.

**Supplementary Video captions**

**Supplementary Video S1.** Schizophrenia S4A structure. The structure rotates around the soma node of the PYR 2 pyramidal neuron. Structural constituents are color-coded. The pial surface is toward the top. Video frames were produced using the MCTrace software.

**Supplementary Video S2.** Control N4A structure. The structure rotates around the soma node of the PYR 3 pyramidal neuron. Structural constituents are color-coded. The pial surface is toward the top. Video frames were produced using the MCTrace software.
