# Supplementary material for: Three-dimensional alteration of neurites in schizophrenia
Source: Transl Psychiatry. 2019 Feb 12;9:85. doi: 10.1038/s41398-019-0427-4 (PMC6372695; doi:10.1038/s41398-019-0427-4)
Supplement: Supplementary file 4 — Supplementary Tables S1-S3, clean [file 41398_2019_427_MOESM4_ESM.pdf]

Supplementary Table S1. Statistics of schizophrenia and control cases.

Case code	S1	S2	S3	S4	N1	N2	N3	N4
Gender	female	female	male	male	female	female	male	male
Age	56	70	64	69	58	72	62	65
Postmortem interval (hour)	2	7.5	21	7	85	3.5	8	17.5
Psychiatric record	schizophrenia	schizophrenia	schizophrenia	schizophrenia	no	no	no	no
Layer V depth (um)	1600-2300	1500-2300	1600-2700	1100-2100	1700-2900	1000-1800	1300-2200	1700-2900
Number of datasets	5	10	10	4	10	4	8	4
Number of model nodes	43079	46760	27321	106820	10948	74813	45301	27992
Number of constituents	269	391	241	624	162	329	252	173
Pyramidal neurons	14	17	13	10	23	12	11	9
Interneurons	2	0	0	0	0	2	0	0
Non-typed neurons	3	4	3	0	6	2	0	1
Orphan neurites	240	352	214	607	112	310	230	162
Gliaform cells	1	9	2	5	4	1	10	0
Blood capillaries	9	9	9	2	17	2	1	1
Total length (um) [1]	15699.7	23796.3	13151.6	34948.0	11917.9	27662.9	20062.3	15131.5
Pyramidal process (um)	5852.1	8576.0	6141.4	6500.7	5895.3	9922.4	6972.1	6265.0
Interneuron process (um)	846.9	0.0	0.0	0.0	0.0	805.9	0.0	0.0
Non-typed neuron process (um)	183.2	555.7	185.2	0.0	457.7	227.1	0.0	326.1
Orphan neurite (um)	7790.3	12029.3	5727.0	24818.1	3267.5	15141.7	11944.9	8518.4
Gliaform cell process (um)	221.0	1834.6	549.4	3538.6	453.0	1185.5	1128.0	0.0
Blood capillary (um)	806.2	800.6	548.4	90.5	1844.1	380.4	17.1	22.1
Number of neurite segments	555	796	457	929	473	774	525	482
Number of spines	4577	2755	1103	6681	296	7393	3634	1562
Spine density (um^{-1}) [2]	0.422	0.171	0.136	0.261	0.078	0.323	0.247	0.132

[1] Spine length is not included.

[2] Spine density = number of spines / total length of spiny dendrite

Supplementary Table S2. Conditions of microtomography and nanotomography experiments.

Beamtime start date	2013.10.3	2014.5.27	2016.6.3	2017.4.23	2017.10.27	2011.12.8	2013.1.23 or later
Facility	SPring-8	SPring-8	APS	SPring-8	APS	SPring-8	SPring-8
Beamline	BL47XU	BL37XU	32-ID	BL37XU	32-ID	BL20XU [1]	BL20XU [1]
X-ray energy (keV)	8.0	8.0	8.0	8.0	8.0	12.0	12.0
Contrast	Zernike	Absorption	Zernike	Absorption	Zernike	Absorption	Absorption
Beam condenser	Sector zone plate	Sector zone plate	BSC [2]	Sector zone plate	CRL and BSC [2]	-	-
Outermost zone width (nm) / diameter (um) of Fresnel zone plate	100 / 155	100 / 310	60 / 180	100 / 310	60 / 180	-	-
Depth of focus (um) [3]	258	258	93	258	93	-	-
Scintillator screen	P43 (Gd_2O_2S:Tb)	P43	LuAG:Ce ($Lu_3Al_5O_{12}$:Ce)	P43	GGG:Eu ($Gd_3Ga_5O_{12}$:Eu)	LSO (Lu_2SiO_5:Ce)	LSO or LuAG:Ce
Pixel size (nm)	40.2	59.6	26.1	48.3	26.0	500	500
X-ray illumination (diameter in pixels)	1670	2400	2500	1900	2500	Full field	Full field
Viewing field (pixels) [4]	1680 x 1680	2048 x 2048	2448 x 2048	2048 x 2048	2448 x 2048	1920 x 1440	2048 x 2048
Maximum image width (um)	67	122	64	92	64	960	1024
Image dynamic range (bits) [5]	16	16	12	13	13	12	16
Number of sample frames per dataset	900	1800	1500	900 or 1800	2360	1800	1800
Degrees per frame	0.200	0.100	0.120	0.200 or 0.100	0.076	0.100	0.100
Exposure time per frame (msec)	500	700	1000	400	1000	200	100-150
Data collection time (sec)	600	2400	1800	600 or 1200	3000	1200	400-500
Spatial resolution (nm)	180 [6]	200 [6]	300 [6]	300 [6,7]	300 [6,7]	1200 [6]	1200 [6]

[1] Used for visualizing overall structures

[2] BSC: Beam Shaping Condenser; CRL: compound refractive lens

[3] Depth of focus Δf was calculated with $\Delta f = \pm \lambda / (2\ NA^2)$ and $NA = \lambda / (2\Delta r_N)$, where λ is the wavelength, NA is the numerical aperture, and Δr_N is the outermost zone width of the Fresnel zone plate.

[4] Width x height

[5] Defined from the maximum intensity of flat field images

[6] Determined using three-dimensional square-wave test patterns. These estimates represent the resolution that the instruments can reach.

[7] Determined from the Fourier domain plot. These estimates represent the resolution of the sample image itself.

Supplementary Table S3. Statistics of datasets and Cartesian coordinate models. (**a**) Schizophrenia case S1.

Dataset name	S1A	S1B	S1C	S1D	S1E
Beamtime start date	2013.10.3	2014.5.27	2014.5.27	2013.10.3	2014.5.27
Image size (pixel) [1]	1690 x 1690 x 4897	2670 x 2370 x 4408	2010 x 2010 x 4409	1710 x 1680 x 4897	2000 x 2000 x 3221
Image size (um) [1]	67.9 x 67.9 x 196.9	159.1 x 141.3 x 262.7	119.8 x 119.8 x 262.8	68.7 x 67.5 x 196.9	119.2 x 119.2 x 192.0
Cortical depth of upper end (um)	2300	1700	1200	1400	2300
Number of model nodes	2001	16507	17548	3611	3412
Number of constituents	16	112	96	35	10
Pyramidal neurons	1	3	6	3	1
Interneurons	0	0	1	1	0
Non-typed neurons	0	1	1	1	0
Orphan neurites	15	102	86	30	7
Gliaform cells	0	1	0	0	0
Blood capillaries	0	5	2	0	2
Total length (um) [2]	952.6	5145.9	6298.6	2031.6	1271.0
Pyramidal process (um)	498.4	1380.9	2655.1	716.6	601.1
Interneuron process (um)	0.0	0.0	501.8	345.1	0.0
Non-typed neuron process (um)	0.0	90.6	72.7	19.9	0.0
Orphan neurite (um)	454.2	3071.9	2873.5	950.1	440.6
Gliaform cell process (um)	0.0	221.0	0.0	0.0	0.0
Blood capillary (um)	0.0	381.5	195.5	0.0	229.2
Number of neurite segments	29	181	219	85	41
Number of spines	154	1812	1963	273	375
Spine density (um^{-1}) [3]	0.201	0.471	0.469	0.227	0.443

[1] Image width x height x number of slices

[2] Spine length is not included.

[3] Spine density = number of spines / total length of spiny dendrite

Supplementary Table S3. Statistics of datasets and Cartesian coordinate models. (**b**) Schizophrenia case S2.

Dataset name	S2A	S2B	S2C	S2D	S2E	S2F
Beamtime start date	2017.4.23	2017.4.23	2017.4.23	2017.4.23	2017.4.23	2017.10.27
Image size (pixel) [1]	2020 x 2040 x 4550	2020 x 2020 x 4554	2030 x 2040 x 7053	2010 x 2020 x 4554	2000 x 2000 x 4550	1180 x 1180 x 2533
Image size (um) [1]	97.6 x 98.5 x 219.8	97.6 x 97.6 x 220.0	98.0 x 98.5 x 340.7	97.1 x 97.6 x 220.0	96.6 x 96.6 x 219.8	61.4 x 61.4 x 131.7
Cortical depth of upper end (um)	1800	1900	1800	2000	2300	2300
Number of model nodes	3033	10799	8673	12982	3330	1149
Number of constituents	20	82	57	79	34	30
Pyramidal neurons	1	4	3	2	1	1
Interneurons	0	0	0	0	0	0
Non-typed neurons	0	2	0	1	0	1
Orphan neurites	18	75	51	67	31	27
Gliaform cells	0	0	1	7	0	0
Blood capillaries	1	1	2	2	2	1
Total length (um) [2]	1632.1	4940.7	4405.7	6453.0	1480.6	668.5
Pyramidal process (um)	866.1	1387.8	2065.9	1618.4	409.6	128.3
Interneuron process (um)	0.0	0.0	0.0	0.0	0.0	0.0
Non-typed neuron process (um)	0.0	397.6	0.0	140.8	0.0	17.3
Orphan neurite (um)	755.5	3134.9	2051.0	2641.7	828.7	501.9
Gliaform cell process (um)	0.0	0.0	0.0	1834.6	0.0	0.0
Blood capillary (um)	10.5	20.4	288.8	217.5	242.4	21.0
Number of neurite segments	55	158	135	152	53	45
Number of spines	167	641	590	791	270	38
Spine density (um^{-1}) [3]	0.121	0.170	0.178	0.209	0.250	0.159

[1] Image width x height x number of slices

[2] Spine length is not included.

[3] Spine density = number of spines / total length of spiny dendrite

Supplementary Table S3. Statistics of datasets and Cartesian coordinate models. (**b**) Schizophrenia case S2 (cont'd).

Dataset name	S2G	S2H	S2I	S2J
Beamtime start date	2017.10.27	2017.10.27	2017.10.27	2017.10.27
Image size (pixel) [1]	1180 x 1170 x 4217	1190 x 1190 x 2879	1080 x 1080 x 4400	1200 x 1200 x 3358
Image size (um) [1]	61.4 x 60.8 x 219.3	61.9 x 61.9 x 149.7	56.2 x 56.2 x 228.8	62.4 x 62.4 x 174.6
Cortical depth of upper end (um)	2100	2100	1900	1800
Number of model nodes	3269	1281	1848	396
Number of constituents	46	13	22	8
Pyramidal neurons	2	1	1	1
Interneurons	0	0	0	0
Non-typed neurons	0	0	0	0
Orphan neurites	44	12	20	7
Gliaform cells	0	0	1	0
Blood capillaries	0	0	0	0
Total length (um) [2]	1977.2	758.3	1082.8	397.4
Pyramidal process (um)	1030.8	319.0	539.0	211.1
Interneuron process (um)	0.0	0.0	0.0	0.0
Non-typed neuron process (um)	0.0	0.0	0.0	0.0
Orphan neurite (um)	946.4	439.3	543.7	186.2
Gliaform cell process (um)	0.0	0.0	0.0	0.0
Blood capillary (um)	0.0	0.0	0.0	0.0
Number of neurite segments	91	35	51	21
Number of spines	84	90	68	16
Spine density (um^{-1}) [3]	0.071	0.151	0.106	0.198

[1] Image width x height x number of slices

[2] Spine length is not included.

[3] Spine density = number of spines / total length of spiny dendrite

Supplementary Table S3. Statistics of datasets and Cartesian coordinate models. (**c**) Schizophrenia case S3.

Dataset name	S3A	S3B	S3C	S3D	S3E	S3F
Beamtime start date	2017.10.27	2017.10.27	2017.10.27	2017.10.27	2017.10.27	2017.4.23
Image size (pixel) [1]	1190 x 1180 x 1994	1180 x 1180 x 1864	1180 x 1180 x 4223	1180 x 1170 x 2550	1210 x 1210 x 2546	2020 x 2020 x 4555
Image size (um) [1]	61.9 x 61.4 x 103.7	61.4 x 61.4 x 96.9	61.4 x 61.4 x 219.6	61.4 x 60.8 x 132.6	62.9 x 62.9 x 132.4	97.6 x 97.6 x 220.0
Cortical depth of upper end (um)	1400	1600	1600	2000	1400	2300
Number of model nodes	1351	502	2723	1504	2691	4965
Number of constituents	11	9	21	11	30	58
Pyramidal neurons	1	1	1	1	1	2
Interneurons	0	0	0	0	0	0
Non-typed neurons	0	0	0	0	0	0
Orphan neurites	10	8	20	9	27	53
Gliaform cells	0	0	0	1	1	0
Blood capillaries	0	0	0	0	1	3
Total length (um) [2]	617.8	296.3	1270.5	734.2	1652.2	2213.1
Pyramidal process (um)	411.3	150.3	589.9	364.9	452.9	577.7
Interneuron process (um)	0.0	0.0	0.0	0.0	0.0	0.0
Non-typed neuron process (um)	0.0	0.0	0.0	0.0	0.0	0.0
Orphan neurite (um)	206.4	145.9	680.6	276.1	721.4	1424.6
Gliaform cell process (um)	0.0	0.0	0.0	93.2	456.2	0.0
Blood capillary (um)	0.0	0.0	0.0	0.0	21.7	210.8
Number of neurite segments	26	17	44	23	47	86
Number of spines	22	5	151	87	39	184
Spine density (um^{-1}) [3]	0.081	0.034	0.201	0.193	0.083	0.133

[1] Image width x height x number of slices

[2] Spine length is not included.

[3] Spine density = number of spines / total length of spiny dendrite

Supplementary Table S3. Statistics of datasets and Cartesian coordinate models. (**c**) Schizophrenia case S3 (cont'd).

Dataset name	S3G	S3H	S3I	S3J
Beamtime start date	2017.4.23	2017.4.23	2017.4.23	2014.5.27
Image size (pixel) [1]	2020 x 2020 x 7061	2020 x 2020 x 5810	2000 x 2010 x 5810	2030 x 2030 x 3227
Image size (um) [1]	97.6 x 97.6 x 341.0	97.6 x 976. x 280.6	96.6 x 97.1 x 280.6	121.0 x 121.0 x 192.3
Cortical depth of upper end (um)	2300	2300	2400	3300
Number of model nodes	5082	3586	1027	3890
Number of constituents	38	27	7	29
Pyramidal neurons	2	2	1	1
Interneurons	0	0	0	0
Non-typed neurons	0	1	0	2
Orphan neurites	35	23	5	24
Gliaform cells	0	0	0	0
Blood capillaries	1	1	1	2
Total length (um) [2]	2153.5	1747.2	624.9	1841.9
Pyramidal process (um)	1295.1	866.3	480.2	952.8
Interneuron process (um)	0.0	0.0	0.0	0.0
Non-typed neuron process (um)	0.0	49.6	0.0	135.6
Orphan neurite (um)	805.0	737.2	107.2	622.6
Gliaform cell process (um)	0.0	0.0	0.0	0.0
Blood capillary (um)	53.4	94.0	37.6	130.9
Number of neurite segments	71	62	21	60
Number of spines	330	107	45	133
Spine density (um^{-1}) [3]	0.182	0.089	0.086	0.119

[1] Image width x height x number of slices

[2] Spine length is not included.

[3] Spine density = number of spines / total length of spiny dendrite

Supplementary Table S3. Statistics of datasets and Cartesian coordinate models. (**d**) Schizophrenia case S4.

Dataset name	S4A	S4B	S4C	S4D
Beamtime start date	2017.4.23	2017.4.23	2017.4.23	2017.4.23
Image size (pixel) [1]	2030 x 2020 x 4552	2020 x 2020 x 4549	2010 x 2010 x 3294	2030 x 2020 x 4549
Image size (um) [1]	98.0 x 97.6 x 219.9	97.6 x 97.6 x 219.7	97.1 x 97.1 x 159.1	98.0 x 97.6 x 219.7
Cortical depth of upper end (um)	1600	1900	2000	2000
Number of model nodes	25841	35936	26500	18543
Number of constituents	188	184	145	107
Pyramidal neurons	1	3	3	3
Interneurons	0	0	0	0
Non-typed neurons	0	0	0	0
Orphan neurites	186	177	141	103
Gliaform cells	0	4	1	0
Blood capillaries	1	0	0	1
Total length (um) [2]	7916.0	12665.9	8650.1	5716.0
Pyramidal process (um)	719.2	2030.5	2442.8	1308.2
Interneuron process (um)	0.0	0.0	0.0	0.0
Non-typed neuron process (um)	0.0	0.0	0.0	0.0
Orphan neurite (um)	7163.0	7717.9	5586.2	4351.0
Gliaform cell process (um)	0.0	2917.4	621.2	0.0
Blood capillary (um)	33.8	0.0	0.0	56.7
Number of neurite segments	260	279	243	147
Number of spines	1304	2297	1843	1237
Spine density (um^{-1}) [3]	0.225	0.277	0.271	0.263

[1] Image width x height x number of slices

[2] Spine length is not included.

[3] Spine density = number of spines / total length of spiny dendrite

Supplementary Table S3. Statistics of datasets and Cartesian coordinate models. (**e**) Control case N1.

Dataset name	N1A	N1B	N1C	N1D	N1E	N1F
Beamtime start date	2016.6.3	2016.6.3	2016.6.3	2016.6.3	2017.4.23	2017.4.23
Image size (pixel) [1]	1224 x 1224 x 1940	1224 x 1224 x 2873	1224 x 1224 x 2852	1224 x 1224 x 3772	2040 x 2040 x 5815	2070 x 2100 x 4537
Image size (um) [1]	63.9 x 63.9 x 101.3	63.9 x 63.9 x 150.0	63.9 x 63.9 x 148.9	63.9 x 63.9 x 196.9	98.5 x 98.5 x 280.9	100.0 x 101.4 x 219.1
Cortical depth of upper end (um)	2800	2600	2400	2200	1200	1500
Number of model nodes	235	984	1378	1421	944	1444
Number of constituents	7	20	11	8	16	24
Pyramidal neurons	2	2	1	1	2	2
Interneurons	0	0	0	0	0	0
Non-typed neurons	0	0	0	0	0	1
Orphan neurites	4	15	8	5	13	16
Gliaform cells	0	0	0	0	0	0
Blood capillaries	1	3	2	2	1	5
Total length (um) [2]	390.0	898.4	876.2	1252.1	1060.1	1590.9
Pyramidal process (um)	236.3	397.5	500.0	881.3	540.9	601.9
Interneuron process (um)	0.0	0.0	0.0	0.0	0.0	0.0
Non-typed neuron process (um)	0.0	0.0	0.0	0.0	0.0	104.5
Orphan neurite (um)	75.1	427.0	278.0	87.9	388.8	484.1
Gliaform cell process (um)	0.0	0.0	0.0	0.0	0.0	0.0
Blood capillary (um)	78.6	73.9	98.2	283.0	130.4	400.3
Number of neurite segments	17	57	35	40	47	58
Number of spines	8	40	2	1	62	85
Spine density (um^{-1}) [3]	0.064	0.102	0.014	0.007	0.092	0.124

[1] Image width x height x number of slices

[2] Spine length is not included.

[3] Spine density = number of spines / total length of spiny dendrite

Supplementary Table S3. Statistics of datasets and Cartesian coordinate models. (**e**) Control case N1 (cont'd).

Dataset name	N1G	N1H	N1I	N1J
Beamtime start date	2017.4.23	2017.10.27	2017.10.27	2017.10.27
Image size (pixel) [1]	2030 x 2090 x 5802	1200 x 1200 x 3381	1200 x 1190 x 3372	1220 x 1210 x 3377
Image size (um) [1]	98.0 x 100.9 x 280.2	62.4 x 62.4 x 175.8	62.4 x 61.9 x 175.3	63.4 x 62.9 x 175.6
Cortical depth of upper end (um)	1700	2000	2200	2400
Number of model nodes	1654	1112	873	903
Number of constituents	15	31	19	11
Pyramidal neurons	3	2	6	2
Interneurons	0	0	0	0
Non-typed neurons	1	2	1	1
Orphan neurites	8	26	10	7
Gliaform cells	1	1	2	0
Blood capillaries	2	0	0	1
Total length (um) [2]	2478.1	1172.3	1251.8	948.0
Pyramidal process (um)	1028.0	189.5	851.3	668.6
Interneuron process (um)	0.0	0.0	0.0	0.0
Non-typed neuron process (um)	48.7	148.1	33.4	123.0
Orphan neurite (um)	259.2	834.6	360.4	72.4
Gliaform cell process (um)	446.4	0.0	6.6	0.0
Blood capillary (um)	695.7	0.0	0.0	84.0
Number of neurite segments	66	48	61	44
Number of spines	68	20	5	5
Spine density (um^{-1}) [3]	0.088	0.035	0.076	0.022

[1] Image width x height x number of slices

[2] Spine length is not included.

[3] Spine density = number of spines / total length of spiny dendrite

Supplementary Table S3. Statistics of datasets and Cartesian coordinate models. (**f**) Control case N2.

Dataset name	N2A	N2B	N2C	N2D
Beamtime start date	2017.4.23	2017.4.23	2017.4.23	2017.4.23
Image size (pixel) [1]	2050 x 2040 x 5808	2030 x 2030 x 5803	2040 x 2040 x 5809	2040 x 2040 x 4553
Image size (um) [1]	99.0 x 98.5 x 280.5	98.0 x 98.0 x 280.3	98.5 x 98.5 x 280.6	98.5 x 98.5 x 219.9
Cortical depth of upper end (um)	1700	1300	1400	1400
Number of model nodes	4955	34444	20156	15258
Number of constituents	7	141	83	98
Pyramidal neurons	1	5	3	3
Interneurons	0	2	0	0
Non-typed neurons	0	2	0	0
Orphan neurites	4	131	80	95
Gliaform cells	0	1	0	0
Blood capillaries	2	0	0	0
Total length (um) [2]	2658.2	12523.1	6685.6	5796.0
Pyramidal process (um)	1905.8	3669.0	2549.8	1797.8
Interneuron process (um)	0.0	805.9	0.0	0.0
Non-typed neuron process (um)	0.0	227.1	0.0	0.0
Orphan neurite (um)	372.0	6635.6	4135.9	3998.2
Gliaform cell process (um)	0.0	1185.5	0.0	0.0
Blood capillary (um)	380.4	0.0	0.0	0.0
Number of neurite segments	64	340	174	196
Number of spines	430	3591	2046	1326
Spine density (um^{-1}) [3]	0.209	0.365	0.341	0.266

[1] Image width x height x number of slices

[2] Spine length is not included.

[3] Spine density = number of spines / total length of spiny dendrite

Supplementary Table S3. Statistics of datasets and Cartesian coordinate models. (**g**) Control case N3.

Dataset name	N3A	N3B	N3C	N3D	N3E	N3F
Beamtime start date	2016.6.3	2017.4.23	2017.4.23	2017.4.23	2017.10.27	2017.10.27
Image size (pixel) [1]	1224 x 1224 x 8440	2020 x 2020 x 7060	1990 x 2000 x 7050	2020 x 2000 x 8311	1230 x 1220 x 4217	1160 x 1150 x 2702
Image size (um) [1]	63.9 x 63.9 x 440.6	97.6 x 97.6 x 341.0	96.1 x 96.6 x 340.5	97.6 x 96.6 x 401.4	64.0 x 63.4 x 219.3	60.3 x 59.8 x 140.5
Cortical depth of upper end (um)	1600	1400	1500	1900	1800	2100
Number of model nodes	3095	11978	4633	11680	4516	1069
Number of constituents	36	62	29	56	23	10
Pyramidal neurons	1	3	1	1	2	1
Interneurons	0	0	0	0	0	0
Non-typed neurons	0	0	0	0	0	0
Orphan neurites	35	49	28	55	20	9
Gliaform cells	0	9	0	0	1	0
Blood capillaries	0	1	0	0	0	0
Total length (um) [2]	1995.1	5862.3	2258.8	4109.4	2056.2	679.4
Pyramidal process (um)	759.0	1492.4	685.6	1217.0	1014.6	534.9
Interneuron process (um)	0.0	0.0	0.0	0.0	0.0	0.0
Non-typed neuron process (um)	0.0	0.0	0.0	0.0	0.0	0.0
Orphan neurite (um)	1236.1	3308.7	1573.2	2892.5	957.5	144.5
Gliaform cell process (um)	0.0	1044.0	0.0	0.0	84.0	0.0
Blood capillary (um)	0.0	17.1	0.0	0.0	0.0	0.0
Number of neurite segments	65	119	56	97	59	28
Number of spines	37	1096	298	914	340	76
Spine density (um^{-1}) [3]	0.058	0.257	0.177	0.259	0.204	0.171

[1] Image width x height x number of slices

[2] Spine length is not included.

[3] Spine density = number of spines / total length of spiny dendrite

Supplementary Table S3. Statistics of datasets and Cartesian coordinate models. (**g**) Control case N3 (cont'd).

Dataset name	N3G	N3H
Beamtime start date	2017.10.27	2017.10.27
Image size (pixel) [1]	1170 x 1170 x 3535	1230 x 1230 x 3378
Image size (um) [1]	60.8 x 60.8 x 183.8	64.0 x 64.0 x 175.7
Cortical depth of upper end (um)	2300	1700
Number of model nodes	5424	2906
Number of constituents	24	12
Pyramidal neurons	1	1
Interneurons	0	0
Non-typed neurons	0	0
Orphan neurites	23	11
Gliaform cells	0	0
Blood capillaries	0	0
Total length (um) [2]	2072.1	1029.0
Pyramidal process (um)	646.9	621.7
Interneuron process (um)	0.0	0.0
Non-typed neuron process (um)	0.0	0.0
Orphan neurite (um)	1425.1	407.3
Gliaform cell process (um)	0.0	0.0
Blood capillary (um)	0.0	0.0
Number of neurite segments	69	32
Number of spines	620	253
Spine density (um^{-1}) [3]	0.342	0.369

[1] Image width x height x number of slices

[2] Spine length is not included.

[3] Spine density = number of spines / total length of spiny dendrite

Supplementary Table S3. Statistics of datasets and Cartesian coordinate models. (**h**) Control case N4.

Dataset name	N4A	N4B	N4C	N4D
Beamtime start date	2017.4.23	2017.4.23	2017.4.23	2017.4.23
Image size (pixel) [1]	2040 x 2040 x 7051	2060 x 2040 x 4552	2050 x 2050 x 5786	2000 x 2010 x 3299
Image size (um) [1]	98.5 x 98.5 x 340.6	99.5 x 98.5 x 219.9	99.0 x 99.0 x 279.5	96.6 x 97.1 x 159.3
Cortical depth of upper end (um)	1400	1800	2200	2700
Number of model nodes	12016	5904	6579	3493
Number of constituents	76	25	53	19
Pyramidal neurons	2	3	3	1
Interneurons	0	0	0	0
Non-typed neurons	1	0	0	0
Orphan neurites	73	22	49	18
Gliaform cells	0	0	0	0
Blood capillaries	0	0	1	0
Total length (um) [2]	5979.6	3240.7	3912.5	1998.7
Pyramidal process (um)	2153.7	1969.9	1354.6	786.8
Interneuron process (um)	0.0	0.0	0.0	0.0
Non-typed neuron process (um)	326.1	0.0	0.0	0.0
Orphan neurite (um)	3499.8	1270.8	2535.9	1211.9
Gliaform cell process (um)	0.0	0.0	0.0	0.0
Blood capillary (um)	0.0	0.0	22.1	0.0
Number of neurite segments	192	96	134	60
Number of spines	603	514	353	92
Spine density (um^{-1}) [3]	0.126	0.201	0.112	0.070

[1] Image width x height x number of slices

[2] Spine length is not included.

[3] Spine density = number of spines / total length of spiny dendrite
